# Human metabolism and urinary excretion of seven neonicotinoids and neonicotinoid-like compounds after controlled oral dosages

**DOI:** 10.1007/s00204-021-03159-0

**Published:** 2021-10-13

**Authors:** Sonja A. Wrobel, Daniel Bury, Heiko Hayen, Holger M. Koch, Thomas Brüning, Heiko U. Käfferlein

**Affiliations:** 1 Institute for Prevention and Occupational Medicine of the German Social Accident Insurance (IPA), Ruhr-University Bochum, Bürkle-de-la-Camp-Platz 1, 44789 Bochum, Germany; 2 Institute of Inorganic and Analytical Chemistry, University of Münster, Corrensstraße 30, 48149 Münster, Germany

**Keywords:** Neonicotinoids, Metabolism, Metabolites, Urine, Metabolite screening

## Abstract

Few human data on exposure and toxicity are available on neonicotinoids and neonicotinoid-like compounds (NNIs), an important group of insecticides worldwide. Specifically, exposure assessment of humans by biomonitoring remains a challenge due to the lack of appropriate biomarkers. We investigated the human metabolism and metabolite excretion in urine of acetamiprid (ACE), clothianidin (CLO), flupyradifurone (FLUP), imidacloprid (IMI), sulfoxaflor (SULF), thiacloprid (THIAC) and thiamethoxam (THIAM) after single oral dosages at the currently acceptable daily intake levels of the European Food Safety Authority. Consecutive post-dose urine samples were collected up to 48 h. Suspect screening of tentative metabolites was carried out by liquid chromatography–high-resolution mass spectrometry. Screening hits were identified based on their accurate mass, isotope signal masses and ratios, product ion spectra, and excretion kinetics. We found, with the exception of SULF, extensive metabolization of NNIs to specific metabolites which were excreted next to the parent compounds. Overall, 24 metabolites were detected with signal intensities indicative of high metabolic relevance. Phase-I metabolites were predominantly derived by mono-oxidation (such as hydroxy-FLUP, -IMI, and -THIAC) and by oxidative *N*-desalkylation (such as *N*-desdifluoroethyl-FLUP and *N*-desmethyl-ACE, -CLO and -THIAM). IMI-olefin, obtained by dehydration of hydroxylated IMI, was identified as a major metabolite of IMI. SULF was excreted unchanged in urine. Previously reported metabolites of NNIs such as 6-chloronicotinic acid or 2-chlorothiazole-4-carboxylic acid and their glycine derivatives were detected either at low signal intensities or not at all and seem less relevant for human biomonitoring. Our highly controlled approach provides specific insight into the human metabolism of NNIs and suggests suitable biomarkers for future exposure assessment at environmentally relevant exposures.

**Supplementary Information:**

The online version contains supplementary material available at 10.1007/s00204-021-03159-0.

## Introduction

Neonicotinoids and neonicotinoid-like compounds (NNIs) are used as systemic insecticides in plant protection and veterinary products and act by blocking or modulating the nicotinic acetylcholine receptors (nAChR) of insects (Casida and Durkin 2013; Casida 2018). Due to their low acute toxicity in mammals, NNIs are considered safe alternatives to pyrethroids and carbamates (Simon-Delso et al. 2015). However, in recent years, selected NNIs have received widespread attention due to their proven sublethal behavioral effects on pollinators (Goulson 2015; Woodcock et al. 2017; Bell et al. 2020; Crall et al. 2018). Therefore, the ecotoxicological impact of neonicotinoids next to pyrethroids is increasingly considered critical (Schulz et al. 2021). In contrast, reports on chronic neurotoxicity and behavioral toxicity in mammals including humans (in vivo/in vitro) remain scarce (Calderón-Segura et al. 2012; Simon-Delso et al. 2015; Kimura-Kuroda et al. 2012; Yang et al. 2014; Marfo et al. 2015; Loser et al. 2021).

**Fig. 1:**
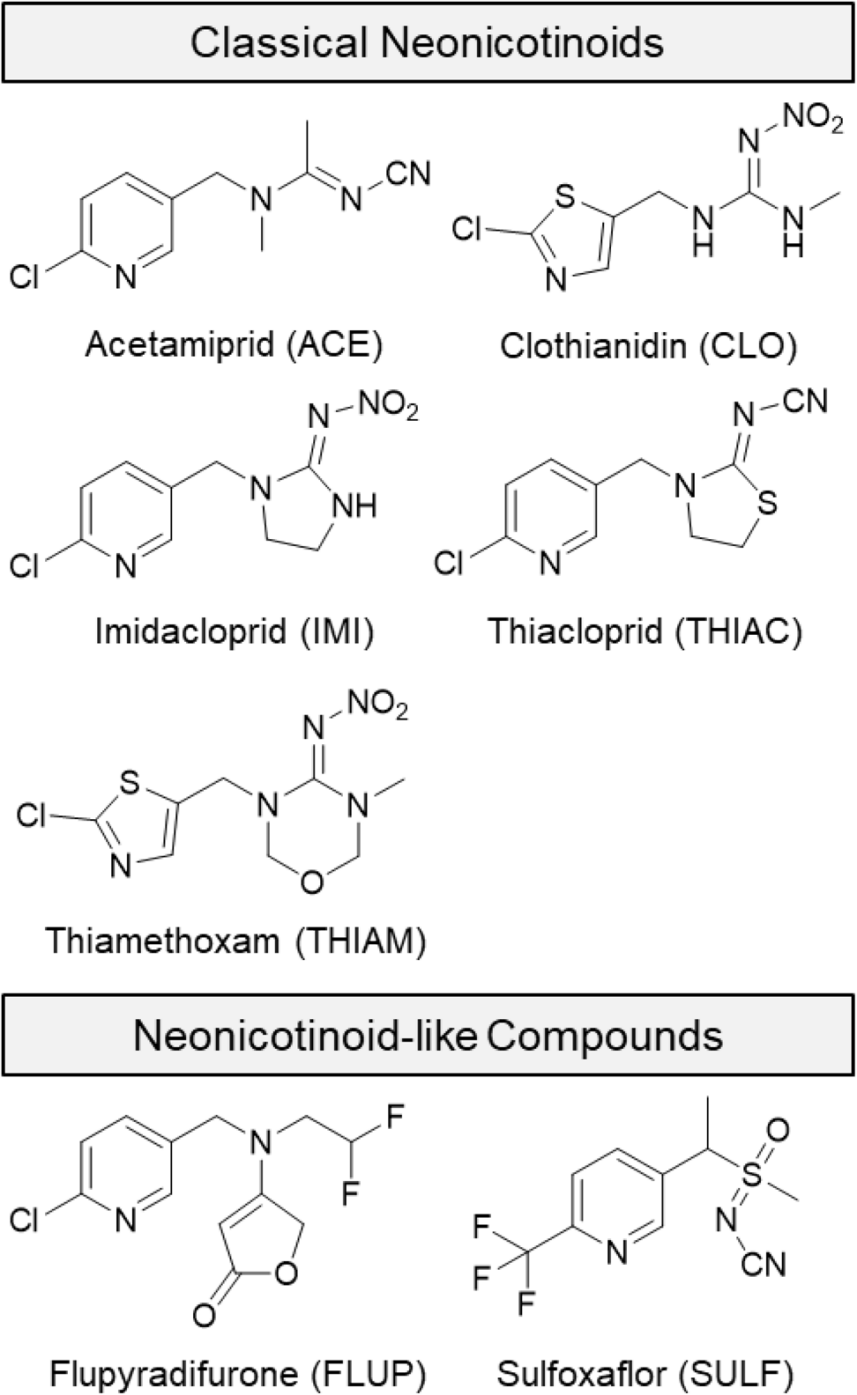
Structural overview on classical neonicotinoids and neonicotinoid-like compounds which have been studied

NNIs were developed during the late 1980s and the first compound was imidacloprid (IMI) (Shiokawa et al. 1986; Kagabu et al. 1985; Moriya et al. 1992). In the following years, structurally related substances were developed which also specifically block the nAChR receptor in insects including acetamiprid (ACE), clothianidin (CLO), thiacloprid (THIAC), and thiamethoxam (THIAM) (Tomizawa and Casida 2011; Kagabu 2011). The most recently developed compounds such as the butenolide flupyradifurone (FLUP) (Jeschke et al. 2006) and the sulfoximine sulfoxaflor (SULF) (Loso et al. 2007) are also known nAChR agonists and show similar toxicity (Siviter and Muth 2020) although being not completely identical to the classical neonicotinoids with regard to their structure–activity relationship (Nauen et al. 2015; Sparks et al. 2013) (Fig. 1) and thus are neonicotinoid-like compounds. All in all, NNIs represent the world’s fastest growing and largest selling group of insecticides for agricultural and veterinary use (Jeschke et al. 2011; Craddock et al. 2019; Bass et al. 2015).

Due to their extensive use in agriculture and households, NNIs have become ubiquitous in the environment and are found in water and soil (Dankyi et al. 2014; Morrissey et al. 2015). In addition, NNIs have been detected in food such as fruits, vegetables, milk, and honey (Taira 2014; Lu et al. 2018; Lachat and Glauser 2018; Mitchell et al. 2017). However, actual data on human exposure, specifically biomonitoring data, remain scarce (Cimino et al. 2017), most likely due to the lack of specific biomarkers and basic metabolic data in humans. Consequently, with the exception of IMI (Ospina et al. 2019; Wrobel et al. 2020; Song et al. 2021), only few information is available on the excretion of specific metabolites of other NNIs in humans, e.g., *N*-demethylated ACE in urine (Taira et al. 2013; Ospina et al. 2019) or in saliva and blood (Zhang et al. 2021). In addition, it is difficult to extrapolate data to humans from experimental studies such as in rodents due to significant interspecies differences in the metabolism of NNIs (Khidkhan et al. 2021). Therefore, the overall aim of this study was to identify promising and specific human biomarkers of NNIs which, in the future, can be used for exposure assessment and population monitoring. For this purpose, we specifically investigated the metabolism of five classical neonicotinoids (ACE, CLO, IMI, THIAC, THIAM) and two of their major neonicotinoid-like substitution products (FLUP, SULF) after controlled single oral dosages by high-performance liquid chromatography and high-resolution mass spectrometry. We also determined the urinary excretion kinetics of the parent compounds and their metabolites in consecutively collected urine samples up to 48 h.

ACE, CLO, FLUP, IMI, SULF, THIAC and THIAM were intentionally chosen for our studies. Because NNIs are systemic insecticides and are distributed throughout the plant including their nectaries (Simon-Delso et al. 2015), the current global application and geographic distribution of the different types of NNIs are almost perfectly reflected in their occurrence in honey samples. In example, analyses of honey samples from different parts of the world showed that ACE, CLO, IMI, THIAC and THIAM are by far the most important NNIs worldwide even if distinctive geographical differences can be identified depending on their current or pending registration status as an insecticide (Mitchell et al. 2017). In addition, the neonicotinoid-like butenolide FLUP (Nauen et al. 2015) and the sulfoximine SULF (Sparks et al. 2013) are considered the most important substitution products for IMI, CLO, and THIAM. The latter have recently been banned for outdoor use in the European Union due to their sublethal effects on pollinators (Crall et al. 2018; Tsvetkov et al. 2017; Woodcock et al. 2017). By administering single doses of NNIs one by one and using doses at the acceptable daily intake (ADI), our approach allowed to specifically assign metabolites to certain NNIs, evaluate the excretion kinetics of NNIs and their metabolites in detail, and guarantee that the results are of high relevance for environmental exposures in humans.

## Materials and methods

### Chemicals and reagents

Acetamiprid, clothianidin, flupyradifurone, imidacloprid, imidacloprid-olefin, thiacloprid, thiamethoxam, 6-chloronicotinic acid (Pestanal^®^ analytical standards), ammonium acetate (BioXtra, ≥ 98%), and β-Glucuronidase/sulfatase (*Helix pomatia*, type HP2; ≥ 100,000/ ≤ 7500 U/ml, pH 5) were purchased from Sigma-Aldrich (Steinheim, Germany). Sulfoxaflor and 2-chlorothiazole-4-carboxylic acid was purchased from TRC (North York, Ontario, Canada). *N*-Desmethyl-acetamiprid and clothianidin-desmethyl were purchased from LGC (Augsburg, Germany). 4-Hydroxyimidacloprid was purchased from Eurisotop (Saarbrücken, Germany). 5-Hydroxyimidacloprid (CAS: 155802-61-2) was purchased from Dr. Gilsing, Institute of Thin Film and Microsensoric Technology (Teltow, Germany) and Eurisotope. Ethanol (≥ 99.8%), water (LC–MS grade), acetonitrile (CHROMASOLV™) and formic acid (≥ 98%) were purchased from Honeywell (Seelze, Germany). Acetic acid (100% for LC–MS) was purchased from Merck (Darmstadt, Germany). Polyethylene containers (250 and 500 mL) were purchased from Sarstedt (Nümbrecht, Germany). Screw neck vials (glass, 1.5 mL) were purchased from Macherey–Nagel (Düren, Germany). Vial inserts (400 µL, glass, flat bottom) were purchased from Agilent (Waldbronn, Germany). Screw caps (PP, 9 mm) were purchased from VWR (Darmstadt, Germany).

### Dose studies

Dose studies were conducted for each of the seven NNIs in a single volunteer (male, Caucasian, 50 years, 83 kg body weight, non-smoker). The doses were applied orally. The time interval between the single doses was at least 4 weeks. The respective doses of each NNI were based on the acceptable daily intake (ADI) proposed by the European Food Safety Authority (EFSA) (Table 1). The doses were prepared in 1 mL ethanol, diluted with 5 mL water and were administered in an edible chocolate cup in the morning together with a light breakfast. The orally applied final doses were between 1 (thiacloprid) and 5 mg (e.g., imidacloprid) depending on the respective NNI. Post-dose urine samples were collected consecutively and completely up to 48 h in 250 or 500 mL polyethylene containers. Time and volume (gravimetrically) of each urine sample was recorded. All samples were stored frozen at − 20 °C until further use. The first urine sample (full void) was provided just before dosing (*t* = 0). Urinary creatinine concentrations were determined by the Jaffé method (L.u.P GmbH Labor- und Praxis Service, Bochum, Germany). All experiments were carried out after informed written consent and have been reviewed by the Ethics Committee of the Ruhr-University Bochum, Germany (IRB Reg. No. 18-6680-BR).

**Table 1:** Oral doses based on the acceptable daily intakes (ADI in mg/kg/day) by the European Food Safety Authority (European Commission 2016) for neonicotinoids and neonicotinoid-like compounds (NNI)

NNI	ADI [mg/kg/day]	*D*_C_ [mg]	*D*_A_ [mg]
Acetamiprid	0.025	2.00	2
Clothianidin	0.097	7.76	5
Flupyradifurone	0.064	5.12	5
Imidacloprid	0.060	4.80	5
Sulfoxaflor	0.040	3.20	3
Thiacloprid	0.010	0.80	1
Thiamethoxam	0.026	2.08	2

The doses were calculated (*D*_C_) for a volunteer of 80 kg body weight. The finally applied oral doses (*D*_A_) were rounded and limited to a maximum of 5 mg

### Sample preparation

Frozen samples were equilibrated to room temperature. 300 µL aliquots of homogenized samples were transferred into glass screw-cap vials and 100 µL ammonium acetate buffer (1 M, pH 6.2–6.4) was added. A volume of 6 µL of a β-glucuronidase/arylsulfatase solution was added for possible deconjugation and the samples were incubated in a water bath at 37 °C for 5 h to hydrolyze potential conjugated species. After incubation, samples were acidified with 30 µL of formic acid and frozen overnight for protein precipitation. Next, samples were thawed, equilibrated to room temperature and centrifuged (2500×*g*, 10 min). The supernatant was transferred into a 400 µL vial insert and a volume of 5 µL was injected into the HPLC system.

### Sample analysis

Liquid chromatography was performed on a Thermo Scientific™ UltiMate™ 3000 system as previously outlined (Lessmann et al. 2018) with the exception of using a Kinetex^®^ C18 column (150 × 2.1 mm, 2.6 µm, 100 Å) and, in case of ACE, a Kinetex^®^ Phenyl-Hexyl (150 × 3.0 mm, 2.6 µm, 100 Å) for chromatographic separation. Mass spectrometric analysis was performed using a Thermo Scientific™ Q Exactive™ Focus Hybrid Quadrupole-Orbitrap mass spectrometry system in two separate ionization modes (heated ESI negative and positive). The mean reproducibility (triplicate injections, relative standard deviation) was <10% for all compounds. Detailed methodological information on instrument setup and LC–MS/MS conditions are outlined in the supplementary material (Tables S1–S3).

### Metabolite screening

The Qual Browser of the XCalibur™ Software (Version 4.2 SP Thermo Scientific) was used in the screening experiment to search for chromatographic peaks with the exact mass of the molecular ions of the dosed NNI and plausible metabolites thereof (Table S4a–g). The expected molecular masses of potential metabolites of the seven NNIs were mainly derived from data in the current registration dossiers (FAO/WHO 2021) and from publications on NNI metabolism in other species such as rodents (Casida 2011) and in other matrices such as water and soil (Hussain et al. 2016; Akoijam and Singh 2014). However, generally known metabolic pathways were also taken into account such as CYP oxidation of aliphatic structures including oxidative demethylation of *N*-methyl moieties. In addition, the ^13^C isotope signal patterns and, where possible, the ^35^Cl and ^37^Cl and the ^32^S und ^34^S isotopolog masses of putative metabolites were investigated. If detectable, both ionization modes were used. Excretion profiles were obtained by plotting the ^35^Cl and ^37^Cl peak areas (absolute, creatinine-adjusted, and corrected for urinary volume) for each putative metabolite against the time of sampling.

### Metabolite confirmation

Further data processing was done using the Compound Discoverer™ (CD) software version 2.0.0.303 (Thermo Scientific) as previously outlined (Lessmann et al. 2018). The software was used as an independent metabolite predictor for the screening data. All potential screening hits were evaluated but CD did not provide any additional, unexpected hits. Tentative metabolites from screening were included in a confirmation run using data-dependent MS/MS (dd-MS^2^) to obtain product ion spectra if they showed plausible ^13^C-, ^37^Cl-, and/or ^34^S-isotopolog signal patterns (± 30% of expected relative intensity), accurate signal masses (± 5 ppm) and plausible excretion kinetics in the initial screening run. The derived major mass fragments were evaluated based on the chemical formulae of the precursor ions, the postulated fragment structures thereof and after neutral losses. For further confirmation, the correlation of the peak areas in both ionization modes between ^35^ and ^37^Cl and, if possible in case of sulfur-containing metabolites, between ^32^ and ^34^S was reviewed for each tentative metabolite. For further confirmation purposes, commercially available analytical standards of putative metabolites of high relevance were purchased and retention times, isotope signal patterns, and derived product ion spectra of all analytical standards were used as additional reference. All in all, confidence levels between 1 (confirmed structure by MS, MS^2^, retention time, reference standard) and 5 (exact mass of interest only) were assigned to each of the parent NNIs or their identified metabolites in post-dose urine sample (Schymanski et al. 2014).

## Results and discussion

Currently, human biomonitoring of individuals and populations exposed to NNIs is limited due to a lack of knowledge on basic metabolic data of NNIs and specific biomarkers. This study directly addresses this need for data by identifying the most promising and specific human biomarkers of NNIs after oral dosage. For this purpose, five highly important classical neonicotinoids (ACE, IMI, CLO, THIAC and THIAM) and two major substitution products, the neonicotinoid-like compounds FLUP and SULF, have been chosen. In addition, the applied doses were based on the current EU acceptable daily intakes (ADI) and thus are environmentally relevant for the general population.

### Classical neonicotinoids

Prototypes of classical NNIs are those of the 1st and 2nd generation which, respectively, contain a 6-chloro-3-pyridinyl moiety (e.g., ACE, IMI, and THIAC) or a 2-chlorothiazole moiety (e.g., CLO and THIAM) (Fig. 1).

#### Imidacloprid

**Fig. 2:**
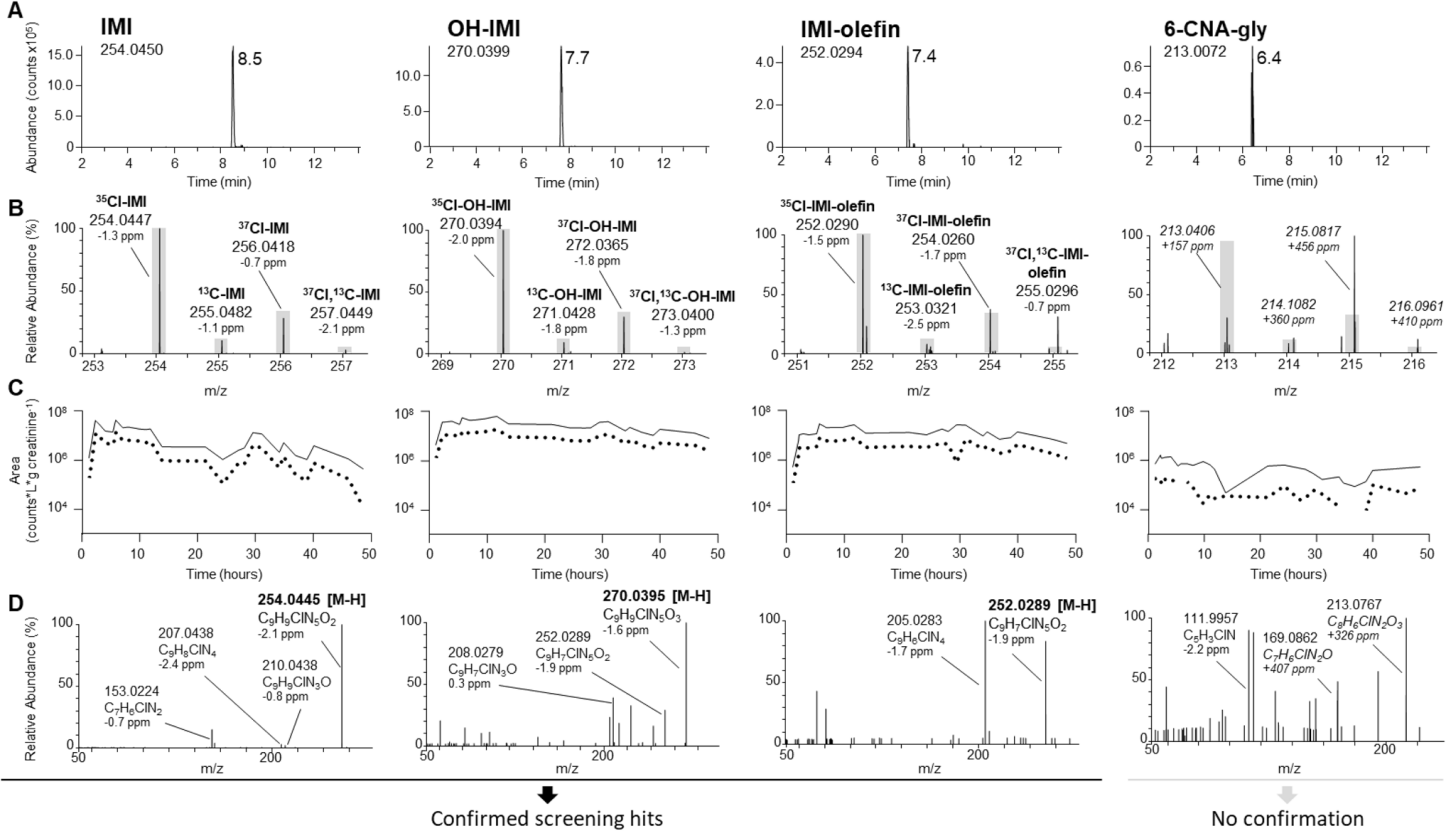
Identification of NNIs and their metabolites by LC-Q-Orbitrap-MS using imidacloprid (IMI) as an example. An initial screening (**A**–**C**) was followed by a confirmation run (**D**). **A** Extracted ion chromatograms ([M–H]^−^) of the most abundant ^35^Cl isotope of IMI (left), hydroxy-IMI (OH-IMI; middle left), IMI-olefin (middle right), and 6-chloronicotinoyl glycine (6-CNA-gly; right) 2.3 h after an oral dose of IMI. **B** Respective isotope cluster regions of [M–H]^−^ in full scan MS1 with theoretical intensities relative to the main isotope signal (grey boxes). **C** Creatinine-adjusted excretion profiles (peak area vs. time) using the ^35^Cl (bold) and the ^37^Cl isotope mass signals (dotted). **D** Product ion spectra for the ^35^Cl isotope (for fragmentation suggestions see also Figure S2a). In case of 6-CNA-gly, confirmation as an IMI metabolite failed (see main text)

The best studied substance of all NNIs so far is undoubtedly IMI, even though there are only few studies in humans. Consequently, IMI was the first NNI which we orally dosed and used as a model compound. The parent compound and three putative metabolites, hydroxylated IMI, IMI olefin and 6-CNA-glycine, could be detected in post-dose urine samples based on the extracted ion chromatograms of the molecular ions of the most abundant ^35^Cl isotopologs, the corresponding isotope cluster regions of the molecular ion, and the corresponding excretion profiles (Fig. 2). In contrast, neither IMI nor its metabolites could be identified in the pre-dose urine sample.

Confirmation analysis of the screening results by product ion spectra could validate the presence of IMI (confidence level 1), hydroxylated IMI (confidence level 3) and IMI-olefin (confidence level 1), but not 6-CNA-glycine. In example, the presence of unchanged IMI and IMI-olefin was undoubtedly verified by comparing their retention times (*t*_R_ = 8.5 and 7.4 min) and mass spectra after injecting commercially available analytical standards. In addition, unchanged IMI and both, hydroxylated IMI and IMI olefin, were detected up to 48 h post-dose in urine and a high correlation between the respective isotopic forms (^35^Cl/^37^Cl) could be found (Figure S3a). The observed prolonged excretion of unchanged IMI up to 48 h in our study is also in line with the results reported by Harada et al. (2016) who described the excretion of IMI in urine up to 96 h after oral ingestion of deuterated IMI.

In case of 6-CNA-gly, confirmation as an IMI metabolite failed due to considerable matrix interference. Even though a chromatographic peak was detected in the extracted ion chromatogram (ESI negative mode) at the accurate mass of the deprotonated 6-CNA-gly (*m*/*z* 213.0071), the excretion profile was inconclusive compared to the other metabolites (Figure S1a). Furthermore, the mass signals in the dd-MS^2^ product-ion spectra could not be attributed to the expected mass signals (Figure S2a). Finally, the expected ^35^Cl and ^37^Cl isotopolog mass signals and their ratios could not be observed, resulting in a less conclusive ^35^Cl/^37^Cl isotope correlation (Figure S3a). Overall, a confidence level of 5 could be assigned to 6-CNA-gly only.

The product ion spectra of the observed peak for hydroxylated IMI (*t*_R_ = 7.7 min), the observed neutral loss of water and the finding of IMI-olefin pointed to the formation of hydroxylated IMI at the 4- and/or 5-position of the 1-H-imidazole moiety rather than at the 6-chloronicotinoyl moiety or the methylene bridge. The retention time was also identical to those of commercially available standards of 4- and 5-OH-IMI, thus further strengthening our conclusion that IMI has been hydroxylated at the 4- and/or 5-position of the 1-H-imidazole moiety. Using an exploratory isocratic chromatographic method, we were able to separate 4- and 5-OH-IMI and could even show the formation of both metabolites in our samples. The formation of 5-OH-IMI has been previously reported in metabolism studies of rodents (Ford and Casida 2006). In addition, its formation also has been observed in humans (Taira et al. 2013; Ospina et al. 2019; Song et al. 2021; Käfferlein et al. 2021; Wrobel et al. 2020).

A dihydroxylated metabolite of IMI such as 4,5-OH-IMI could not be detected in our study albeit it has been reported previously in a qualitative profiling study (Taira et al. 2013). Similarly, desnitro-IMI (also known as IMI-guanidine) which has recently been analyzed in urine samples from China (Wang et al. 2020) could not be identified in our experiments. In addition, desnitro-IMI-olefin has been previously suggested for examination in human urine based on the fact that this metabolite has been found in lizard brain following oral exposure to IMI (Wang et al. 2018, 2020). In our study, we indeed were able to detect a chromatographic peak at the exact mass of desnitro-IMI-olefin in ESI positive ionization mode. However, this peak had the identical retention time of IMI (*t*_R_ = 8.5 min) and was also observed when analyzing a neat standard solution of 50 µg/L IMI. Accordingly, we concluded that the presence of desnitro-IMI-olefin is more likely due to in-source fragmentation involving the neutral loss of nitrous acid (as previously described in the gas-phase fragmentation of IMI-olefin (Fusetto et al. 2016)) rather than being formed as a metabolite of IMI in vivo (Figure S4).

**Fig. 3:**
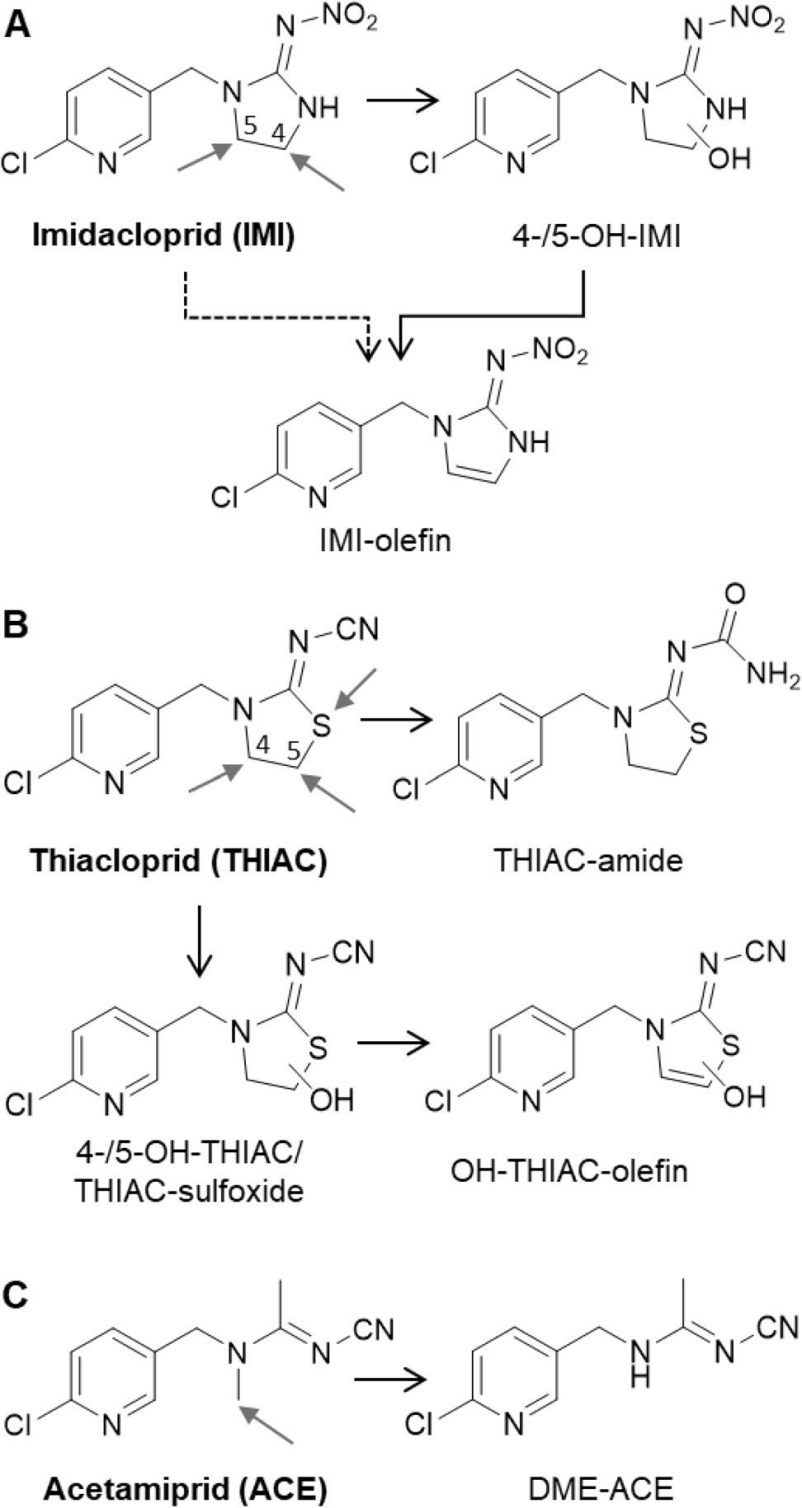
Proposed metabolism of classical NNIs of the 1st generation (containing a 6-chloro-3-pyridinyl group) in humans (**A** imidacloprid; **B** thiacloprid; **C** acetamiprid). Metabolites with a confidence level of ≤ 3 are shown; identified or most likely positions for oxidation are given (grey arrows) based on reference material, chromatographic and MS data presented in this publication and/or data from previously published manuscripts (see text)

6-CNA has been previously suggested as biomarker for NNIs which contain a 6-chloro-3-nicotinoyl moiety (Uroz et al. 2001; Kavvalakis et al. 2013; Tao et al. 2019). This suggestion is mainly based on experiments in rodents where 6-CNA has been found as a metabolite of IMI (Solecki 2001). However, we could not identify 6-CNA in post-dose urine samples by using our approach. To verify our results, we spiked urine samples with commercially available 6-CNA at different concentrations. The results show that, if present in post-dose urine samples, 6-CNA could have been detected with a limit of detection of 10 µg/L using our approach (Figure S5). Consequently, it is safe to assume that 6-CNA is excreted, if at all, in low amounts only (< 10 µg/L) after oral dosage of environmentally relevant concentrations of IMI. The proposed metabolism and urinary excretion of IMI in humans based on our experiments is summarized in Fig. 3A.

#### Thiacloprid

After oral dosage of THIAC, the parent compound and six putative metabolites could be detected in post-dose urine samples (Figure S1b, Figure S2b). The parent compound was, similar to IMI, verified by comparing the retention time (*t*_R_ = 9.9 min) and the mass spectra after injecting a commercially available analytical standard (confidence level 1). THIAC was eliminated much faster than IMI. The latter has been detected in high amounts even 48 h after dosing, while THIAC could only be found up to 24 h under identical MS screening conditions.

Next to THIAC, at least two oxidized forms of THIAC (‘THIAC + O’; neutral exact mass *m*/*z* 268.01856), a hydroxylated form of THIAC-olefin (OH-THIAC-olefin), THIAC-amide (= THIAC-urea), 6-CNA-glycine, and one metabolite of unknown structure with a molecular formula of C_11_H_15_ClN_4_O_3_S (exact mass *m*/*z* 319.0632) could be discovered (Figure S1b, Figure S2b). High correlations between the isotopic forms (^35^Cl/^37^Cl) could be found for each metabolite (Figure S3b). Neither THIAC nor any of the metabolites could be identified in the pre-dose urine sample. In contrast to IMI, an olefinic form of THIAC could not be found.

At least two ‘THIAC + O’ at retention times of *t*_R_ = 8.9 and 10.5 min (both confidence level 3) were found up to 48 and 24 h in post-dose urine samples. Based on the fragment of *m*/*z* 126 found in the product ion spectra in positive ionization mode, similar to IMI, hydroxylation might occur at the 1-H-thiazole moiety. Therefore, these two metabolites are either 4- and/or 5-OH THIAC or THIAC-sulfoxide. Due to the observed neutral loss of CO in the negative ionization mode at both retention times, the formation of the sulfoxide seems less probable. ‘THIAC + O’ might be considered as hydroxylated forms of THIAC (OH-THIAC). However, the first peak at *t*_R_ = 8.9 min appears to be a double peak thus indicating that more than one isomer with the same accurate mass is formed (Figure S7). As no sufficient chromatographic separation was achieved, it was treated as a single peak to evaluate the excretion profile of OH-THIAC.

A dehydrogenated monoxidized THIAC metabolite (‘OH-THIAC-olefin’) could be detected up to 28 h post-dose (*t*_R_ = 10.4 min, confidence level 3). In addition, the exact mass of THIAC-amide (*m*/*z* 286.02912) also gave a positive hit in the screening experiment (*t*_R_ = 8.3 min, confidence level 3) albeit its product ion spectrum was inconclusive in our experiments, possibly due to poor detection of the metabolite. Similar to OH-THIAC-olefin, THIAC-amide could be found up to 28 h post-dose in ESI negative mode, while it was only detected in few samples (*n* = 4; 2–8 h post-dose) in ESI-positive mode.

Similar to IMI, the protonated and deprotonated exact mass of 6-CNA-gly gave screening hits (*t*_R_ = 6.3 min) and could be detected for up to 24 h. However, the result could not be confirmed by dd-MS^2^ due to matrix interferences (confidence level 4). In addition, the pre-cursor prior phase-II conjugation (6-CNA) could not be identified in any of the post-dose urine samples above our limit of detection (10 µg/L, Figure S5).

Last but not least, one metabolite named by its molecular formula C_11_H_15_ClN_4_O_3_S associated to the accurate mass of *m*/*z* 319.0632 (*t*_R_ = 6.8 min) was detected in ESI-positive ionization mode (confidence level 4). Based on the obtained dd-MS^2^ spectra, calculated neutral losses affirm the plausibility of the proposed molecular formula, but no potential structure could be concluded from the obtained data. In summary, the proposed metabolism of THIAC based on our experiments is shown in Fig. 3B.

#### Acetamiprid

The parent compound and two putative metabolites, *N*-desmethyl-ACE (DME-ACE) and 6-CNA-gly, were detected in both ionization modes after oral dosage of ACE. The initial extracted ion chromatogram of the exact mass of ACE (*m*/*z* 223.07450, ESI-positive ionization, [M + H]^+^) showed strong mass interferences visible already for the pre-dose sample and resulted in an overlay with the chromatographic peak of ACE in the post-dose samples. After modification of the analytical method (Table S2, Table S3), the interfering matrix could be chromatographically separated. Unchanged ACE was detected up to 24 h post-dose in urine (Figure S1c) at the retention time of the analytical standard (*t*_R_ = 7.9 min) and with comparable mass spectra (Figure S2c) (confidence level 1).

The formation of a specific putative metabolite, DME-ACE (*t*_R_ = 7.3 min) has been, similar to ACE, unequivocally confirmed at the retention time of the analytical standard and by its dd-MS^2^ spectra (confidence level 1). DME-ACE appeared in the first urine sample after dosing (3.9 h) and remained at more or less constant levels up to 48 h post-dose (Figures S1c and S2c). Again, for both ACE and DME-ACE, high correlations between the isotopic forms (^35^Cl/^37^Cl) could be found (Figure S3c). Our results are in line with Harada and co-workers who also reported DME-ACE as a major metabolite after controlled dosage of humans with deuterated ACE (Harada et al. 2016).

The observed excretion kinetics of ACE and DME-ACE suggest that ACE is rapidly converted into DME-ACE before excretion and proof a high capacity of oxidative *N*-demethylation in humans. Interestingly, we already detected DME-ACE in the pre-dose urine sample at low levels, suggesting background exposures to ACE in the general population. This finding is in line with previous studies where DME-ACE has been analyzed in urine samples of the general population (Song et al. 2021; Baker et al. 2019) or after accidental intake of NNI-containing insecticide formulations (Taira et al. 2013).

The putative 6-CNA-gly (*t*_R_ = 6.2 min) could be detected in both ionization modes and a high isotopic ^35^Cl/^37^Cl correlation could be found. Yet, it still could not be confirmed through dd-MS^2^ (confidence level 4). All in all, our results suggest that ACE is almost exclusively metabolized to DME-ACE in humans (Fig. 3C).

#### Thiamethoxam

For THIAM, a NNI of the 2nd generation containing a 2-chlorothiazole moiety, the parent compound was detected up to 48 h in post-dose urine samples (Figure S1d and S2d), at the retention time of the analytical standard (*t*_R_ = 7.4 min), and with comparable mass spectra (Figure S2d) (confidence level 1). Excretion was slow and, similar to all other NNIs, not finished after 48 h.

THIAM has been reported to be metabolized to CLO in rodents (Casida 2011) after cleavage of the 1,3,5-oxadiazine moiety, a metabolic step which we also observed here in humans. CLO was unequivocally confirmed by analytical standard material (*t*_R_ = 8.2 min) and mass spectra (confidence level 1). Similar to THIAM, the excretion of CLO was slow and considerable amounts were still detected after 48 h. As observed for the metabolism of ACE, CLO was further metabolized by oxidative demethylation to *N*-desmethyl-CLO (DME-CLO; *t*_R_ = 7.7 min) and this metabolite could be also unequivocally confirmed in the urine samples by its retention time and mass spectra compared to an analytical standard (confidence level 1) (Figure S2d). Levels of DME-CLO started to increase the first 12–24 h and then DME-CLO was more or less excreted at constantly high levels up to 48 h and possibly beyond (Figure S1d). The formation of DME-CLO from either THIAM or directly from CLO is also in line with Taira and co-workers who previously suggested its formation when analyzing urine samples of persons after acute intoxication of NNI-containing insecticide formulations (Taira et al. 2013).

We also screened for the exact mass of *N*-desmethyl-THIAM (DME-THIAM; 8.9 min). However, this metabolite was detectable in few samples only (in ESI positive mode) and the elimination kinetics could not be studied. In addition, the isotopic pattern drawn from the full scan MS^1^ was inconclusive and no product ion spectra could be obtained possibly due to its poor detection. Instead, a putative cysteinyl analogue of THIAM (cys-THIAM, *t*_R_ = 3.6 min) could be detected which is most likely derived by direct GSH conjugation in the presence of GSH transferases and subsequent degradation to its cysteine-*S*-conjugate in the liver. This newly identified metabolite was observed between 1.4 and 48 h post-dose. However, its main excretion occurred in the first 24 h. Interestingly, a similar metabolite could not be observed in NNIs of the 1st generation, i.e., by substitution of chlorine in the 6-chloronicotinoyl moiety. Overall, high correlations were observed between the ^35^Cl/^37^Cl isotopes in case of THIAM, CLO and DME-CLO and ^34^S/^36^S isotopes of cys-THIAM (Figure S3d).

#### Clothianidin

Based on the aforementioned formation of CLO after oral dosage of THIAM, there is considerable overlap between the metabolism of THIAM and CLO. After oral dosage of CLO, both the parent compound and DME-CLO were the main compounds excreted in urine and both compounds could be confirmed at the retention time of the respective analytical standards (*t*_R_ = 8.2 and 7.7 min) and with comparable mass spectra in both ionization modes (confidence level 1) (Figure S2e). In contrast to THIAM, where CLO is slowly formed during the first 24 h, CLO and DME-CLO excretion after direct oral dosage of CLO were constantly excreted at high and more or less constant levels from the start and up to 48 h. Similar to DME-ACE, DME-CLO has been previously described after accidental intake of NNI-containing insecticide formulations (Taira et al. 2013).

As described for THIAM, we also observed the formation of a cysteinyl analogue of CLO (cys-CLO, *t*_R_ = 4.9 min) (confidence level 3). In contrast to THIAM, however, we additionally observed the formation of CLO-urea (*t*_R_ = 6.7 min) (confidence level 2b). Both metabolites, cys-CLO and CLO-urea, were excreted in substantial amounts up to 42 h after oral dosage.

Similar to 6-CNA, 2-CTA has been postulated as a metabolite of NNIs containing a 2-chlorothiazole group (Nomura et al. 2013). However, 2-CTA could not be detected at levels above 10 µg/L (Figure S8) thus must be considered a metabolite of minor relevance in humans.

The presence of CLO and DME-CLO in urine can either indicate exposure to THIAM and/or CLO. Therefore, CLO and DME-CLO are, similar to 6-CNA and 2-CTA, not specific for a single NNI. However, cys-THIAM and cys-CLO are highly specific and both metabolites were excreted in relevant amounts after exposure to THIAM and CLO. Furthermore, CLO-urea was not found after oral dosage of THIAM and might also be specific for CLO. However, the lack of CLO-urea after dosing THIAM may be due to the lower administered dose of THIAM compared to CLO. Overall, to distinguish between THIAM and CLO exposure, it is advisable to analyze the parent compounds together with the metabolites for biomonitoring purposes. Then, a lack of THIAM clearly identifies CLO as source of exposure. However, the presence of THIAM may not exclude co-exposures to CLO.

**Fig. 4:**
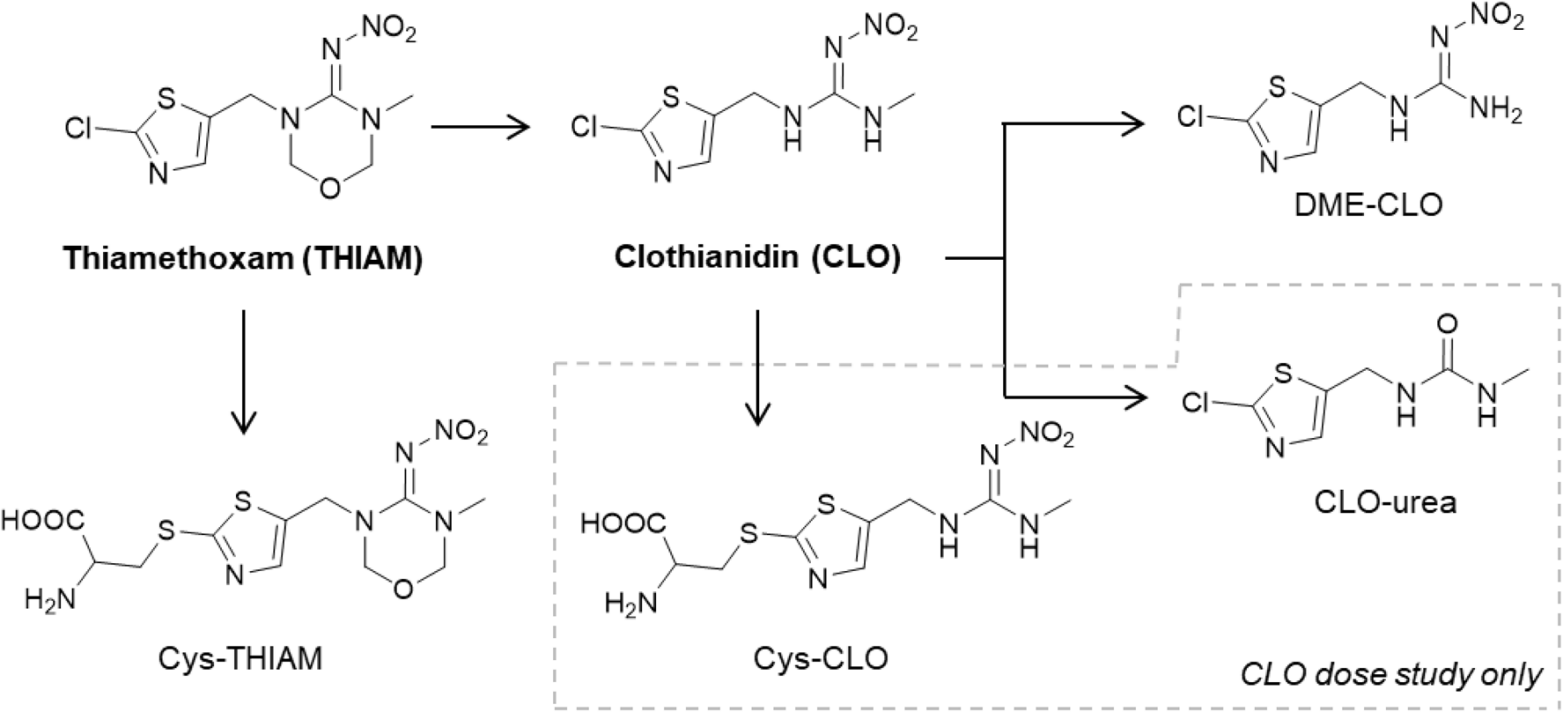
Proposed metabolism of classical NNIs of the 2nd generation (containing a 2-chlorothiazole moiety) in humans including thiamethoxam (THIAM) and clothianidin (CLO). Metabolites with a confidence level of ≤ 3 are shown

We found high correlations for DME-CLO between positive and negative ionization modes after oral dosage of THIAM (Figure S3d), while, unexpectedly, a correlation after oral dosage of CLO was absent (Figure S3e). Based on this result, one also would have assumed that a high correlation between ^35^Cl/^37^Cl isotopes for DME-CLO is observed only after dosage of THIAM. However, the ^35^Cl/^37^Cl isotope correlations for DME-CLO were high after both THIAM and CLO dosages. This phenomenon might be explained by unknown matrix effects which influences ionization. In summary, based on a substantial overlap, the proposed metabolism of THIAM and CLO in humans is shown in Fig. 4.

### Neonicotinoid-like compounds

#### Flupyradifurone

Unchanged FLUP was found up to 48 h post-dose (Figure S1f) at the retention time of the analytical standard (*t*_R_ = 9.5 min) and with comparable mass spectra (Figure S2f) in both ionization modes (confidence level 1). Three isomers of mono-oxidized FLUP were detected (*t*_R_ = 9.2, 9.3 and 9.8 min) up to 48 h post-dose of which the first eluting peak was detectable in positive ionization mode only (confidence level 3) (Figure S9). Also, two metabolites formed by *N*-dealkylation could be detected (confidence level 2b). FLUP-des-difluoroethyl (DFE-FLUP; *t*_R_ = 7.6 min) could be identified in both ionization modes and confirmed through its product ion spectra by dd-MS^2^. In contrast, des-chloropyridinyl-FLUP (DCP-FLUP; *t*_R_ = 5.1 min) could be detected and confirmed through dd-MS^2^ in negative mode only*.* Each of the peaks of both metabolites was observed at different retention times from that of the parent substance (FLUP). Therefore, in-source fragmentation and formation of DFE-FLUP and DCP-FLUP directly from FLUP rather than their metabolic formation could be ruled out (Figure S10).

The exact positions of all hydroxyl groups in mono-oxidized FLUP cannot be assigned definitively. The observation of three peaks (a double peak at 9.2 and 9.3 and a single peak at 9.8 min) indicates that at least three different hydroxylated isomers are formed (Figure S2f). The obtained ddMS^2^ spectrum of the isomer which elutes at 9.2 min with a specific mass fragment of *m*/*z* 225 (Figure S2f) can be only explained by hydroxylated FLUP at the difluoroethyl group. This position is also supported by the subsequent formation of DFE-FLUP which, similar to oxidative *N*-demethylation, most likely occurs via oxidative *N*-desdifluoroethylation. The product ion spectra of the two remaining isomers at *t*_R_ = 9.2 and 9.8 min indicate hydroxylation at the 2(5H)-furanone (2-butenolide) moiety.

**Fig. 5:**
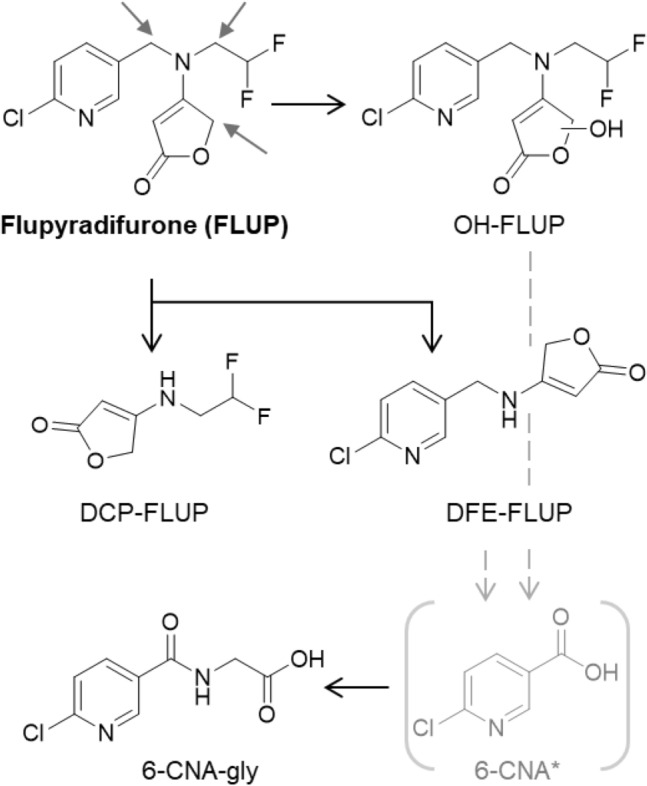
Proposed metabolism of flupyradifurone (FLUP) in humans. Metabolites with a confidence level of ≤ 3 are shown; identified or most likely positions for oxidation are given (grey arrows) based on chromatographic and MS data presented in this publication; the pre-curser of 6-CNA-gly, 6-CNA (gray), could not be detected (see text)

In contrast to the classical neonicotinoids of the 1st generation which, similar to FLUP, contain a 6-chloronicotinoyl moiety (IMI, THIAC, ACE), 6-CNA-gly (*t*_R_ = 6.3 min) could be clearly confirmed by its elimination kinetics (Figure S1f) and product ion spectrum (Figure S2f) after oral dosage of FLUP (confidence level 2b). However, its pre-cursor 6-CNA, could not be detected at levels ≥ 10 µg/L similar to the metabolism of IMI. The proposed metabolism of FLUP in humans is shown in Fig. 5.

#### Sulfoxaflor

Unchanged SULF was detected up to 48 h post-dose (Figures S1g) at the retention time of the analytical standard (*t*_R_ = 9.9/10.1 min) and with comparable mass spectra (Figure S2g) for both ionization modes. The chromatographic peak appeared as a double peak which could be explained by the presence of two chiral centers in the molecule and therefore four stereoisomers of which two diastereomeric forms can be chromatographically separated by our method.

Previously, up to nine different metabolites have been reported in liver homogenates, and bile and urine samples of rats orally dosed with SULF (Xu et al. 2020). Seven out of the nine metabolites have been oxidized at the 6-(trifluoromethyl)-nicotinoyl moiety. The four metabolites which have been detected in urine include 1- and/or 2-hydroxy sulfoxaflor, glucuronated derivative(s) thereof, and S-oxidized SULF. None of the nine metabolites were detected in our study. In addition, a thorough search for other metabolites including demethylated sulfoxaflor or degradation products after cleavage of the CH-bridge such as 1-(6-(trifluoromethyl)pyridin-3-yl)ethanol, 5-acetyl-2-(trifluoromethyl)pyridine, 6-trifluoromethylnicotinic acid or its glycine conjugate did not reveal hits in our study. The differences between our results and those derived by Xu and co-workers may be due to a combination of, among others, interspecies differences (rats vs. humans), differences in the applied absolute dose (human: 3 mg; rats: ~ 20 mg), duration (human: single exposure; rat: repeated exposures at 125 mg/kg/day for 6 weeks), and different analytical sensitivities.

## Concluding remarks

In this study, we successfully applied a screening approach in urine samples from low oral dose studies using LC–HRMS to investigate the human metabolism and urinary excretion of seven important NNIs. In addition to the parent compounds (*n* = 7), we detected 24 metabolites. A confidence level of 1 or 2b (confirmed or probable structure) could be assigned to eleven metabolites and a confidence level of 3 (tentative metabolite candidates) to ten metabolites (Table 2). Using this approach, we were able to confirm previously reported key metabolites in humans such as DME-ACE, DME-CLO, OH-IMI, IMI-olefin, CLO-urea, and THIAC-amide (Taira et al. 2013; Harada et al. 2016), question the relevance of certain metabolites which have been previously assumed of being key metabolites in humans such as 6-CNA or 2-CTA, and ultimately identify new key metabolites in humans such as cys-CLO, OH-FLUP, DFE-FLUP, DCP-FLUP, OH-THIAC, OH-THIAC-olefin, and cys-THIAM. In addition, we were able to show that both, 4- and 5-OH-IMI, are formed after oral dosage of IMI rather than just 5-OH-IMI only as previously reported (Song et al. 2021; Ospina et al. 2019).

**Table 2:** Identified and confirmed metabolites (*n* = 24) of seven dosed neonicotinoids and neonicotinoid-like compounds (NNIs) including name, molecular formula, retention time (*t*_R_), and confidence level of their identification based on Schymanski et al. (2014)

	Dosed substance	Identified substances and metabolites in urine			
		Name	Molecular formula	*t*_R_ (min)	Confidence level
Classical neonicotinoids	**IMI**	**IMI**	**C**_**9**_**H**_**10**_**ClN**_**5**_**O**_**2**_	**8.5**	**1**
		4-OH-IMI	C_9_H_10_ClN_5_O_3_	7.7	1^a^
		5-OH-IMI	C_9_H_10_ClN_5_O_3_	7.7	1^a^
		IMI-olefin	C_9_H_8_ClN_5_O_2_	7.4	1
	**THIAC**	**THIAC**	**C**_**10**_**H**_**9**_**ClN**_**4**_**S**	**9.9**	**1**
		OH-THIAC-olefin	C_10_H_7_ClN_4_OS	10.4	3
		OH-THIAC/THIAC-sulfoxide (3)	C_10_H_9_ClN_4_OS	8.8/8.9 + 10.5	3
		THIAC-amide	C_10_H_11_ClN_4_OS	8.3	3
		6-CNA-gly	C_8_H_7_ClN_2_O_3_	6.3	4
		‘C_11_H_15_ClN_4_O_3_S’	C_11_H_15_ClN_4_O_3_S	6.8	4
	**ACE**	**ACE**	**C**_**10**_**H**_**11**_**ClN**_**4**_	**7.9**	**1**
		DME-ACE	C_9_H_9_ClN_4_	7.3	1
		6-CNA-gly	C_8_H_7_ClN_2_O_3_	6.2	4
	**THIAM**	**THIAM**	**C**_**8**_**H**_**10**_**ClN**_**5**_**O**_**3**_**S**	**7.4**	**1**
		CLO	C_6_H_8_ClN_5_O_2_S	8.2	1
		DME-CLO	C_5_H_6_ClN_5_O_2_S	7.7	1
		Cys-THIAM	C_11_H_16_N_6_O_5_S_2_	3.6	3
	**CLO**	**CLO**	**C**_**6**_**H**_**8**_**ClN**_**5**_**O**_**2**_**S**	**8.2**	**1**
		DME-CLO	C_5_H_6_ClN_5_O_2_S	7.7	1
		CLO-urea	C_6_H_8_ClN_3_OS	6.7	2b
		Cys-CLO	C_9_H_14_N_6_O_4_S_2_	4.9	3
NNI-like compounds	**FLUP**	**FLUP**	**C**_**12**_**H**_**11**_**ClF**_**2**_**N**_**2**_**O**_**3**_	**9.5**	**1**
		OH-FLUP (3)	C_12_H_11_ClF_2_N_2_O_4_	9.2, 9.3 + 9.8	3
		DFE-FLUP	C_10_H_9_ClN_2_O_2_	7.6	2b
		6-CNA-gly	C_8_H_7_ClN_2_O_3_	6.3	2b
		DCP-FLUP	C_6_H_7_F_2_NO_2_	5.1	2b
	**SULF**	**SULF**	**C**_**10**_**H**_**10**_**F**_**3**_**N**_**3**_**OS**	**9.9/10.1**	**1**

The number of detected isomers is given in parentheses behind the corresponding name. Confidence level (and required information). 1: confirmed structure (MS, MS^2^, retention time, reference standard); 2a: probable structure (MS, MS^2^, library MS^2^); 2b: probable structure (MS, MS^2^, experimental data); 3: tentative candidate (MS, MS^2^, experimental data); 4: unequivocal molecular formula (MS isotope/adduct); 5: exact mass of interest (MS)

^a^A separate isocratic method showed the formation of both, 4- and 5-OH-IMI (see text)

For IMI, THIAC and FLUP, oxidation at the 1-H-imidazole, 1-H-thiazole and 2(5H)-furanone moiety, respectively, has been shown a major metabolic step. In contrast, oxidative *N*-demethylation turned out to be the initial metabolic step for ACE and CLO. Oxidative *N*-demethylation must also be considered relevant for THIAM, even though DME-THIAM could not be found consistently in all urine samples and only after positive ionization. In addition, in case of FLUP, oxidative *N*-dealkylation in terms of *N*-desdifluoroethylation is of at least equal importance compared to oxidation at the 2(5H)-furanone moiety. Generally, no oxidation in the heteroaromatic ring system (nicotinoyl group) or thiazole moiety could be detected in our study. However, as in any (non-quantitative) screening approach, the presence of putative metabolites which have not been detected in our approach cannot be excluded due to a potential lack of sensitivity for certain metabolites and based on the targeted nature of our suspect screening approach. By using a glucuronidase/sulfatase treatment of the urine samples we tried to balance our sample preparation with regard to the number of potentially identified metabolites in our screening approach and the sensitivity of the method. However, we were aware that this type of sample treatment discriminates the respective phase-II metabolites. Conversely, the enzymatic hydrolysis increased the chance of detecting hydroxylated species which are important phase-I metabolites and biomarkers to assess human exposures to xenobiotics. Therefore, using an enzymatic hydrolysis was a calculated risk that was worthwhile, especially because phase-II metabolites generally elute early from the column, often coelute with large amount of matrix or even during the dead time of the column and thus ultimately result in decreased sensitivities or even the complete loss of these metabolites. Alternatively, using an SPE technique was no option due to the risk of completely discriminating certain metabolites or groups of metabolites in our screening approach. A further adaptation of our technique, e.g., by employing a "dilute and shoot" strategy, was also not necessary because our sample preparation technique provided a significant number of screening hits with sufficient sensitivities.

Because this study was conducted as a qualitative screening to identify specific metabolites of NNIs in humans, the actual sensitivity for each possible metabolite remains unknown. In addition, the detector response in ESI–MS can be significantly affected even by minor changes in the chemical structure. Consequently, quantitatively meaningful metabolites might have been missed due to poor sensitivities of the method for these metabolites. Interestingly, we could not readily detect 6-CNA and 2-CTA in our screening approach after oral dosage of NNIs although these two metabolites have been promoted as promising biomarkers of NNI exposures (Tao et al. 2019; Gries et al. 2018; Nomura et al. 2013). Therefore, we wanted to know whether this particular lack of detection was due to a lack in analytical sensitivity or due to a low formation of these compounds (if any). We consequently checked the actual sensitivities for 6-CNA and 2-CTA (10 µg/L) using commercially available standard material and thus were able to show that 6-CNA and 2-CTA are of minor relevance for assessing exposures to NNIs.

Although NNIs and the majority of the identified metabolites appeared in urine very rapidly, they were well detectable up to 48 h (our observation time) thus indicating a prolonged excretion in urine after oral dosage (Figures S1a–S1g). In addition, we also observed differences in the excretion of the varying NNIs, i.e., showing slower elimination of the novel fluorinated compounds such as FLUP and SULF which have been introduced as less critical alternatives to the classical NNIs. The prolonged excretion cannot be considered a methodological artifact (e.g., detector saturation) because, compared to other MS techniques, the ion population in the mass analyzer of Orbitrap instruments can be controlled (Automatic Gain Control, AGC) and thus MS-orbitrap measurements are less susceptible to detector saturation (Kalli et al. 2013).

The study has been carried out in a single male volunteer. Having at least three or more volunteers of both sexes would have most likely substantiated our findings. Interestingly, significant intraspecies differences in experimental studies with controlled exposures in small sets of volunteers could not be observed for other xenobiotics (Modick et al. 2016; Koch et al. 2014; Gotthardt et al. 2021; Moos et al. 2016). The lack of such differences is most likely due to the generally limited number of studied volunteers in such studies although small but significant intraspecies differences, if present, may become evident when analyzing larger populations.

Although ecotoxicological aspects of NNIs have always been the focus of public interest, potential chronic effects of NNIs in humans also needs to be considered on the long term. At present, a myriad of reports on the effects of NNIs in pollinators is ultimately contrasted by only few studies on human exposures and potential chronic effects of NNIs in humans, even though NNIs are extensively used in agriculture and households and, consequently, have become ubiquitous in our environment. Particularly, actual data on human exposure remains scarce (Cimino et al. 2017), possibly due to a lack of sensitive and specific analytical methods for the determination of NNIs and their metabolites in biological materials of humans such as urine. It is also unclear whether the proven ecotoxicological effects of neonicotinoids on pollinators or suspected chronic effects in humans are solely associated with the parent compounds or, at least in part, can also be attributed to certain metabolites. For example, IMI olefin has been also shown to be a highly potent insecticide rather than IMI alone (Dai et al. 2006).

So far, with the exception of 5-OH-IMI and DME-ACE (Ospina et al. 2019; Baker et al. 2019; Wrobel et al. 2020; Song et al. 2021), the majority of published methods proposed the use of unchanged NNIs in urine as biomarkers of exposure (Osaka et al. 2016; Ichikawa et al. 2019; Harada et al. 2016). For the first time, the present study identified promising and, most importantly, specific metabolites of NNIs in humans by a targeted approach, i.e. after controlled oral exposures. In addition, these metabolites were formed under realistic exposure scenarios, i.e., at exposures at the acceptable daily intake. Consequently, our results may serve as a basis for the future development of biomonitoring methods for exposure assessment in humans and based on specific metabolites in urine.

## Supplementary Information

Below is the link to the electronic supplementary material.

Supplementary file1 (PDF 1895 KB)
