# Supplementary material for: Human metabolism and urinary excretion of seven neonicotinoids and neonicotinoid-like compounds after controlled oral dosages
Source: Arch Toxicol. 2021 Oct 13;96(1):121–34. doi: 10.1007/s00204-021-03159-0 (PMC8748328; doi:10.1007/s00204-021-03159-0)

Electronic Supplementary Material (Wrobel et al. 2021)

Human metabolism and urinary excretion of seven neonicotinoids and neonicotinoid-like compounds after single oral dosages

Sonja A. Wrobel[1], Daniel Bury[1], Heiko Hayen[2], Holger M. Koch[1], Thomas Brüning[1], Heiko U. Käfferlein[1,*]

[1]Institute for Prevention and Occupational Medicine of the German Social Accident Insurance - Institute of the Ruhr-Universität Bochum (IPA), Bürkle-de-la-Camp-Platz 1, 44789 Bochum, Germany

[2]Institute of Inorganic and Analytical Chemistry, University of Münster, Correnssstraße 30, 48149 Münster, Germany

* Corresponding author: Heiko U. Käfferlein (kaefferlein@ipa-dguv.de)

Supplementary Tables

		Page(s)
Table S1	Solvent gradient for the experiments with CLO, FLUP, IMI, THIAC, THIAM, and SULF	2
Table S2	Solvent gradient for the experiment with ACE	2
Table S3	Instrument settings used for metabolite screening	2
Table S4a-4g	Exact masses of putative metabolites that were screened for IMI, THIAC, ACE, CLO, THIAM, FLUP, and SULF	3-7

Supplementary Figures

		Page(s)
Figure S1a-1g	Urinary excretion kinetics for IMI, THIAC, ACE, CLO, THIAM, FLUP, SULF and their metabolites	8-15
Figure S2a-2g	Confirmation analyses of IMI, THIAC, ACE, CLO, THIAM, FLUP, SULF and its metabolites	16-30
Figure S3a-3g	Pearson correlations (ionization modes, Cl-isotopologs) for IMI, THIAC, ACE, CLO, THIAM, FLUP, SULF and its metabolites	31-38
Figure S4	Artefactual formation of desnitro-IMI-olefin due to MS-in-source fragmentation	39
Figure S5	Extracted ion chromatograms and dd MS^2 spectra of 6-CNA in water and urine	39
Figure S6	Molecular structures of isomers of OH-THIAC, THIAC-amide-olefin, and THIAC-sulfoxide	40
Figure S7	Extracted ion chromatogram of hydroxylated THIAC isomers	40
Figure S8	Extracted ion chromatograms and dd MS^2 spectra of 2-CTA in water and urine	41
Figure S9	Extracted ion chromatogram of hydroxylated FLUP isomers	41
Figure S10	Extracted ion chromatogram of FLUP, DFE-FLUP, and DCP-FLUP	42

Table S1: Solvent gradient for the Kinetex® C18 column (150x2.1 mm, 2.6 µm) with a corresponding SecurityGuard ULTRA Cartridge (Phenomenex, Aschaffenburg, Germany) for the experiments with CLO, FLU, IMI, THIAC, THIAM, and SULF. Solvents A: water and B: acetonitrile, both containing 0.05% of acetic acid. The flow rate was set to 0.2 mL/min and column temperature was set to 25 °C.

Time [min]	A [%]	B [%]
0	95	5
1	95	5
17	5	95
22	5	95
22,1	95	5
30	95	5

Table S2: Solvent gradient for the Kinetex® Phenyl-Hexyl column (150x3.0mm, 2.6 µm) with a corresponding SecurityGuard ULTRA Cartridge (Phenomenex, Aschaffenburg, Germany) for the experiment with ACE. Solvents A: water and B: acetonitrile, both containing 0.05% of acetic acid. The flow rate was set to 0.3 mL/min and column temperature was set to 25 °C.

Time [min]	A [%]	B [%]
0	95	5
1	95	5
11	5	95
15	5	95
15,5	95	5
20	95	5

Table S3: Instrument settings used for metabolite screening in both, heated negative and heated positive ionization mode. (*These settings were applied for screening for ACE metabolites.)

**Tune parameters**		
Ionization mode	**Positive**	**Negative**
Sheath gas flow rate	45	
Aux gas flow rate	10	
Sweep gas flow rate	2	
Spray voltage	3.5 kV	2.5 kV
Capillary temperature	250 °C	
Aux gas heater temperature	400 °C	
S-lens RF level	50	
**Full MS** (experiment 1 and 2)		
In-source CID	-	
Resolution	70,000	
Scan Range	90-500 m/z	
AGC target	1e6	
Maximum IT	auto	
Microscans	1	
Spectrum data type	Profile	
**dd-MS^2 Confirmation** (only experiment 2)		
Resolution	70,000	
Isolation window	1.0 u	
CE	10 to 25 eV depending on compound	
Fixed first mass	-	
Default charge state	1	
AGC target	5e4	
Maximum IT	30ms (*auto)	
Loop count	3	
Minimum AGC target	1.5e2 (*8.0e3)	
Intensity threshold	5e3 (*auto)	
Apex trigger	1 to 9 s	
Dynamic exclusion	3.0s (*auto)	
Spectrum data type	Profile	

Table S4a: Exact masses of putative metabolites that were screened for after dosing imidacloprid (IMI) with given molecular formula in uncharged form. Based on the exact masses, *m/z* ratios for $[M+H]^+$ and $[M\text{-}H]^-$ are given. Masses that were added for confirmation by dd-MS^2 are marked with "x".

**Ionization mode**				**Formula [M]**	**Name**
**Positive $[M+H]^+$**		**Negative $[M\text{-}H]^-$**			
140.03422		138.01967		$C_6H_5NO_3$	
143.03705		141.02250		$C_6H_7ClN_2$	
145.03410		143.01955		$C_6H_7{}^{37}Cl\ N_2$	
158.00033		155.98578		$C_6H_4ClNO_2$	^{35}Cl-6CNA
159.99738		157.98283		$C_6H_4{}^{37}ClNO_2$	^{37}Cl-6CNA
167.02502		166.01775		$C_7H_7ClN_3$	
169.02207		168.01480		$C_7H_7{}^{37}ClN_3$	
170.02703		168.01247		$C_6H_7NO_2S$	
186.07925		184.0647		$C_8H_{12}ClN_3$	
188.07630		186.06175		$C_8H_{12}{}^{37}ClN_3$	
209.05885		207.04430		$C_9H_{10}ClN_4$	^{35}Cl-desnitro-IMI-olefin
211.05590		209.04135		$C_9H_{10}{}^{37}ClN_4$	^{37}Cl-desnitro-IMI-olefin
211.07450		209.05995		$C_9H_{10}ClN_4$	^{35}Cl-IMI-guanidine
212.05852		210.04396		$C_9H_{10}ClN_3O$	^{35}Cl-IMI-urea
213.07155		211.05700		$C_9H_{10}{}^{37}ClN_4$	^{37}Cl-IMI-guanidine
214.05557		212.04101		$C_9H_{10}{}^{37}ClN_3O$	^{37}Cl-IMI-urea
215.02180	x	213.00724	x	$C_8H_7ClN_2O_3$	^{35}Cl-6CNA-glycine
217.01885	x	215.00429	x	$C_8H_7{}^{37}ClN_2O_3$	^{37}Cl-6CNA-glycine
226.08540		224.07085		$C_9H_{12}ClN_5$	
227.04849		225.03394		$C_9H_{10}N_2O_3S$	
227.04849		225.03394		$C_9H_{11}N_2O_3S$	
228.08245		226.06790		$C_9H_{12}{}^{37}ClN_5$	
240.06466		238.05011		$C_9H_{10}ClN_5O$	
242.06171		240.04716		$C_9H_{10}{}^{37}ClN_5O$	
254.04393	x	252.02938	x	$C_9H_8ClN_5O_2$	^{35}Cl-IMI-olefin
256.04098	x	254.02643	x	$C_9H_8{}^{37}ClN_5O_2$	^{37}Cl-IMI-olefin
256.05958	x	254.04503	x	$C_9H_{10}ClN_5O_2$	^{35}Cl-IMI
258.05663	x	256.04208	x	$C_9H_{10}{}^{37}ClN_5O_2$	^{37}Cl-IMI
272.05449	x	270.03994	x	$C_9H_{10}ClN_5O_3$	^{35}Cl-OH-IMI
274.05154	x	272.03699	x	$C_9H_{10}{}^{37}ClN_5O_3$	^{37}Cl-OH-IMI
275.02517		273.01061		$C_{10}H_{12}ClN_2O_3S$	
277.02222		275.00766		$C_{10}H_{12}{}^{37}ClN_2O_3S$	
288.04941		286.03486		$C_9H_{10}ClN_5O_4$	^{35}Cl-Di-OH-IMI
290.04646		288.03190		$C_9H_{10}{}^{37}ClN_5O_4$	^{37}Cl-Di-OH-IMI

Table S4b: Exact masses of putative metabolites that were screened for after dosing thiacloprid (THIAC) with given molecular formula in uncharged form. Based on the exact masses, *m/z* ratios for $[M+H]^+$ and $[M-H]^-$ are given. Masses that were added for confirmation by dd-MS^2 are marked with "x".

**Ionization mode**				**Formula [M]**	**Name**
**Positive $[M+H]^+$**		**Negative [M-H]$^-$**			
142.00696		139.99241		$C_4H_3N_3OS$	
158.00033		155.98578		$C_6H_4ClNO_2$	^{35}Cl-6CNA
159.99738		157.98283		$C_6H_4{}^{37}ClNO_2$	^{37}Cl-6CNA
210.0541		208.03955		$C_8H_8ClN_5$	
212.05115		210.03660		$C_8H_8{}^{37}ClN_5$	
214.01685		212.0023		$C_8H_7NO_4S$	
215.0218	x	213.00724	x	$C_8H_7ClN_2O_3$	^{35}Cl-6CNA-glycine
217.01885	x	215.00429	x	$C_8H_7{}^{37}ClN_2O_3$	^{37}Cl-6CNA-glycine
227.04849		225.03394		$C_9H_{10}N_2O_3S$	
237.03921		235.03921		$C_{10}H_9ClN_4O$	
239.05081		237.05377		$C_{10}H_9{}^{37}ClN_4O$	
251.01527		249.00072		$C_{10}H_7ClN_4S$	^{35}Cl-THIAC-olefin
253.01232		250.99777		$C_{10}H_7{}^{37}ClN_4S$	^{37}Cl-THIAC-olefin
253.03092	x	251.01637		$C_{10}H_9ClN_4S$	^{35}Cl-THIAC
255.02797	x	253.01342		$C_{10}H_9{}^{37}ClN_4S$	^{37}Cl-THIAC
267.01019	x	264.99563	x	$C_{10}H_7ClN_4OS$	^{35}Cl-OH-THIAC-olefin
269.00724	x	266.99268	x	$C_{10}H_7{}^{37}ClN_4OS$	^{37}Cl-OH-THIAC-olefin
269.02584	x	267.01128	x	$C_{10}H_9ClN_4OS$	^{35}Cl-OH-THIAC
271.02289	x	269.00833	x	$C_{10}H_9{}^{37}ClN_4OS$	^{37}Cl-OH-THIAC
271.04149	x	269.02693	x	$C_{10}H_{11}ClN_4OS$	^{35}Cl-THIAC-amide
273.03854	x	271.02398	x	$C_{10}H_{11}{}^{37}ClN_4OS$	^{37}Cl-THIAC-amide
283.04149		281.02693		$C_{11}H_{11}ClN_4OS$	
285.02075		283.00620	x	$C_{10}H_9ClN_4O_2S$	^{35}Cl-Di-OH-THIAC
285.03854		283.02398		$C_{11}H_{11}{}^{37}ClN_4OS$	
287.0178		285.00328	x	$C_{10}H_9{}^{37}ClN_4O_2S$	^{37}Cl-Di-OH-THIAC
301.05205	x	299.03750	x	$C_{11}H_{13}ClN_4O_2S$	
303.0491	x	301.03455	x	$C_{11}H_{13}{}^{37}ClN_4O_2S$	
319.06262	x	317.04806		$C_{11}H_{15}ClN_4O_3S$	C11H15ClN4O3S
321.05967	x	319.04511		$C_{11}H_{15}{}^{37}ClN_4O_3S$	^{37}Cl-C11H15ClN4O3S
356.05786		354.04331		$C_{13}H_{14}ClN_5O_3S$	
358.05491		356.04036		$C_{13}H_{14}{}^{37}ClN_5O_3S$	

Table S4c: Exact masses of putative metabolites that were screened for after dosing acetamiprid (ACE) with given molecular formula in uncharged form. Based on the exact masses, *m/z* ratios for $[M+H]^+$ and $[M-H]^-$ are given. Masses that were added for confirmation by dd-MS^2 are marked with "x".

**Ionization mode**				**Formula [M]**	**Name**
**Positive $[M+H]^+$**		**Negative $[M-H]^-$**			
143.03705		141.0225		$C_6H_7ClN_2$	
144.02107		142.00652		C_6H_6ClNO	
145.0341		143.01955		$C_6H_7{}^{37}Cl\ N_2$	
146.01812		144.00356		$C_6H_6{}^{37}ClNO$	
157.05270		155.03815		$C_7H_9ClN_2$	
158.00033		155.98578		$C_6H_4NO_2$	^{35}Cl-6CNA
159.04975		157.03520		$C_7H_9{}^{37}ClN_2$	
159.99738		157.98283		$C_6H_4{}^{37}ClNO_2$	^{37}Cl-6CNA
170.02703		168.01247		$C_7H_8ClNO_2S$	
185.04762		183.03306		$C_8H_9ClN_2O$	
187.04467		185.03011		$C_8H_9{}^{37}ClN_2O$	
209.05885	x	207.04430	x	$C_9H_9ClN_4$	^{35}Cl-DME-ACE
211.05590	x	209.04135	x	$C_9H_9{}^{37}ClN_4$	^{37}Cl-DME-ACE
214.01685		212.00230		$C_8H_7NO_4S$	
215.02180	x	213.00724	x	$C_8H_7ClN_2O_3$	^{35}Cl-6CNA-glycine
217.01885	x	215.00429	x	$C_8H_7{}^{37}ClN_2O_3$	^{37}Cl-6CNA-glycine
223.07450	x	221.05995	x	$C_{10}H_{11}ClN_4$	^{35}Cl-ACE
225.07155	x	223.05700	x	$C_{10}H_{11}{}^{37}ClN_4$	^{37}Cl-ACE
227.06942		225.05486		$C_9H_{11}ClN_4O$	
229.06647		227.05191		$C_9H_{11}{}^{37}ClN_4O$	
239.06942		237.05486		$C_{10}H_{11}ClN_4O$	
241.06647		239.05191		$C_{10}H_{11}{}^{37}ClN_4O$	

Table S4d: Exact masses of putative metabolites that were screened for after dosing clothianidin (CLO) with given molecular formula in uncharged form. Based on the exact masses, *m/z* ratios for $[M+H]^+$ and $[M-H]^-$ are given. Masses that were added for confirmation by dd-MS^2 are marked with "x".

**Ionization mode**				**Formula [M]**	**Name**
**Positive $[M+H]^+$**		**Negative $[M-H]^-$**			
148.99347		146.97892		$C_4H_5ClN_2S$	
150.99052		148.97597		$C_4H_5{}^{37}ClN_2S$	
163.95675		161.94220		$C_4H_2ClNO_2S$	^{35}Cl-CTA
165.95380		163.93925		$C_4H_2{}^{37}ClNO_2S$	^{37}Cl-CTA
191.01527		189.00072		$C_5H_7ClN_4S$	
191.99929		189.98473		$C_5H_6ClN_3OS$	
193.01232		190.99777		$C_5H_7{}^{37}ClN_4S$	
193.99634		191.98178		$C_5H_6{}^{37}ClN_3OS$	
205.01969		203.00513		$C_7H_9ClN_2OS$	
205.03092		203.01637		$C_6H_9ClN_4S$	
206.01494	x	204.00038		$C_6H_8ClN_3OS$	^{35}Cl-CLO-urea
207.01674		205.00218		$C_7H_9{}^{37}ClN_2OS$	
207.02797		205.01342		$C_6H_9{}^{37}ClN_4S$	
208.01199	x	205.99743		$C_6H_8{}^{37}ClN_3OS$	^{37}Cl-CLO-urea
220.97822		218.96366		$C_6H_5ClN_2O_3S$	^{35}Cl-CTA-glycine
222.97527		220.96071		$C_6H_5{}^{37}ClN_2O_3S$	^{37}Cl-CTA-glycine
236.00035	x	233.98580	x	$C_5H_6ClN_5O_2S$	^{35}Cl-DME-CLO
237.99740	x	235.98285	x	$C_5H_6{}^{37}ClN_5O_2S$	^{37}Cl-DME-CLO
250.01600	x	248.00145	x	$C_6H_8ClN_5O_2S$	^{35}Cl-CLO
252.01305	x	249.99850	x	$C_6H_8{}^{37}ClN_5O_2S$	^{37}Cl-CLO
266.01091		264.99636		$C_6H_8ClN_5O_3S$	
268.00796		265.99341		$C_6H_8{}^{37}ClN_5O_3S$	
335.05907	x	333.04452	x	$C_9H_{14}N_6O_4{}^{32}S_2$	^{32}S-Cys-CLO
337.05487		335.04031		C $C_9H_{14}N_6O_4{}^{34}S_2$	^{34}S-Cys-CLO

Table S4e: Exact masses of putative metabolites that were screened for after dosing thiamethoxam (THIAM) with given molecular formula in uncharged form. Based on the exact masses, *m/z* ratios for $[M+H]^+$ and $[M-H]^-$ are given. Masses that were added for confirmation by dd-MS^2 are marked with "x".

**Ionization mode**				**Formula [M]**	**Name**
**Positive $[M+H]^+$**		**Negative $[M-H]^-$**			
147.05127		145.03671		$C_3H_6N_4O_3$	
148.99347		146.97892		$C_4H_5ClN_2S$	
150.99052		148.97597		$C_4H_5{}^{37}ClN_2S$	
163.95675		161.94220		$C_4H_2ClNO_2S$	^{35}Cl-CTA
165.95380		163.93925		$C_4H_2{}^{37}ClNO_2S$	^{37}Cl-CTA
191.01527		189.00072		$C_5H_7ClN_4S$	
191.99929		189.98473		$C_5H_6ClN_3OS$	
193.01232		190.99777		$C_5H_7{}^{37}ClN_4S$	
193.99634		191.98178		$C_5H_6{}^{37}ClN_3OS$	
205.01969		203.00513		$C_7H_9ClN_2OS$	
206.01494		204.00038		$C_6H_8ClN_3OS$	^{35}Cl-CLO-urea
207.01674		205.00218		$C_7H_9{}^{37}ClN_2OS$	
208.01199		205.99743		$C_6H_8{}^{37}ClN_3OS$	^{37}Cl-CLO-urea
220.97822		218.96366		$C_6H_5ClN_2O_3S$	^{35}Cl-CTA-glycine
222.97527		220.96071		$C_6H_5{}^{37}ClN_2O_3S$	^{37}Cl-CTA-glycine
236.00035	x	233.98580	x	$C_5H_6ClN_5O_2S$	^{35}Cl-DME-CLO
237.99740	x	235.98285	x	$C_5H_6{}^{37}ClN_5O_2S$	^{37}Cl-DME-CLO
247.04149	x	245.02693		$C_8H_{11}ClN_4OS$	^{35}Cl-THIAM-guanidine
248.02550	x	246.01095		$C_8H_{10}ClN_3O_2S$	^{35}Cl-THIAM-urea
249.03854	x	247.02398		$C_8H_{11}{}^{37}ClN_4OS$	^{37}Cl-THIAM-guanidine
250.01600	x	248.00145	x	$C_6H_8ClN_5O_2S$	^{35}Cl-CLO
250.02255	x	248.00800		$C_8H_{10}{}^{37}ClN_3O_2S$	^{37}Cl-THIAM-urea
252.01305	x	249.99850	x	$C_6H_8{}^{37}ClN_5O_2S$	^{37}Cl-CLO
278.01091		275.99636		$C_7H_8ClN_5O_3S$	^{35}Cl-DME-THIAM
280.00796		277.99341		$C_7H_8{}^{37}ClN_5O_3S$	^{37}Cl-DME-THIAM
292.02656		290.01201		$C_8H_{10}ClN_5O_3S$	^{35}Cl-THIAM
294.02361	x	292.00906		$C_8H_{10}{}^{37}ClN_5O_3S$	^{37}Cl-THIAM
377.06964	x	375.05508		$C_{11}H_{16}N_6O_5{}^{32}S_2$	^{32}S-Cys-THIAM
379.06543	x	377.05088		$C_{11}H_{16}N_6O_5{}^{34}S_2$	^{34}S-Cys-THIAM

Table S4f: Exact masses of putative metabolites that were screened for after dosing flupyradifurone (FLUP) with given molecular formula in uncharged form. Based on the exact masses, *m/z* ratios for $[M+H]^+$ and $[M-H]^-$ are given. Masses that were added for confirmation by dd-MS^2 are marked with "x".

**Ionization mode**				**Formula [M]**	**Name**
**Positive $[M+H]^+$**		**Negative $[M-H]^-$**			
143.03705		141.02250		$C_6H_7ClN_2$	
144.02107		142.00652		C_6H_6ClNO	
145.03410		143.01955		$C_6H_7{}^{37}Cl\ N_2$	
146.01812		144.00356		$C_6H_6{}^{37}ClN_O$	
158.00033		155.98578	x	$C_6H_4ClNO_2$	^{35}Cl-6CNA
159.99738		157.98283	x	$C_6H_4{}^{37}ClNO_2$	^{37}Cl-6CNA
164.05176	x	162.03721	x	$C_6H_7F_2NO_2$	DCP-FLUP
215.02180	x	213.00724	x	$C_8H_7ClN_2O_3$	^{35}Cl-6CNA-glycine
217.01885	x	215.00429	x	$C_8H_7{}^{37}ClN_2O_3$	^{37}Cl-6CNA-glycine
225.04253	x	223.02798	x	$C_{10}H_9ClN_2O_2$	^{35}Cl-DFE-FLUP
227.03958	x	225.02503	x	$C_{10}H_9{}^{37}ClN_2O_2$	^{37}Cl-DFE-FLUP
289.05499	x	287.04044	x	$C_{12}H_{11}ClF_2N_2O_3$	^{35}Cl-FLUP
291.05204	x	289.03749	x	$C_{12}H_{11}{}^{37}ClF_2N_2O_3$	^{37}Cl-FLUP
305.04995	x	303.03535	x	$C_{12}H_{11}ClF_2N_2O_4$	^{35}Cl-OH-FLUP
307.04695	x	305.03240	x	$C_{12}H_{11}{}^{37}ClF_2N_2O_4$	^{37}Cl-OH-FLUP

Table S4g: Exact masses of putative metabolites that were screened for after dosing sulfoxaflor (SULF) with given molecular formula in uncharged form. Based on the exact masses, *m/z* ratios for $[M+H]^+$ and $[M-H]^-$ are given. Masses that were added for confirmation by dd-MS^2 are marked with "x".

Ionization mode				Formula [M]	Name
Positive $[M+H]^+$		Negative $[M-H]^-$			
178.04742		176.03287		$C_7H_6F_3NO$	
192.02669		190.01214		$C_7H_4F_3NO_2$	
192.06307		190.04852		$C_8H_8F_3NO$	
249.04815		247.03360		$C_9H_7F_3N_2O_3$	
264.04129		262.02674		$C_9H_7F_3N_3OS$	
278.05694	x	276.04239	x	$C_{10}H_{10}F_3N_3OS$	SULF
294.05186		292.05186		$C_{10}H_{10}F_3N_3O_2S$	
296.06751		294.05296		$C_{10}H_{12}F_3N_3O_2S$	

Figure S1a: Urinary excretion kinetics for imidacloprid (IMI), hydroxy-IMI (OH-IMI), and IMI-olefin presenting both, ^{35}Cl- (bold line) and ^{37}Cl-isotopologs (dashed line) obtained by LC-Q-Orbitrap-MS analysis after an oral dose of 5 mg IMI. Left: unadjusted absolute peak areas; middle: creatinine-adjusted peak areas; right: excretion rate adjusted for sample volume and the time frame covered by the urine sample [counts-seconds*mL/h].

IMI (ESI+)

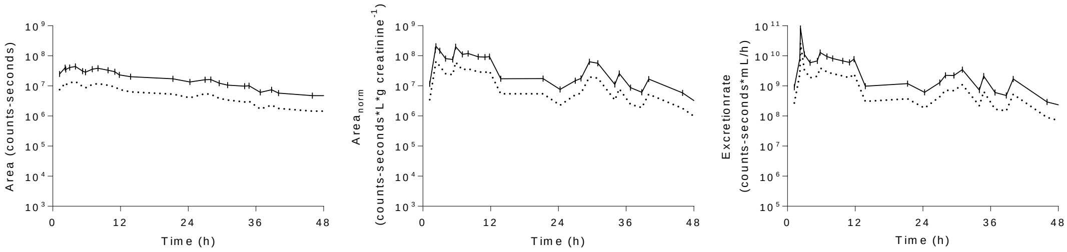

OH-IMI (ESI+)

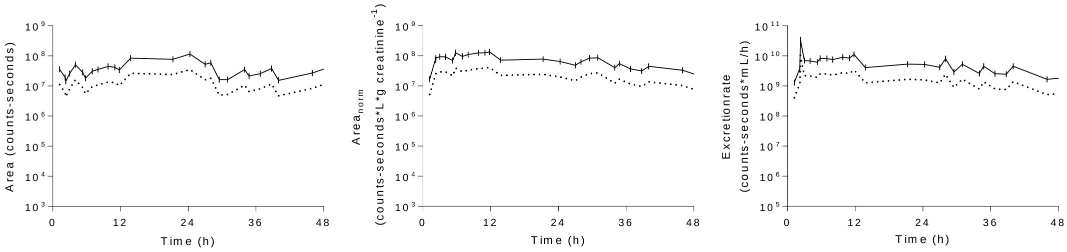

IMI-olefin (ESI+)

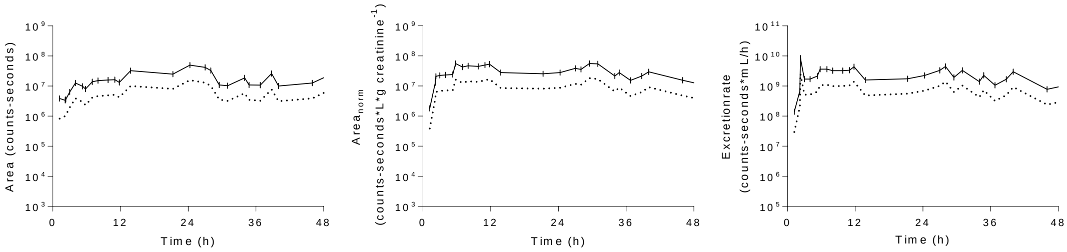

6-CNA-gly (ESI-)

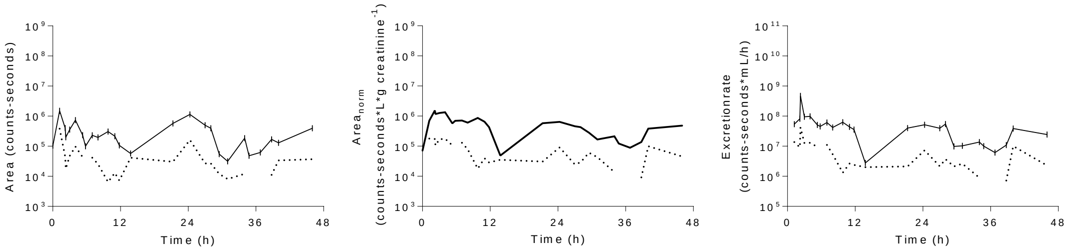

Figure S1b: Urinary excretion kinetics for thiacloprid (THIAC), hydroxy-THIAC-olefin (OH-THIAC-olefin), and OH-THIAC presenting both, ^{35}Cl- (bold line) and ^{37}Cl-isotopologs (dashed line) obtained by LC-Q-Orbitrap-MS analysis after an oral dose of 1 mg THIAC. Left: unadjusted absolute peak areas; middle: creatinine-adjusted peak areas; right: excretion rate adjusted for sample volume and the time frame covered by the urine sample [counts-seconds*mL/h].

THIAC (ESI+)

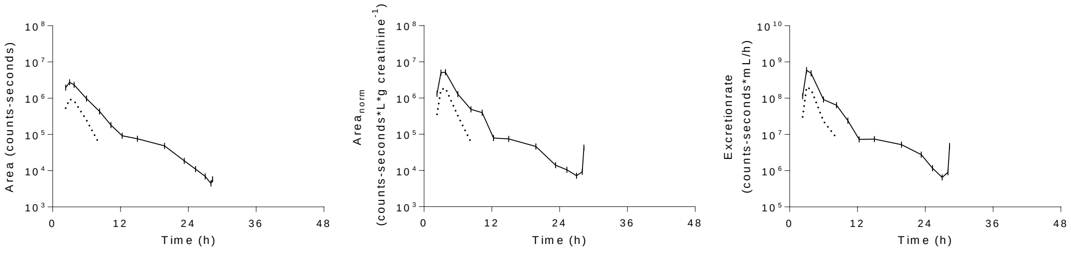

OH-THIAC-olefin (ESI-)

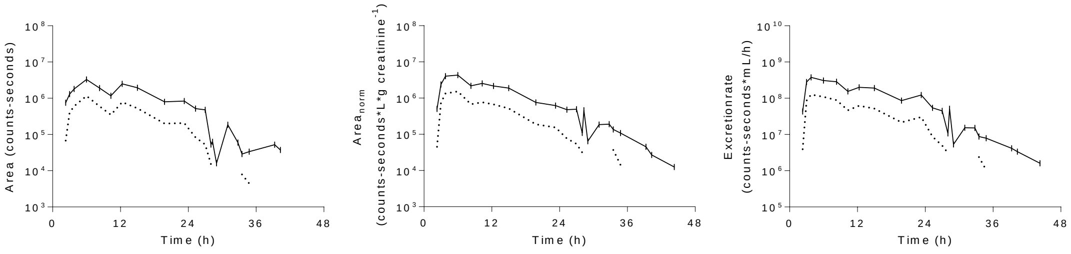

OH-THIAC 8.8 + 8.9 min (ESI+)

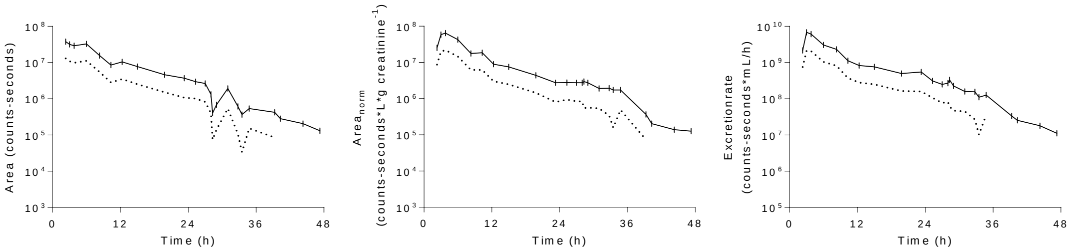

OH-THIAC 10.5 min (ESI-)

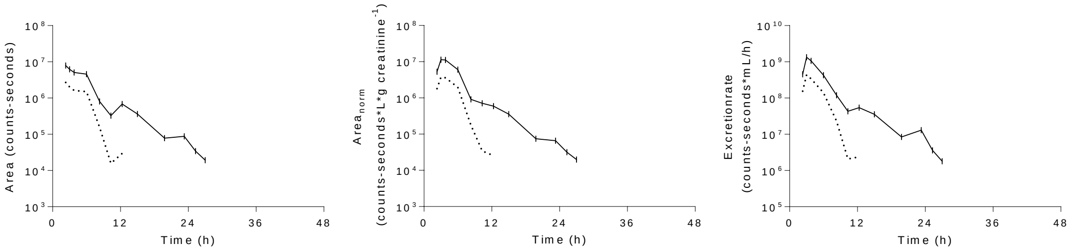

Figure S1b: continued

THIAC-amide (ESI-)

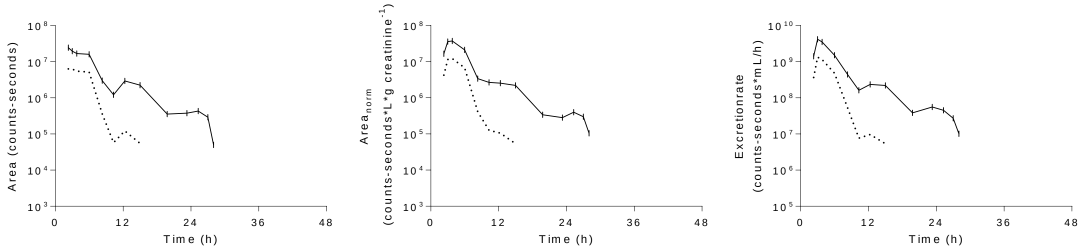

6-CNA-gly (ESI-)

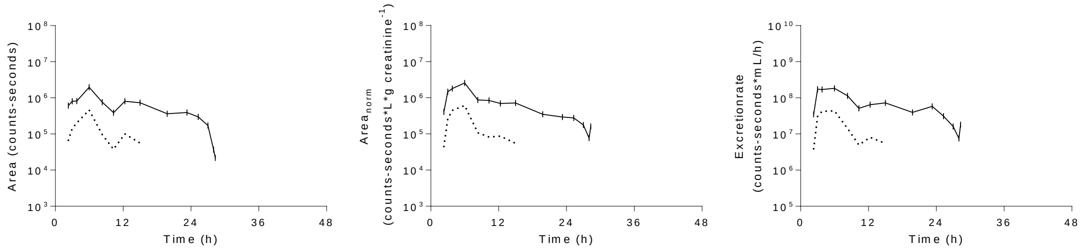

C11H15ClN4O3S (ESI+)

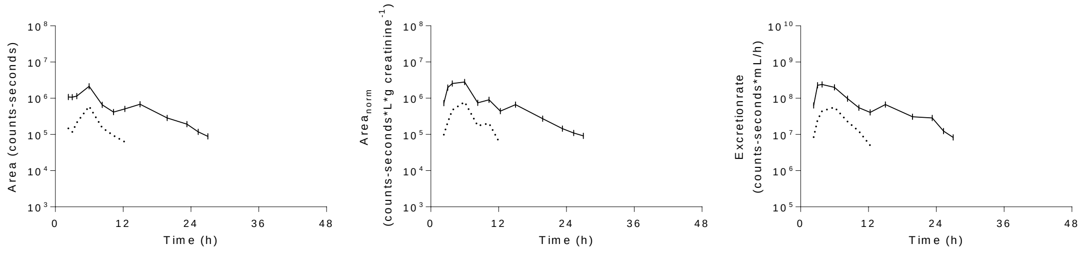

Figure S1c: Urinary excretion kinetics for acetamiprid (ACE), desmethyl-ACE (DME-ACE), and 6-chloro nicotinoyl glycine (6-CNA-gly) presenting both, ^{35}Cl- (bold line) and ^{37}Cl-isotopologs (dashed line) obtained by LC-Q-Orbitrap-MS analysis after an oral dose of 2 mg ACE. Left: unadjusted absolute peak areas; middle: creatinine-adjusted peak areas; right: excretion rate adjusted for sample volume and the time frame covered by the urine sample [counts-seconds*mL/h].

ACE (ESI+)

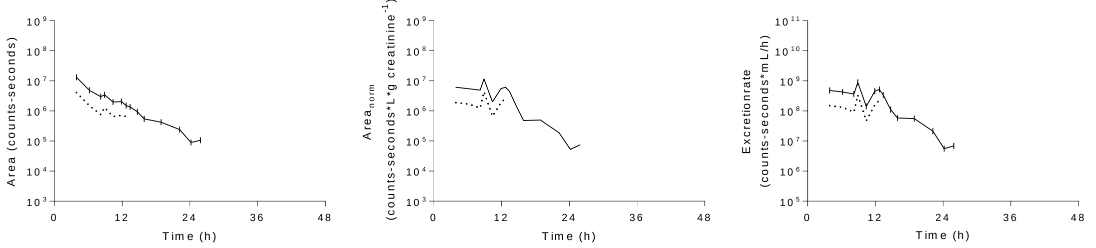

DME-ACE (ESI-)

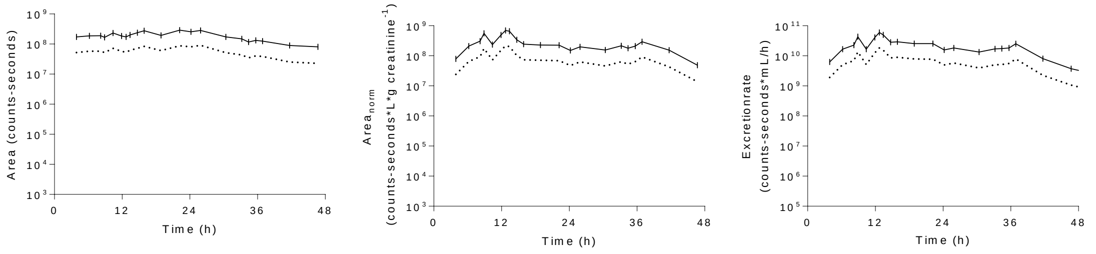

6-CNA-gly (ESI-)

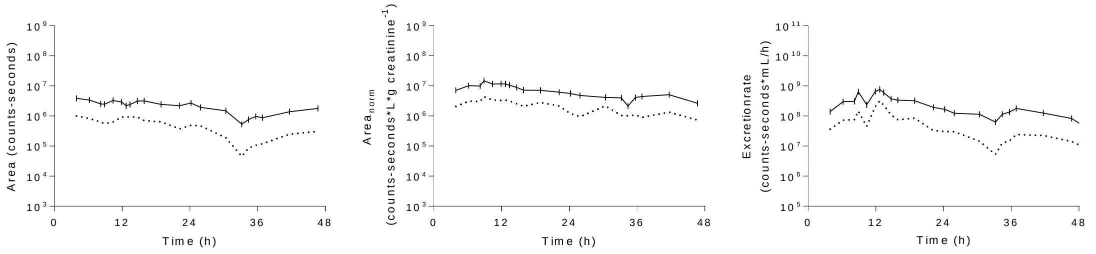

Figure S1d: Urinary excretion kinetics for thiamethoxam (THIAM), clothianidin (CLO), desmethyl-CLO (DME-CLO), and cysteinyl-THIAM presenting, if possible, both, ^{35}Cl- (bold line) and ^{37}Cl-isotopologs (dashed line) obtained by LC-Q-Orbitrap-MS analysis after oral dose of 2 mg THIAM. In case of cysteinyl-THIAM (absence of chlorine), the dashed lines represent the ^{34}S isotope signal masses. Left: unadjusted absolute peak areas; middle: creatinine-adjusted peak areas; right: excretion rate adjusted for sample volume and the time frame covered by the urine sample [counts-seconds*mL/h].

THIAM (ESI+)

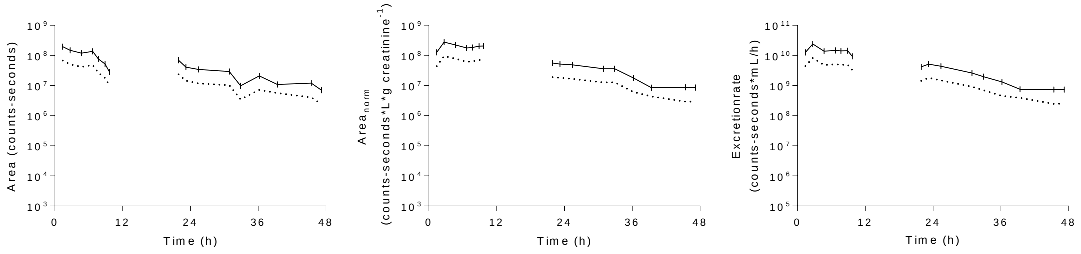

CLO (ESI-)

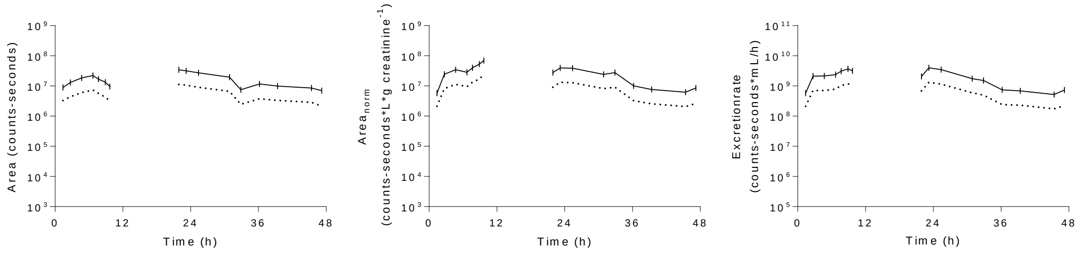

DME-CLO (ESI-)

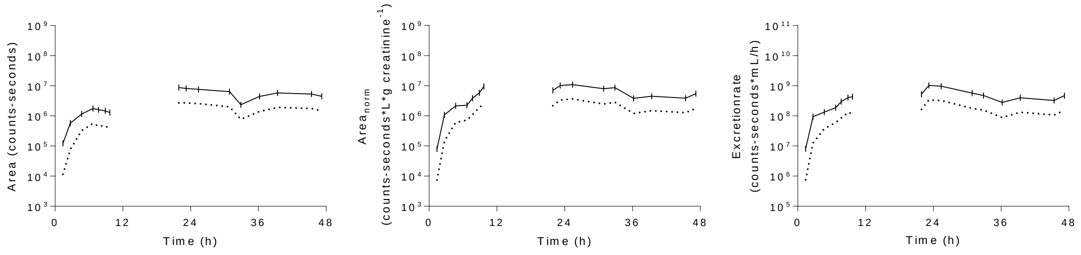

Cysteinyl-THIAM (ESI+)

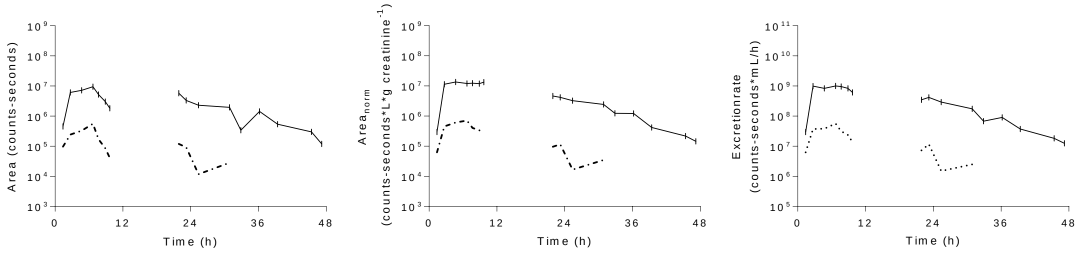

Figure S1e: Urinary excretion kinetics for clothianidin (CLO), desmethyl-CLO (DME-CLO), CLO-urea and cysteinyl-CLO presenting, if possible, both, ^{35}Cl- (bold line) and ^{37}Cl-isotopologs (dashed line) obtained by LC-Q-Orbitrap-MS analysis after an oral dose of 5 mg CLO. Left: unadjusted absolute peak areas; middle: creatinine-adjusted peak areas; right: excretion rate adjusted for sample volume and the time frame covered by the urine sample [counts-seconds*mL/h].

CLO (ESI-)

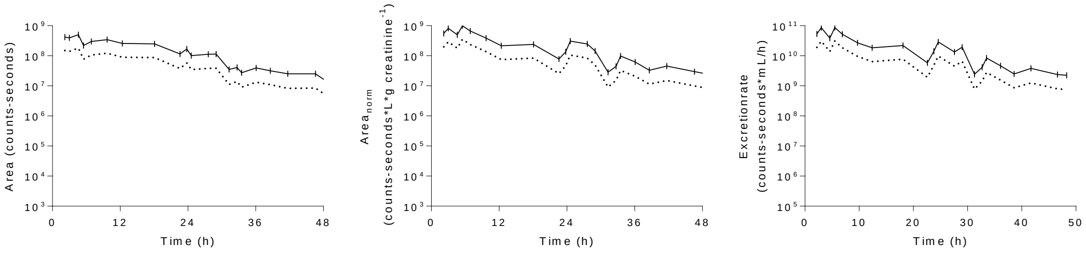

DME-CLO (ESI-)

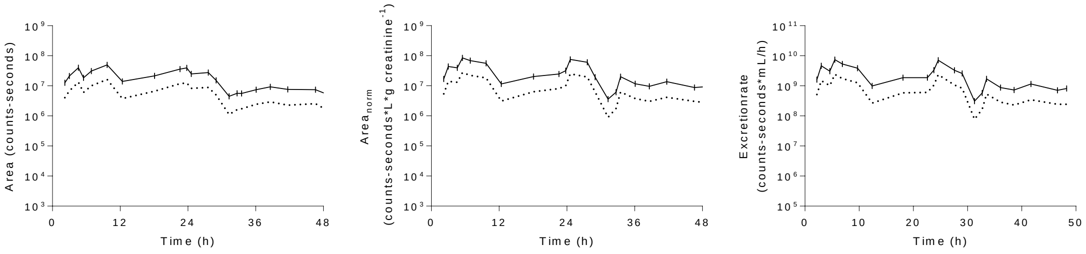

CLO-urea (ESI+)

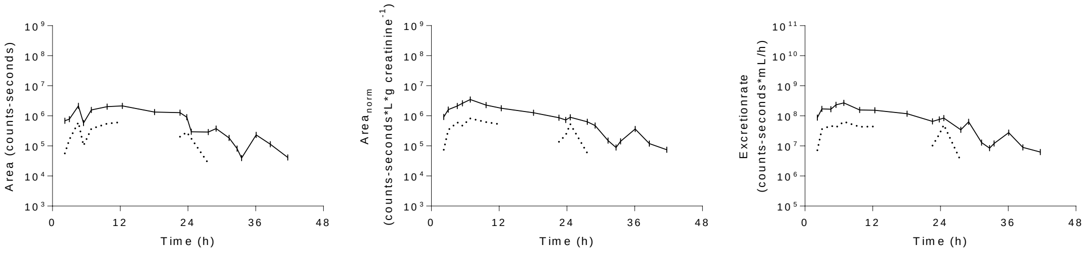

Cysteinyl-CLO (ESI+)

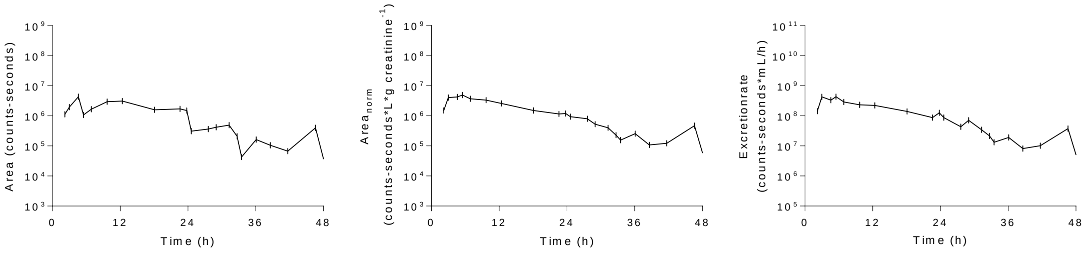

Figure S1f: Urinary excretion kinetics for flupyradifurone (FLUP) and three hydroxy-FLUP (OH-FLUP) isomers presenting both, ^{35}Cl- (bold line) and ^{37}Cl-isotopologs (dashed line) obtained by LC-Q-Orbitrap-MS analysis after oral dose of 5 mg FLUP. Left: unadjusted absolute peak areas; middle: creatinine-adjusted peak areas; right: excretion rate adjusted for sample volume and the time frame covered by the urine sample [counts-seconds*mL/h].

FLUP (ESI+)

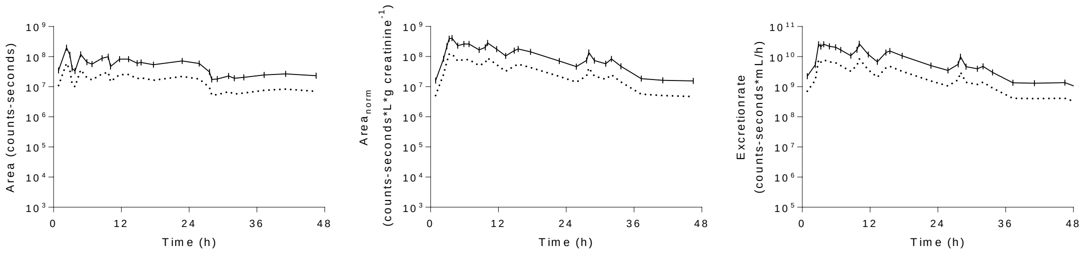

OH-FLUP 9.2 min (ESI+)

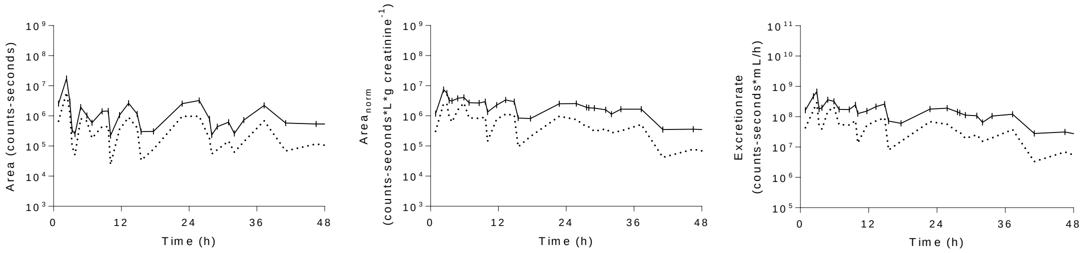

OH-FLUP 9.3 min (ESI+)

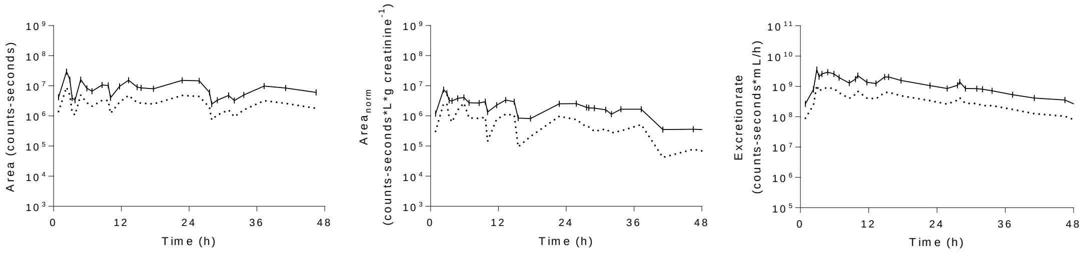

OH-FLUP 9.8 min (ESI+)

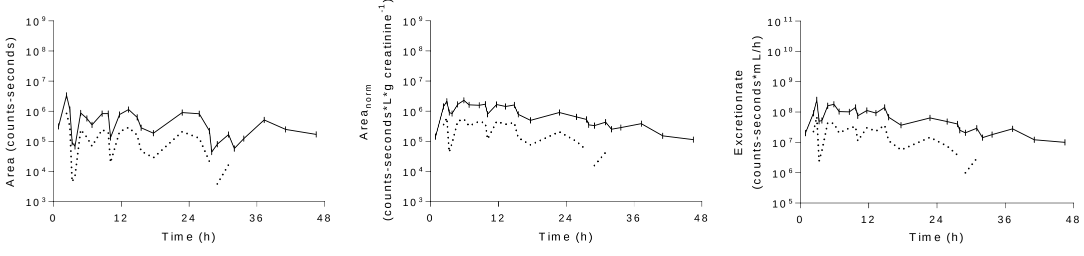

Figure S1f: continued

DFE-FLUP (ESI+)

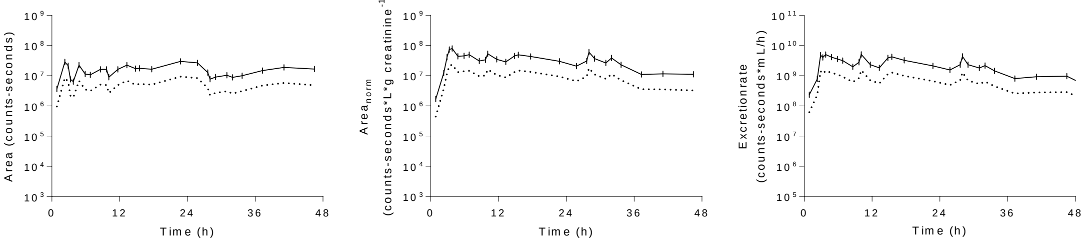

6-CNA-gly (ESI-)

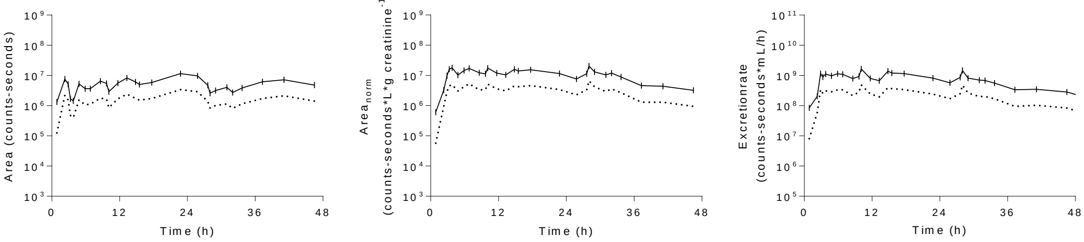

DCP-FLUP (ESI-)

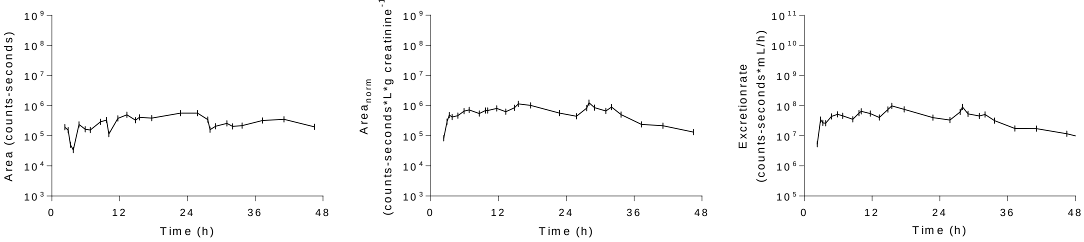

Figure S1g: Urinary excretion kinetics for sulfoxaflor (SULF), presenting, if possible, both, ^{32}S- (bold line) and ^{34}S-isotopologs (dashed line) obtained by LC-Q-Orbitrap-MS analysis after oral dose of 3 mg SULF. Left column: unadjusted absolute peak areas; middle: creatinine-adjusted peak areas; right column: excretionrate obtained by multiplying by the sample volume and dividing by the sampling time.

SULF (ESI-)

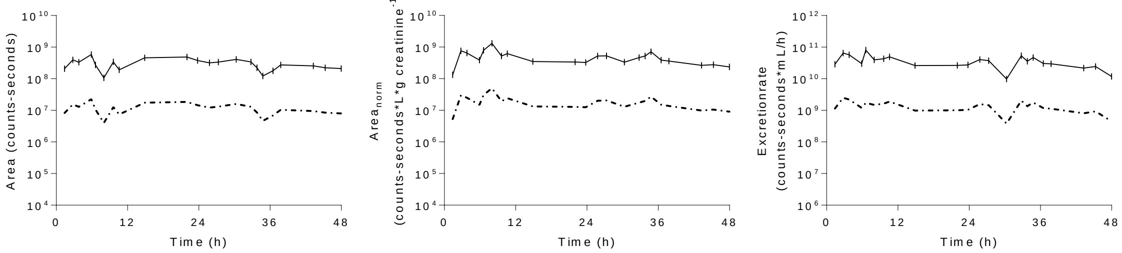

Figure S2a: Confirmation of potential urinary metabolites after a single oral dose of 5 mg imidacloprid (IMI). For each putative metabolite, the extracted ion chromatogram of the ^{35}Cl-isotopolog is shown (upper left corner), its isotope cluster region (MS^1 full scan data, lower left) and the product ion spectrum (dd-MS^2 acquisition, right panel); hypotheses for fragmentation (if possible) are presented; inexplicable masses (mass fragments) are presented in *italics*.

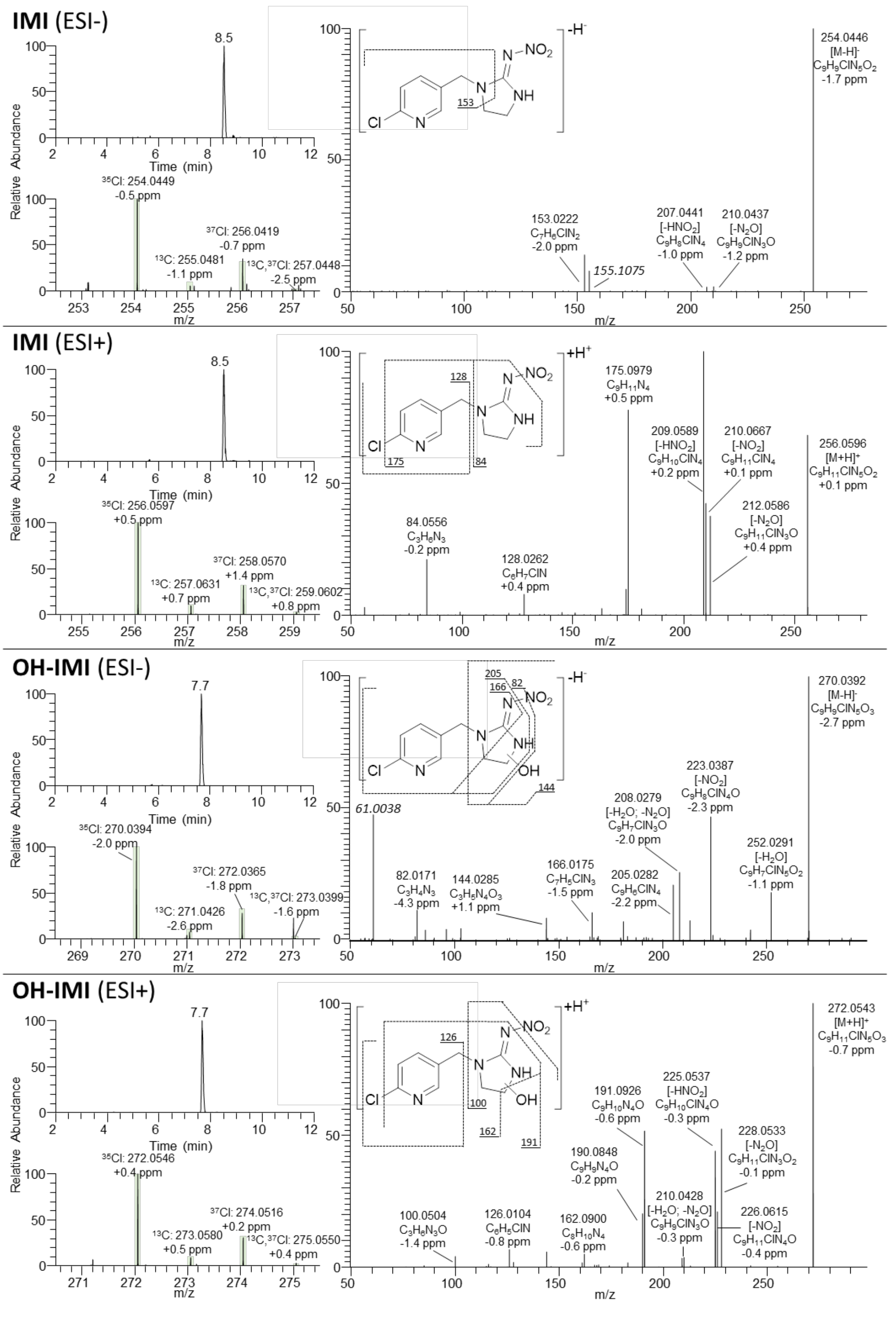

Figure S2a: continued.

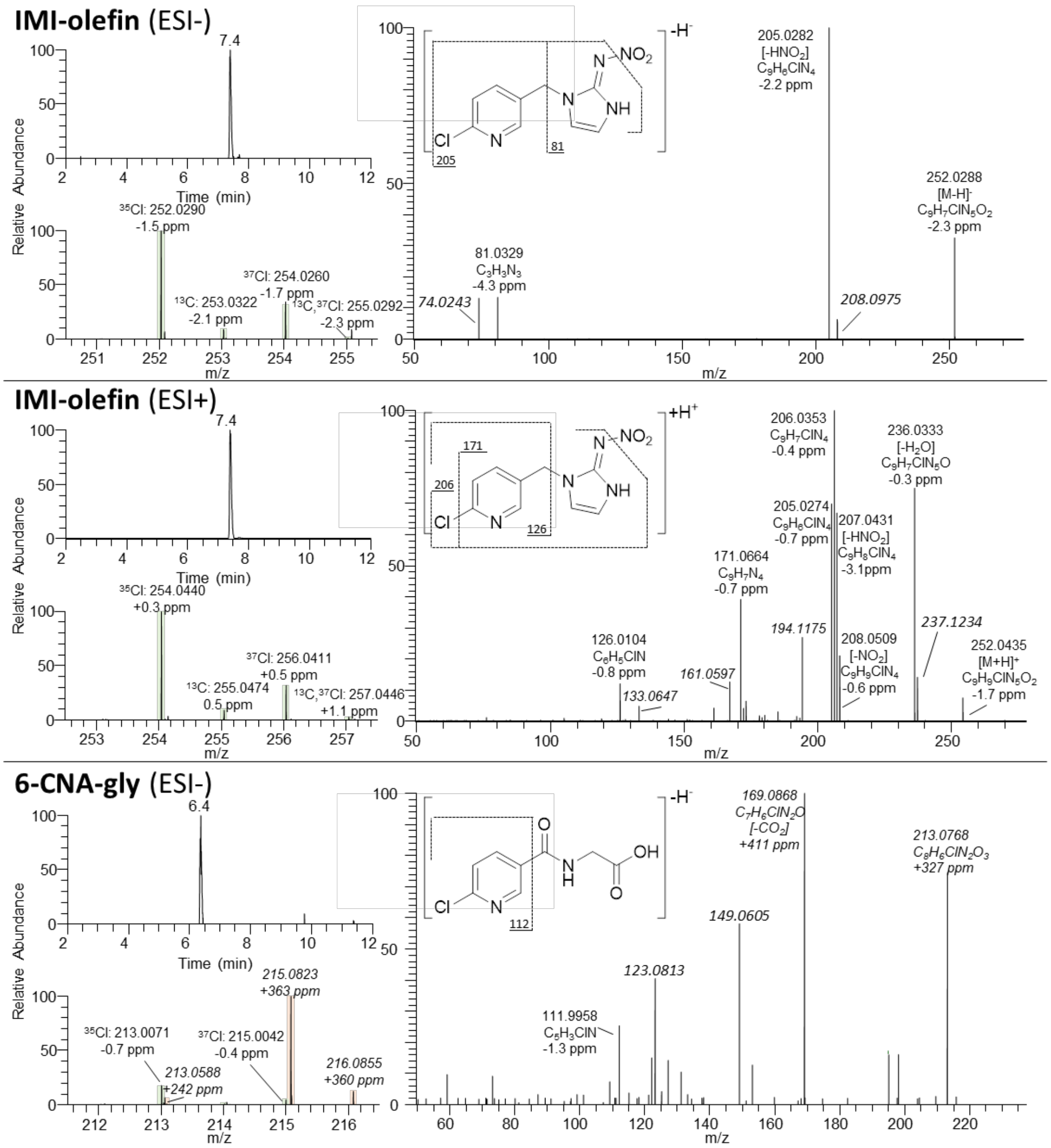

Figure S2b: Confirmation of potential urinary metabolites after a single oral dose of 1 mg thiacloprid (THIAC). For each putative metabolite, the extracted ion chromatogram of the ^{35}Cl-isotopolog is shown (upper left corner), its isotope cluster region (MS^1 full scan data, lower left) and the product ion spectrum (dd-MS² acquisition, right panel); hypotheses for fragmentation (if possible) are presented; inexplicable masses (mass fragments) are presented in *italics*.

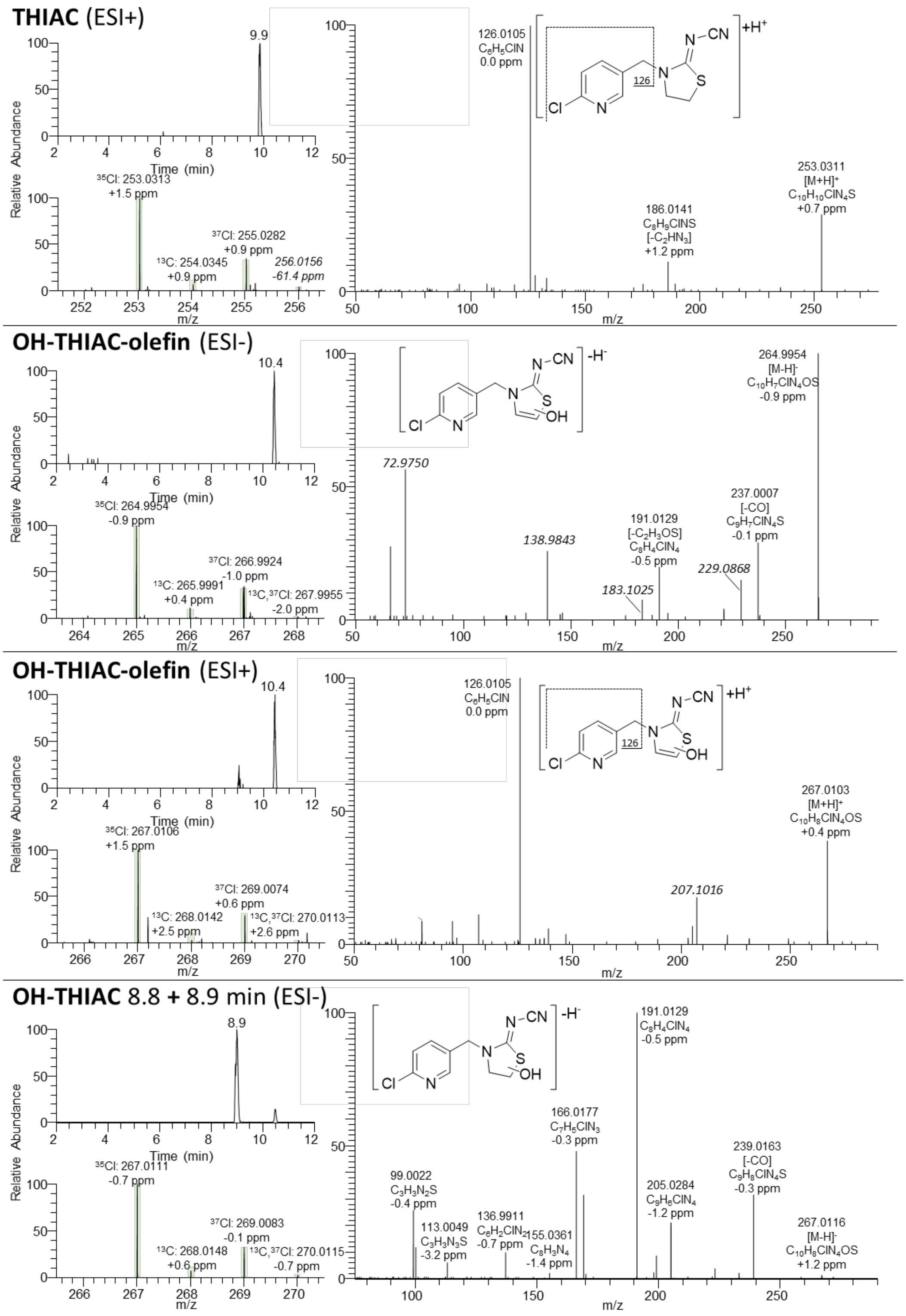

Figure S2b: continued.

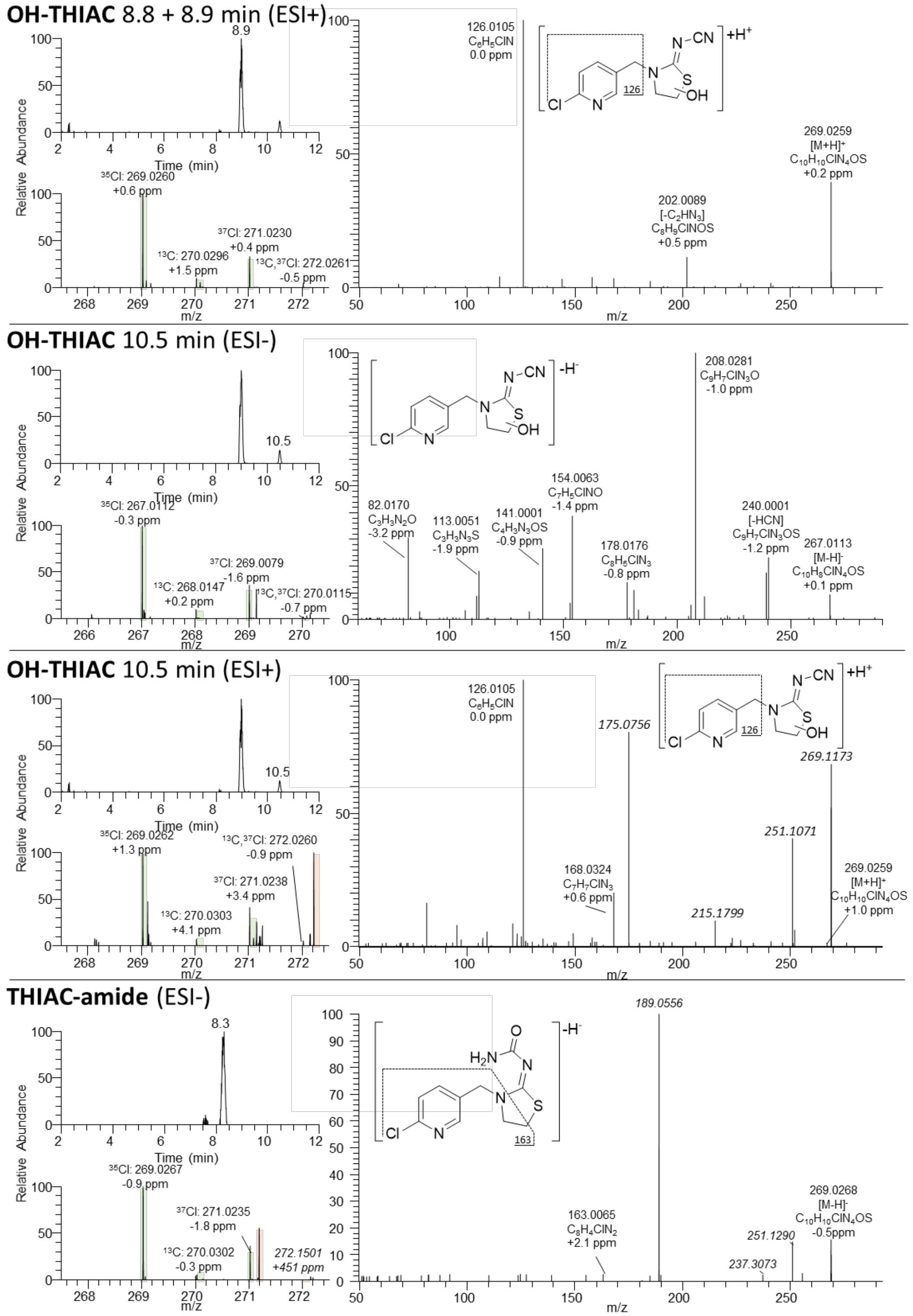

Figure 2b: continued.

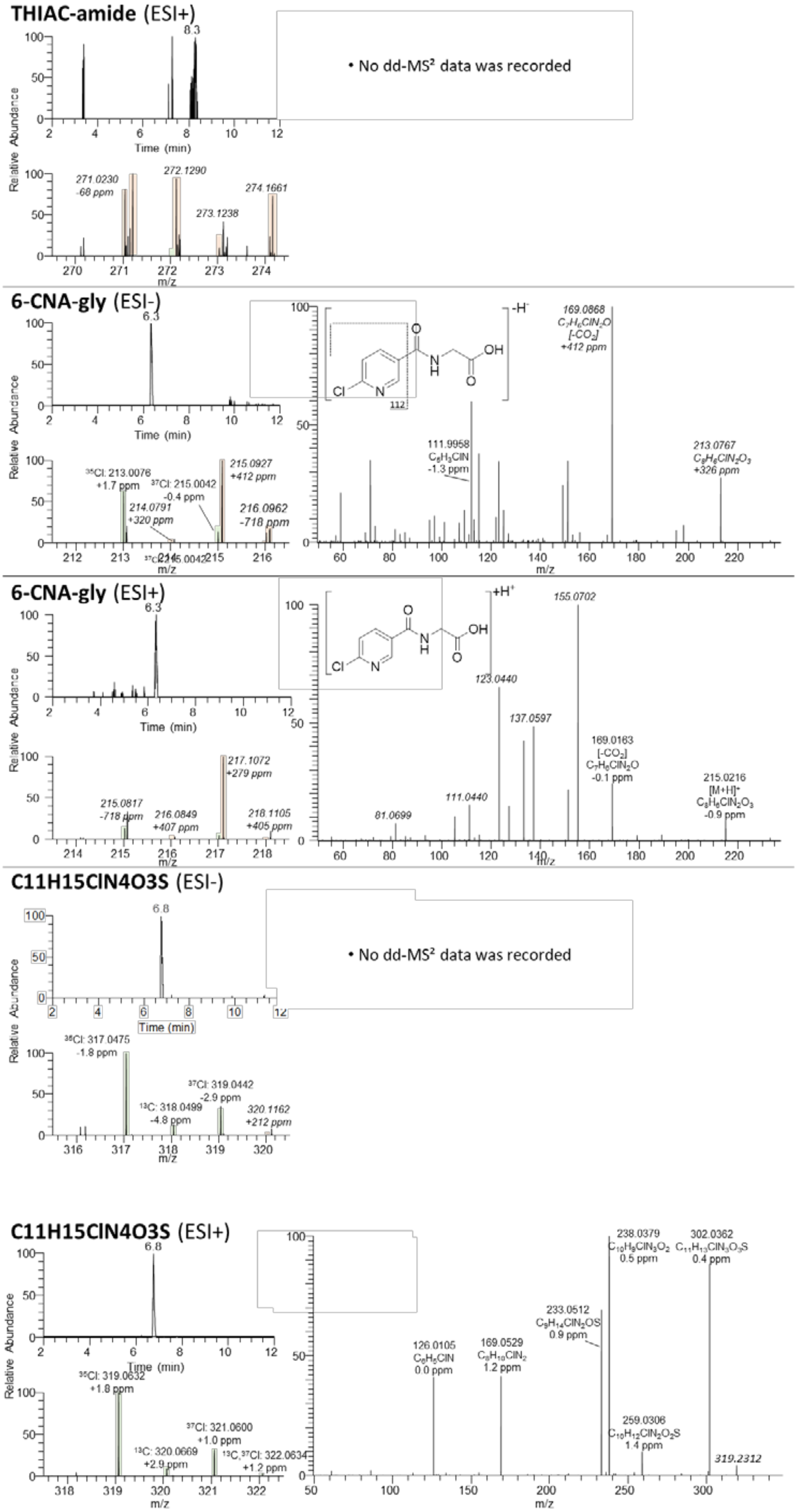

Figure 2c: Confirmation of potential urinary metabolites after a single oral dose of 2 mg acetamiprid (ACE). For each putative metabolite, the extracted ion chromatogram of the ^{35}Cl-isotopolog is shown (upper left corner), its isotope cluster region (MS^1 full scan data, lower left) and the product ion spectrum (dd-MS^2 acquisition, right panel); hypotheses for fragmentation (if possible) are presented; inexplicable masses (mass fragments) are presented in *italics*.

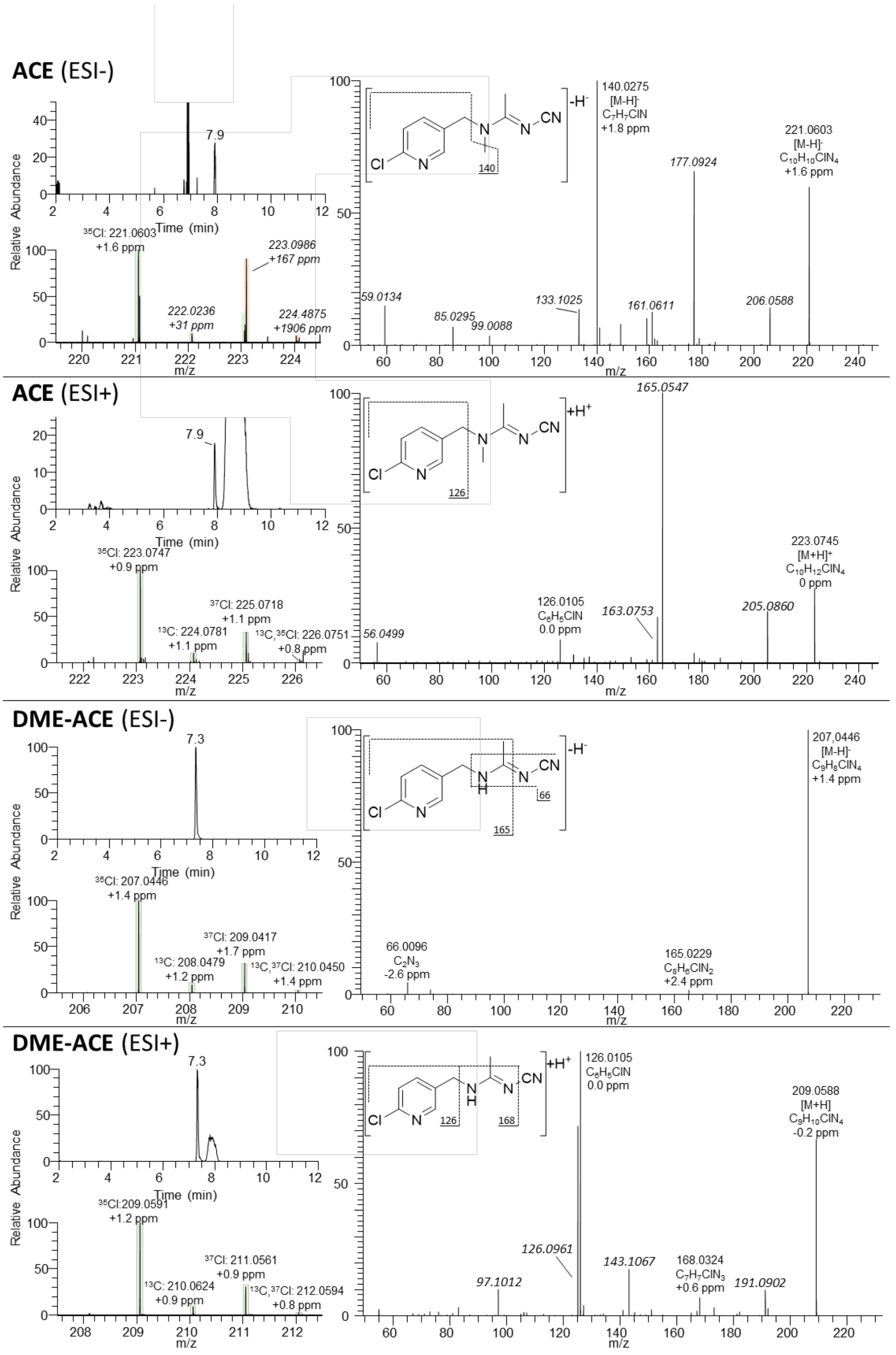

Figure 2c: continued.

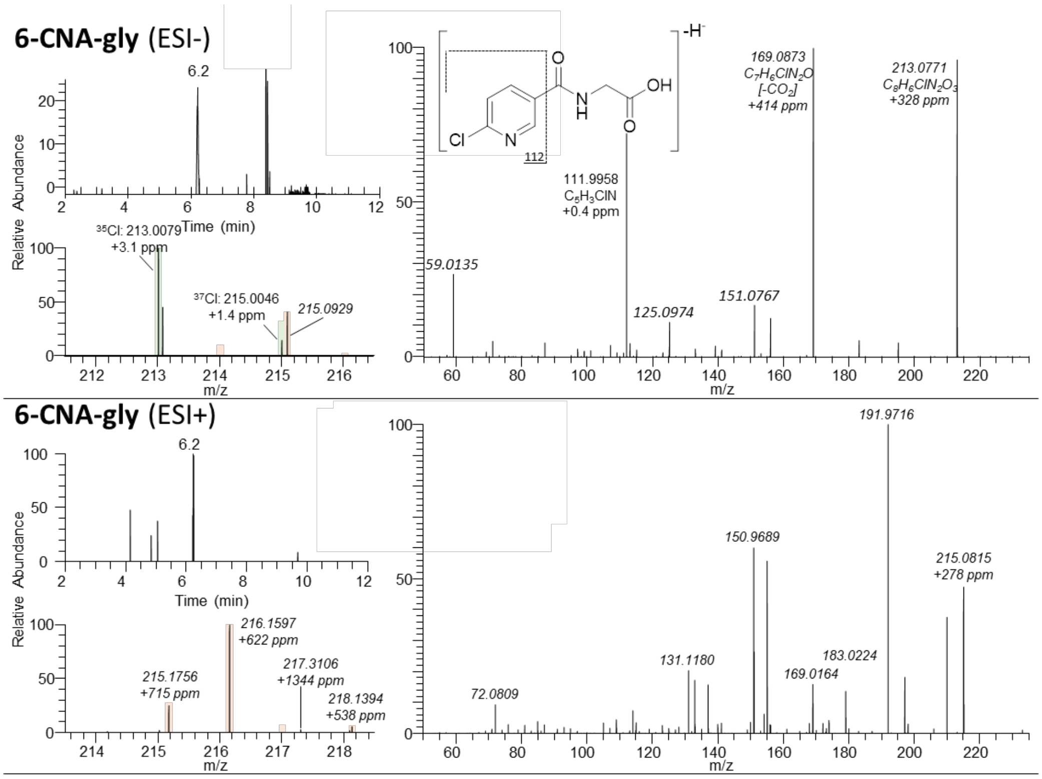

Figure S2d: Confirmation of potential urinary metabolites after a single oral dose of 2 mg thiamethoxam (THIAM). For each putative metabolite, the extracted ion chromatogram of the ^{35}Cl-isotopolog is shown (upper left corner), its isotope cluster region (MS^1 full scan data, lower left) and the product ion spectrum (dd-MS^2 acquisition, right panel); hypotheses for fragmentation (if possible) are presented; inexplicable masses (mass fragments) are presented in *italics*.

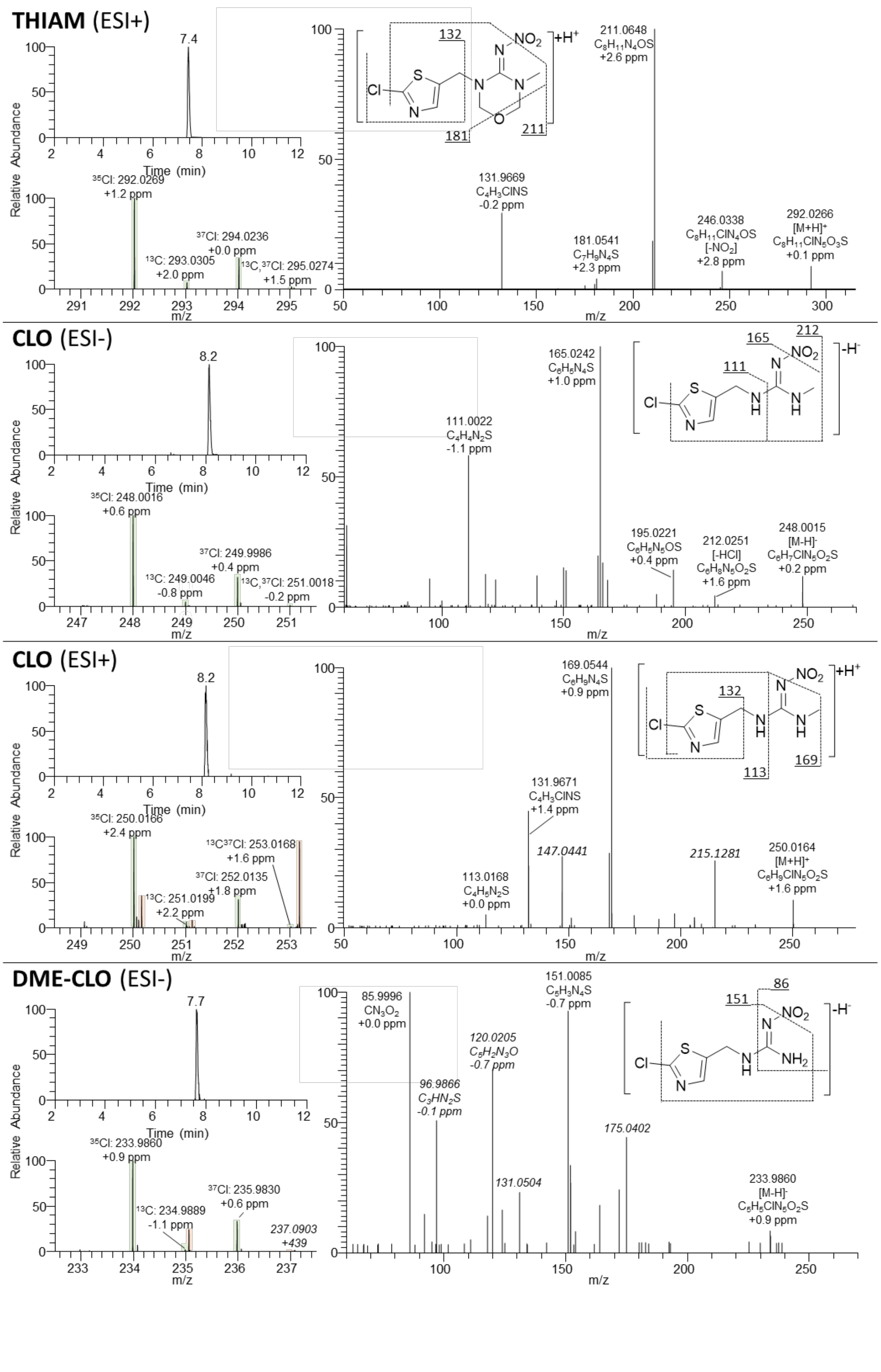

Figure S2d: continued.

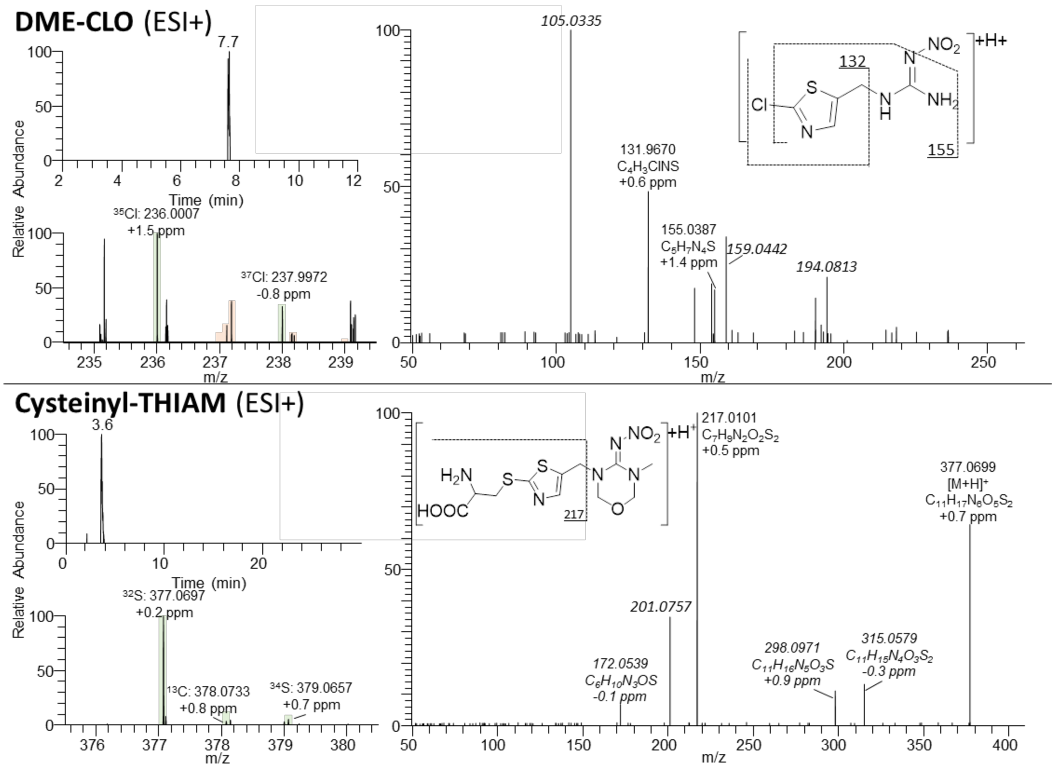

Figure S2e: Confirmation of potential urinary metabolites after a single oral dose of 2 mg clothianidin (CLO). For each putative metabolite, the extracted ion chromatogram of the ^{35}Cl-isotopolog is shown (upper left corner), its isotope cluster region (MS[1] full scan data, lower left) and the product ion spectrum (dd-MS² acquisition, right panel); hypotheses for fragmentation (if possible) are presented; inexplicable masses (mass fragments) are presented in *italics*.

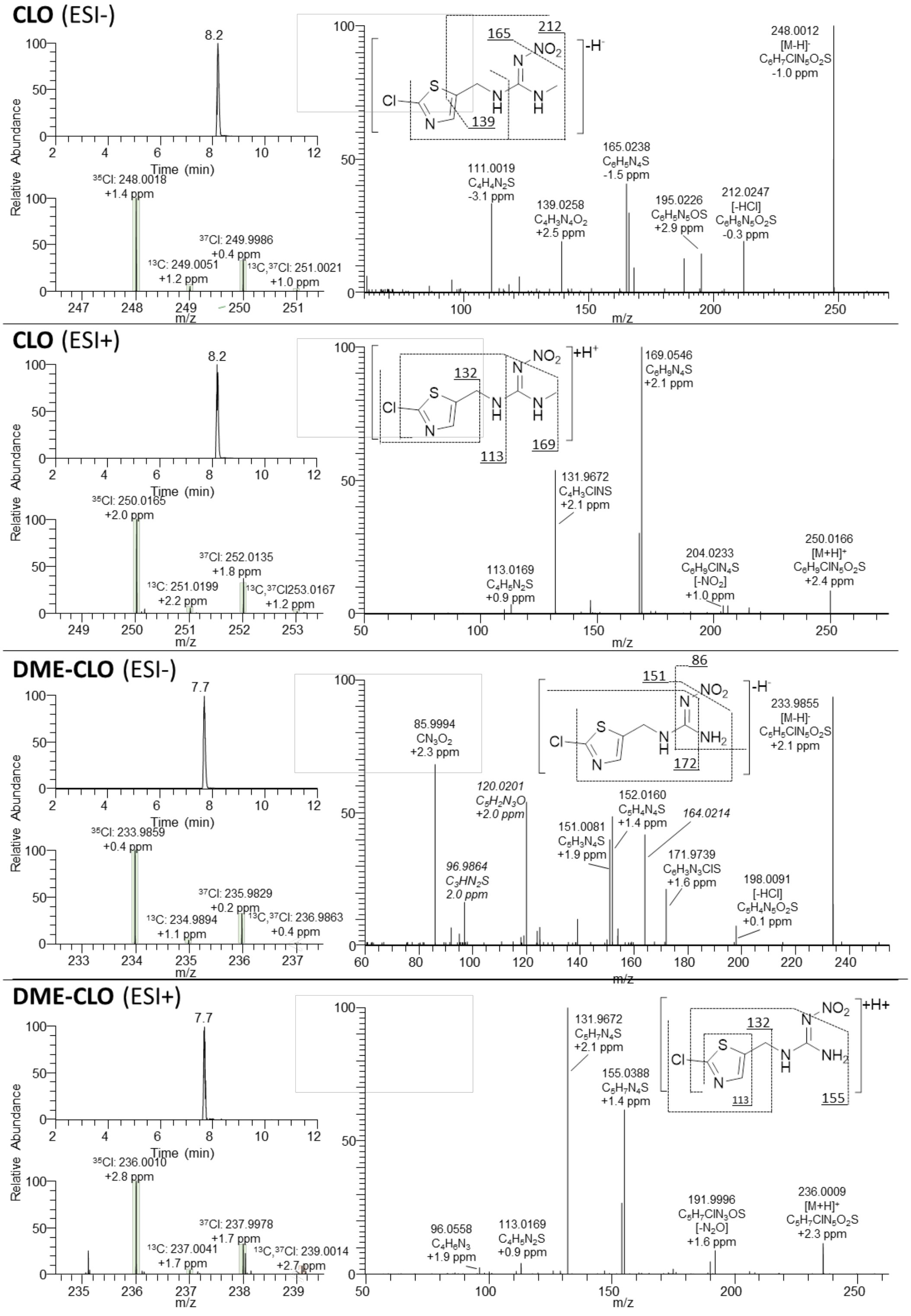

Figure S2e: continued.

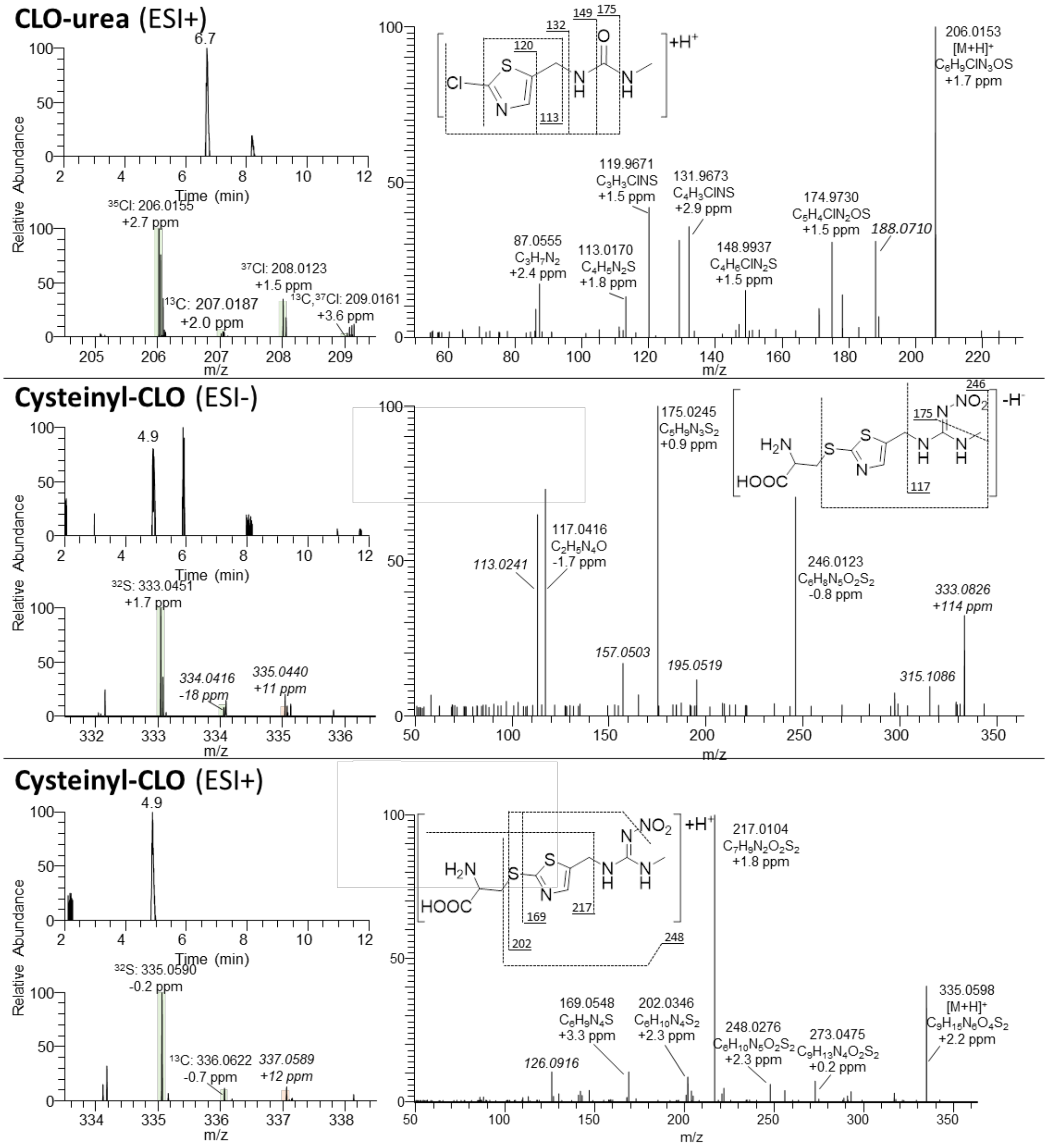

Figure S2f: Confirmation of potential urinary metabolites after a single oral dose of 5 mg flupyradifurone (FLUP). For each putative metabolite, the extracted ion chromatogram of the ^{35}Cl-isotopolog is shown (upper left corner), its isotope cluster region (MS[1] full scan data, lower left) and the product ion spectrum (dd-MS² acquisition, right panel); hypotheses for fragmentation (if possible) are presented; inexplicable masses (mass fragments) are presented in *italics*.

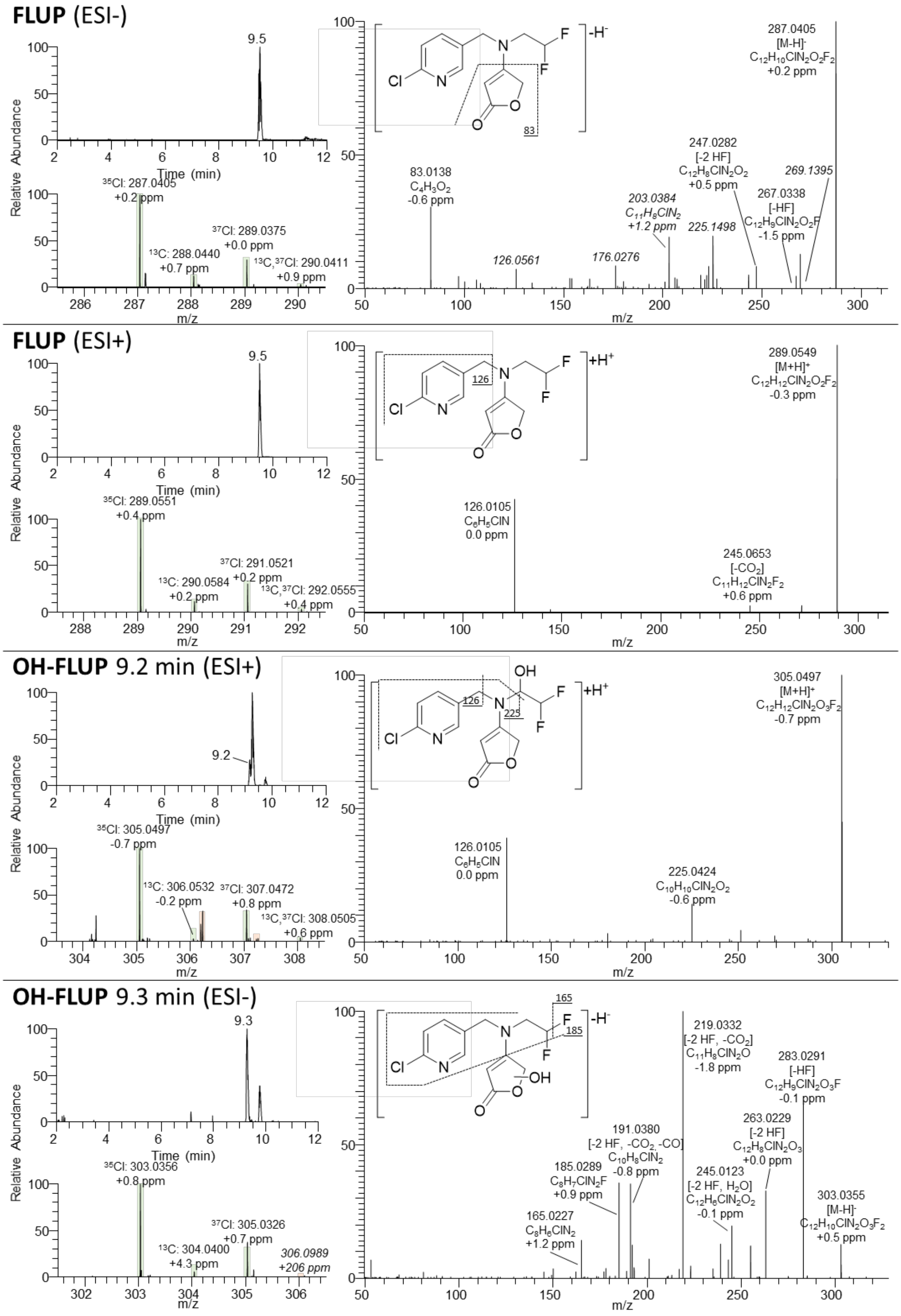

Figure S2f: continued

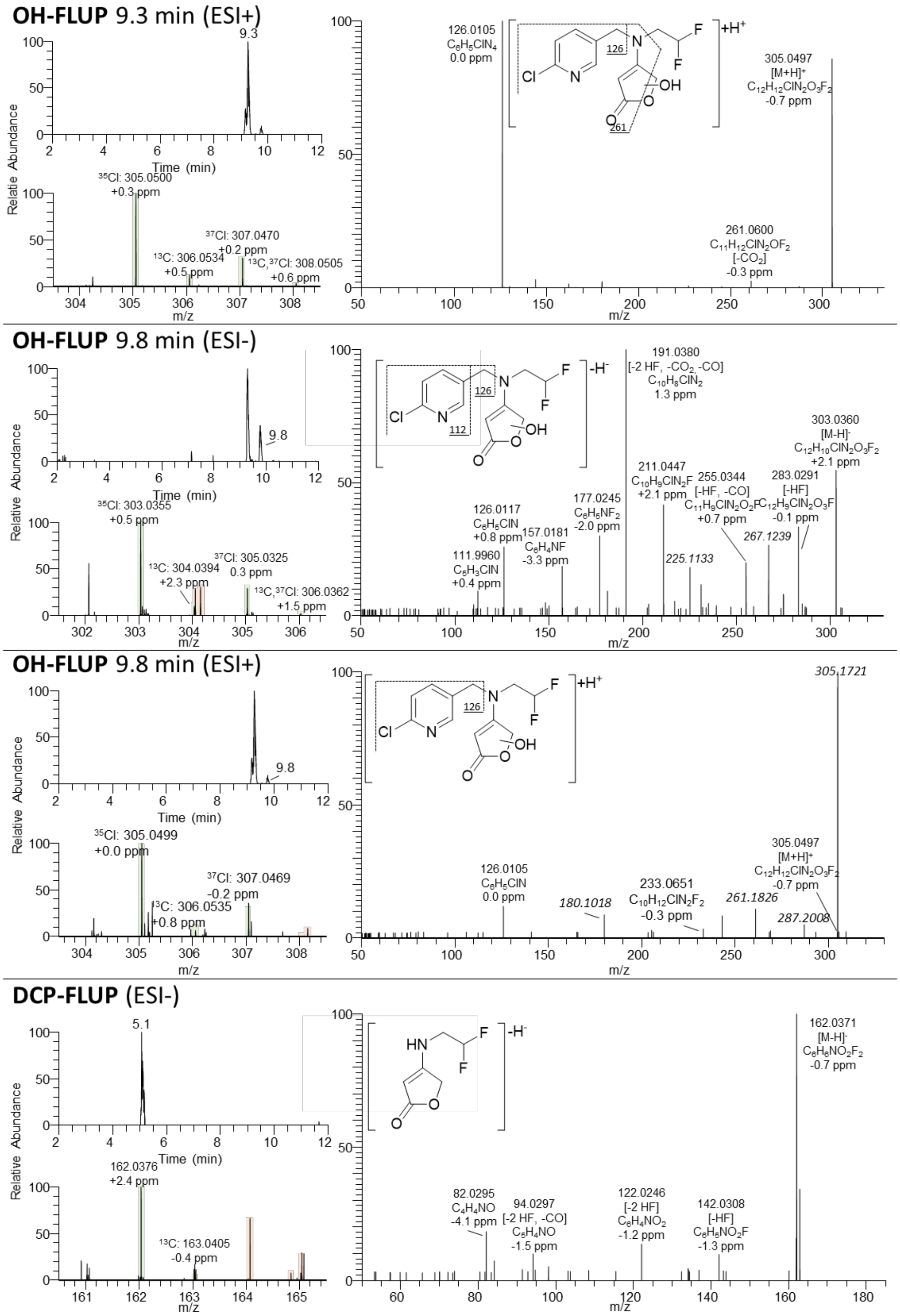

Figure S2f: continued.

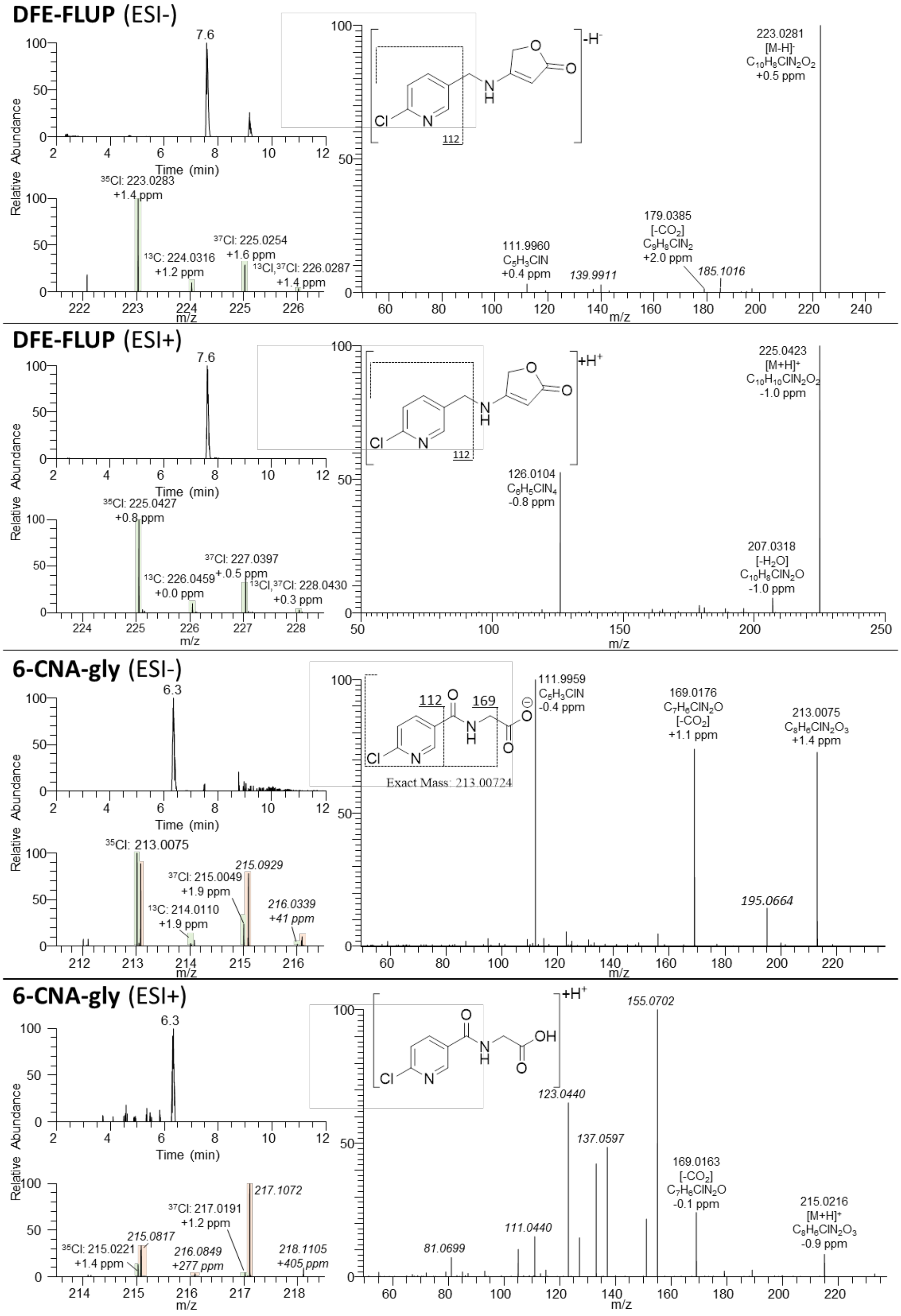

Figure S2g: Confirmation of potential urinary metabolites after a single oral dose of 3 mg sulfoxaflor (SULF). For each putative metabolite, the extracted ion chromatogram of the ^{35}Cl-isotopolog is shown (upper left corner), its isotope cluster region (MS^1 full scan data, lower left) and the product ion spectrum (dd-MS² acquisition, right panel); hypotheses for fragmentation (if possible) are presented; inexplicable masses (mass fragments) are presented in *italics*.

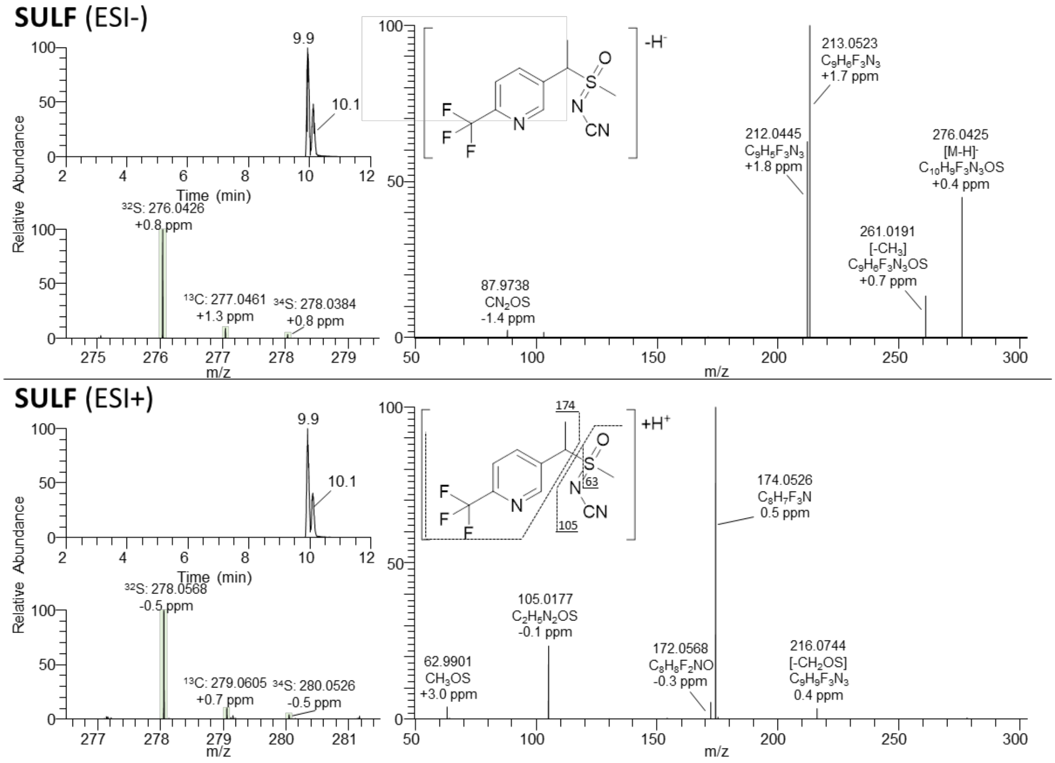

Figure S3a: Pearson correlation between the peak areas in the positive and the negative ionization mode if the corresponding accurate mass was detectable in both ionization modes (left column) and pearson correlation between the peak areas of the $^{35}Cl/^{37}Cl$-isotopologs either from positive or in negative ionization mode for imidacloprid (IMI), hydroxy-IMI (OH-IMI), and IMI-olefin obtained by LC-Q-Orbitrap-MS analysis after oral dose of 5 mg imidacloprid. Based on the relative abundance of the ^{35}Cl- and ^{37}Cl- isotopologs, the slope of the linear equation given in the graphs should ideally be around 0.32.

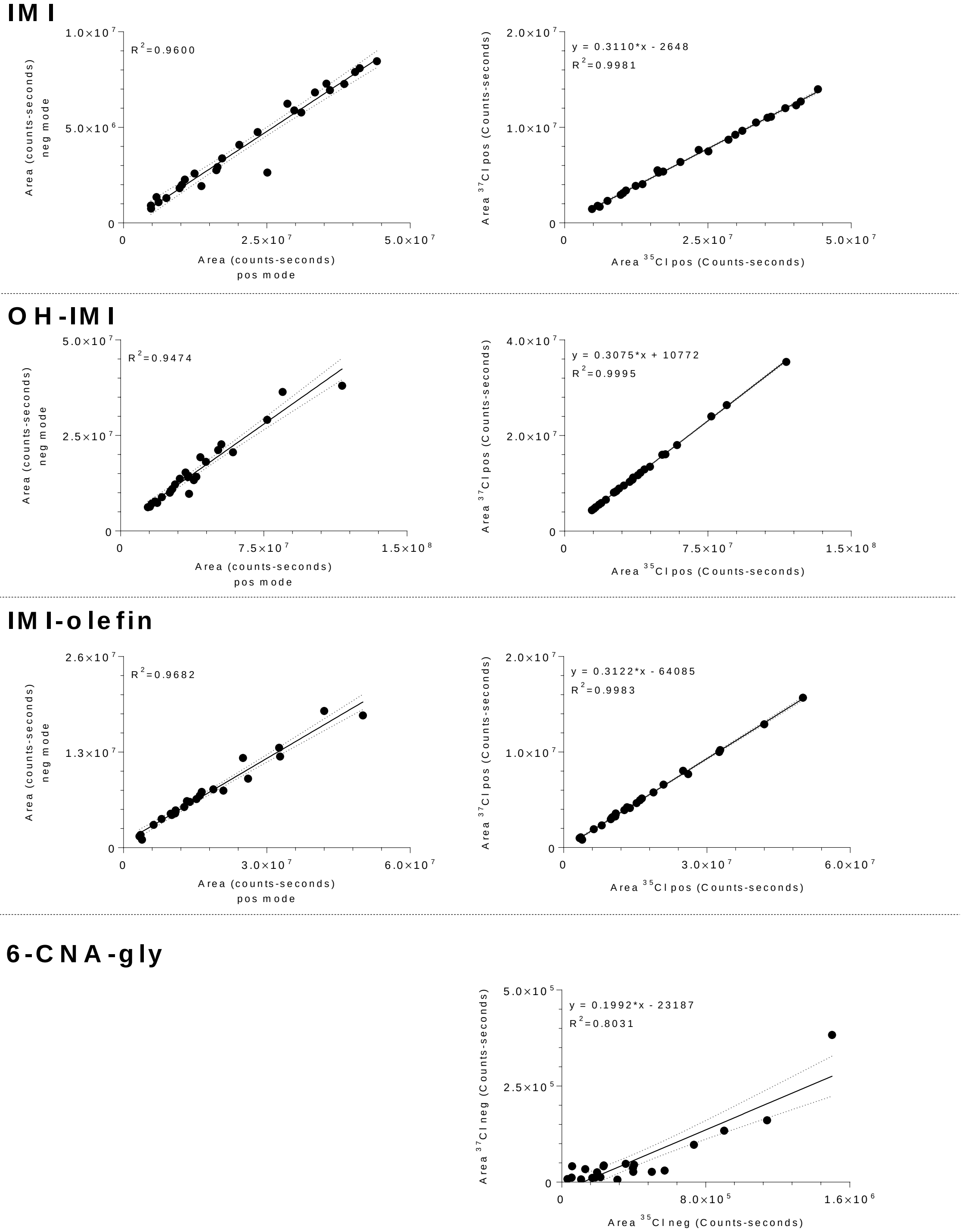

Figure S3b: Pearson correlation between the peak areas in the positive and the negative ionization mode if the corresponding accurate mass was detectable in both ionization modes (left column) and pearson correlation between the peak areas of the $^{35}Cl/^{37}Cl$-isotopologs either from positive or in negative ionization mode for thiacloprid (THIAC), hydroxy-THIAC-olefin (OH-THIAC-olefin), and OH-THIAC olefin obtained by LC-Q-Orbitrap-MS analysis after oral dose of 1 mg thiacloprid. Based on the relative abundance of the ^{35}Cl- and ^{37}Cl-isotopologs, the slope of the linear equation given in the graphs should ideally be around 0.37 due to a mass difference of <5 ppm between ^{37}Cl and ^{34}S which cannot be resolved.

THIAC

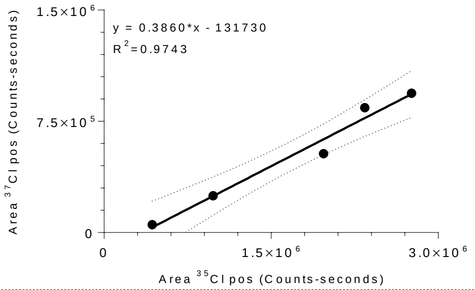

OH-THIAC-olefin

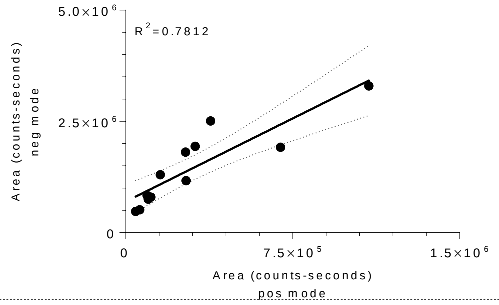

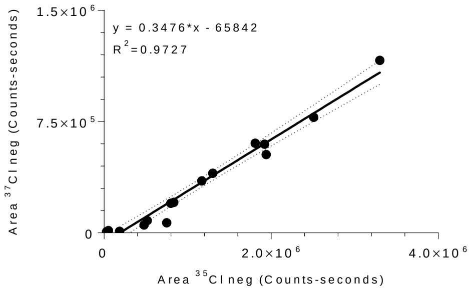

OH-THIAC 8.8 + 8.9 min

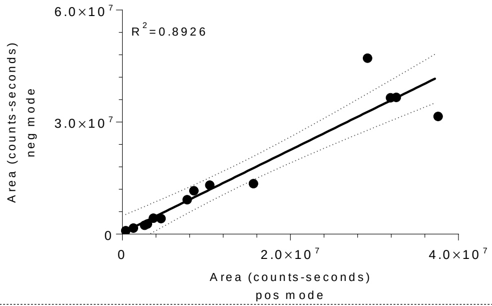

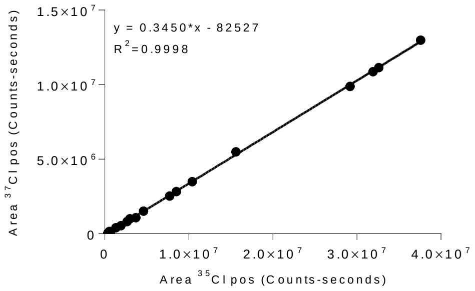

OH-THIAC 10.5 min

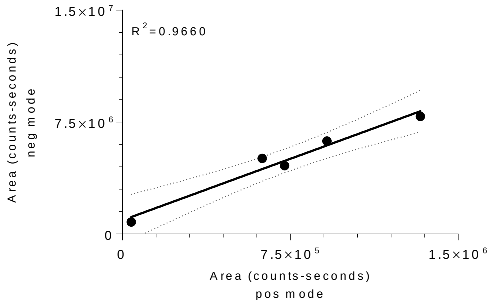

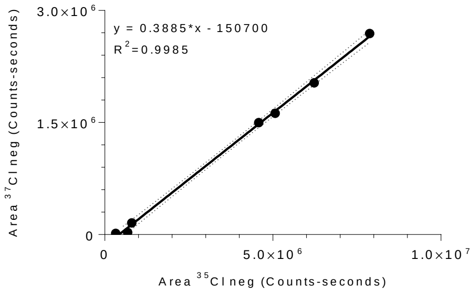

Figure S3b: continued.

THIAC-amide

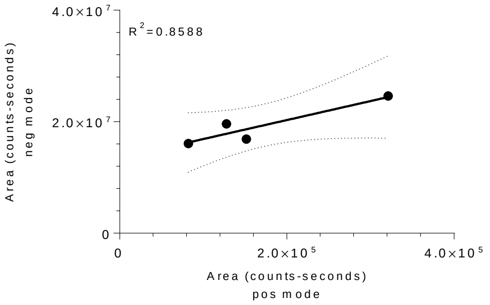

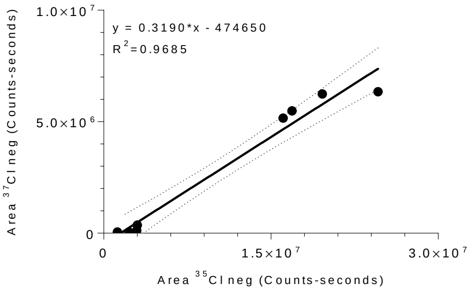

6-CNA-gly

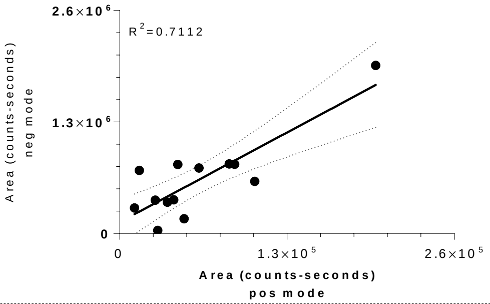

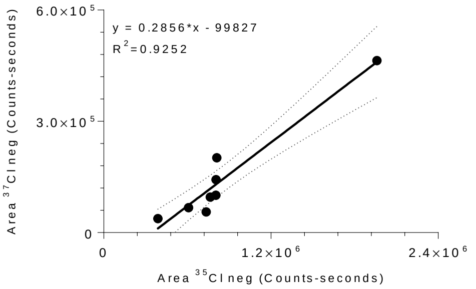

C11H15ClN4O3S

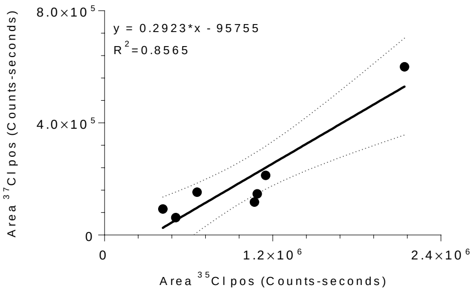

Electronic Supplementary Material (Wrobel et al. 2021)

Figure S3c: Pearson correlation between the peak areas in the positive and the negative ionization mode if the corresponding accurate mass was detectable in both ionization modes (left column) and pearson correlation between the peak areas of the $^{35}Cl/^{37}Cl$-isotopologs either from positive or in negative ionization mode for acetamiprid (ACE), desmethyl-ACE (DME-ACE), and 6-chloro nicotinoyl glycine (6-CNA-gly) obtained by LC-Q-Orbitrap-MS analysis after oral dose of 2 mg acetamiprid. Based on the relative abundance of the ^{35}Cl- and ^{37}Cl- isotopologs, the slope of the linear equation given in the graphs should ideally be around 0.32.

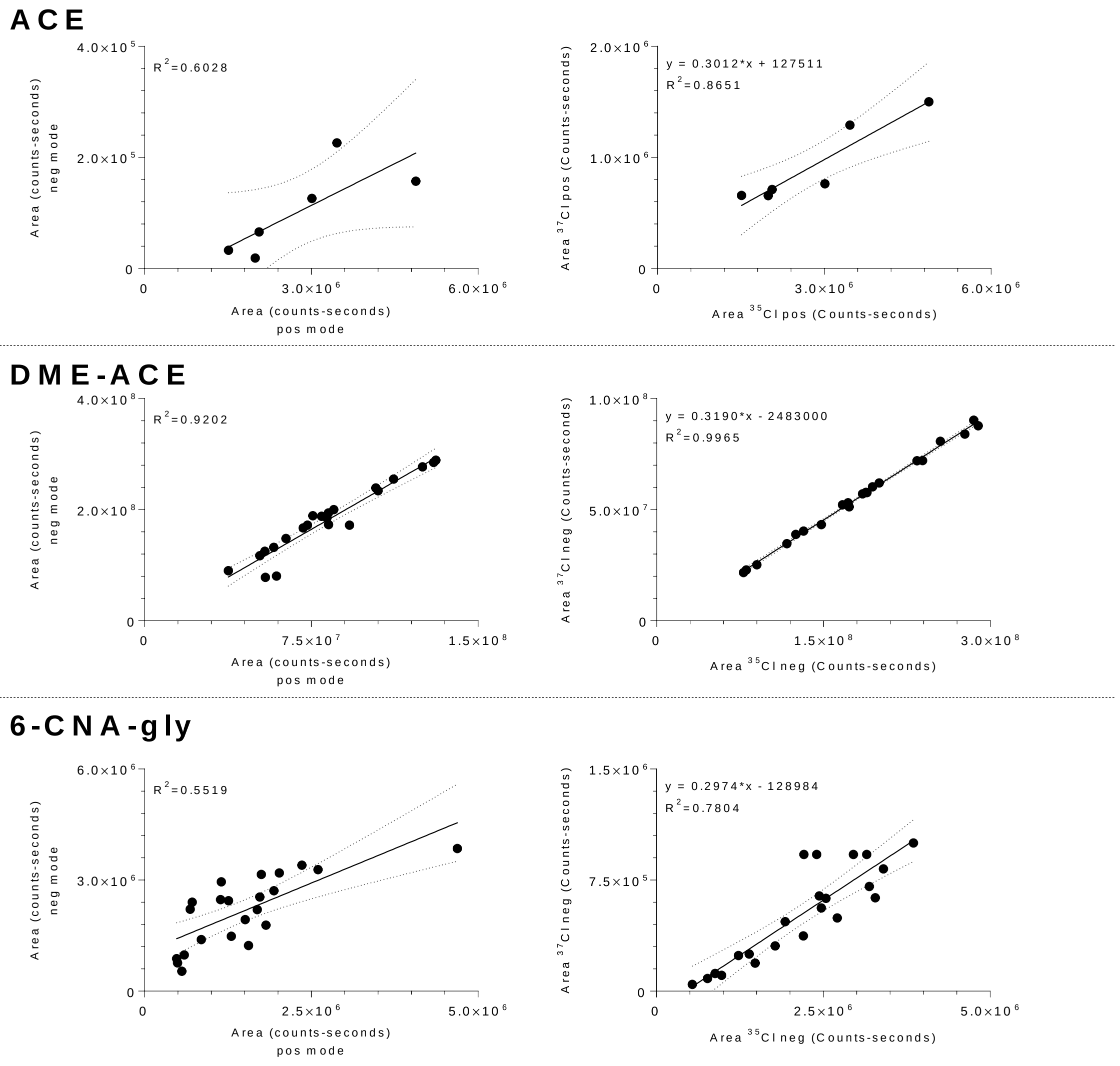

Figure S3d: Pearson correlation between the peak areas in the positive and the negative ionization mode if the corresponding accurate mass was detectable in both ionization modes (left column) and pearson correlation between the peak areas of the $^{35}Cl/^{37}Cl$-isotopologs either from positive or in negative ionization mode for thiamethoxam (THIAM), clothianidin (CLO), desmethyl-CLO (DME-CLO) and cysteinyl-THIAM obtained by LC-Q-Orbitrap-MS analysis after oral dose of 2 mg thiamethoxam. Based on the relative abundance of the ^{35}Cl- and ^{37}Cl- isotopologs, the slope of the linear equation given in the graphs should ideally be around 0.37 due to a mass difference of <5 ppm between ^{37}Cl and ^{34}S which cannot be resolved. For cysteinyl-THIAM, the isotope relation between the ^{32}S- and ^{34}S should ideally result in a slope of approximately 0.045.

THIAM

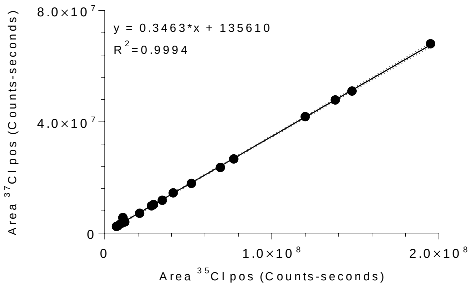

CLO

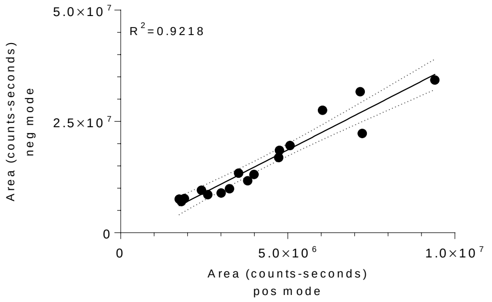

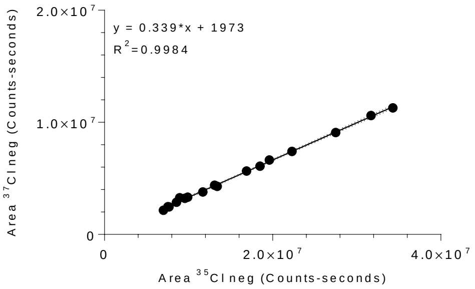

DME-CLO

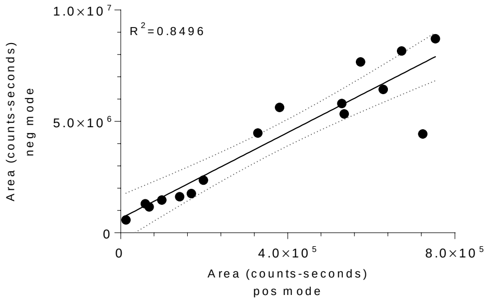

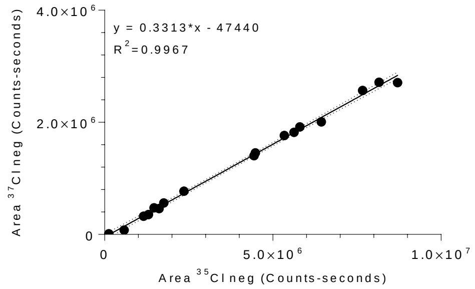

Cysteinyl-THIAM

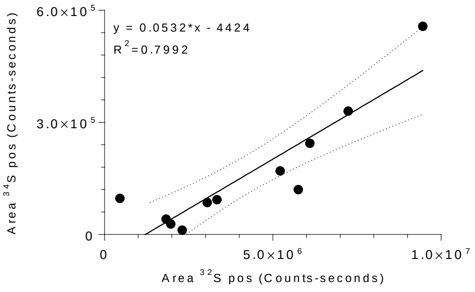

Figure S3e: Pearson correlation between the peak areas in the positive and the negative ionization mode if the corresponding accurate mass was detectable in both ionization modes (left column) and pearson correlation between the peak areas of the $^{35}Cl/^{37}Cl$-isotopologs either from positive or in negative ionization mode for clothianidin (CLO), desmethyl-CLO (DME-CLO), CLO-urea, and cysteinyl-CLO obtained by LC-Q-Orbitrap-MS analysis after oral dose of 5 mg clothianidin. Based on the relative abundance of the ^{35}Cl- and ^{37}Cl-isotopologs, the slope of the linear equation given in the graphs should ideally be around 0.37 due to a mass difference of <5 ppm between ^{37}Cl and ^{34}S which cannot be resolved.

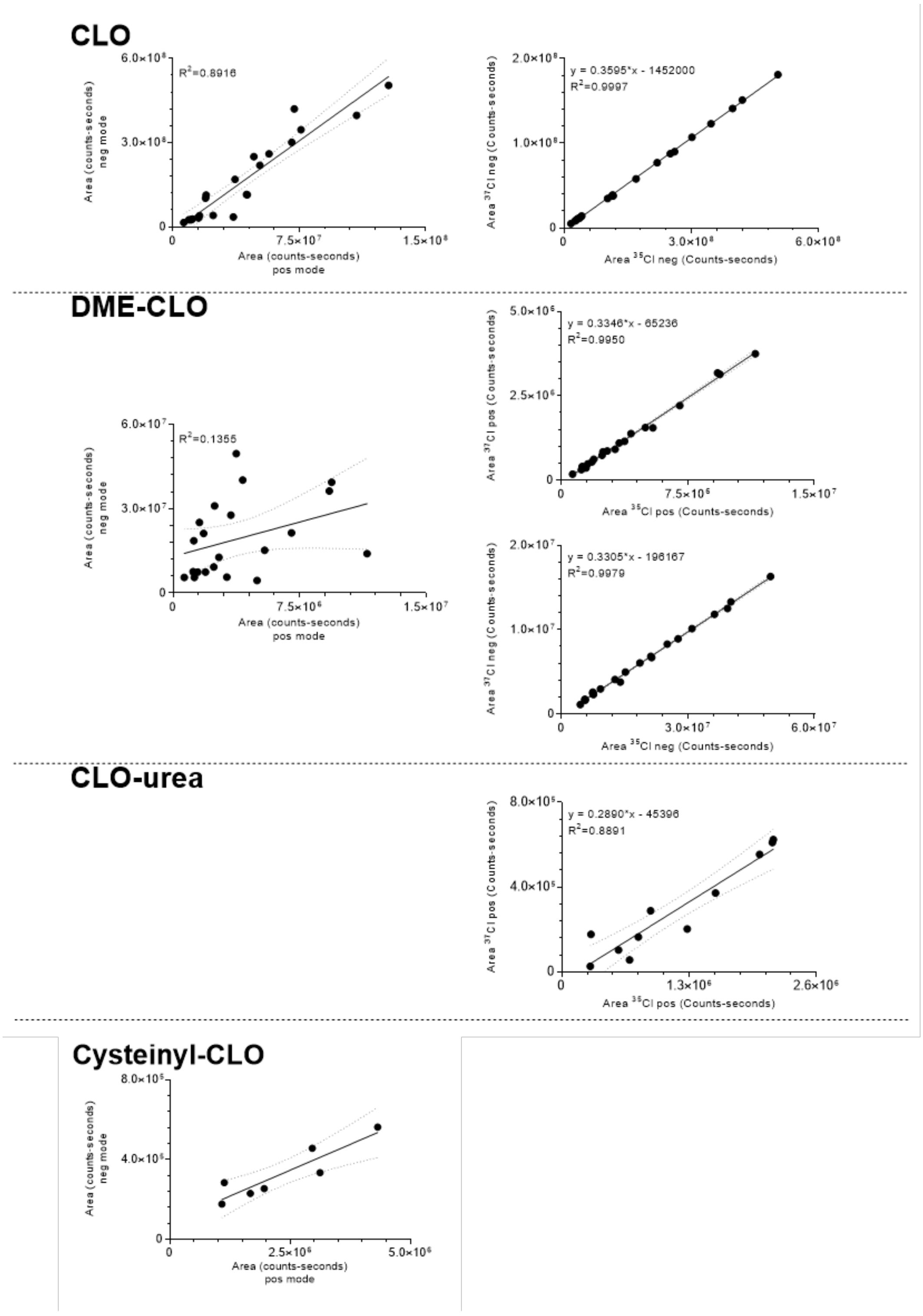

Figure S3f: Pearson correlation between the peak areas in the positive and the negative ionization mode if the corresponding accurate mass was detectable in both ionization modes (left column) and pearson correlation between the peak areas of the $^{35}Cl/^{37}Cl$-isotopologs either from positive or in negative ionization mode for flupyradifurone (FLUP) and three hydroxy-FLUP (OH-FLUP) isomers obtained by LC-Q-Orbitrap-MS analysis after oral dose of 5 mg flupyradifurone. Based on the relative abundance of the ^{35}Cl- and ^{37}Cl- isotopologs, the slope of the linear equation given in the graphs should ideally be around 0.32.

FLUP

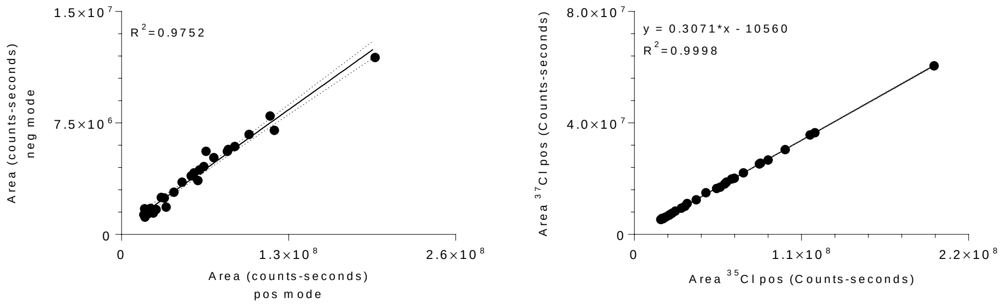

OH-FLUP 9.2 min

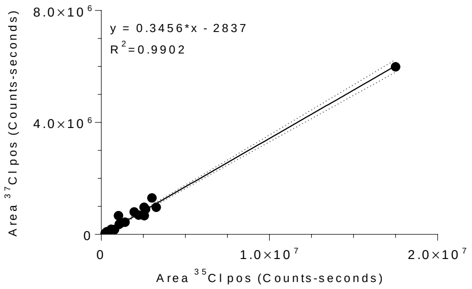

OH-FLUP 9.3 min

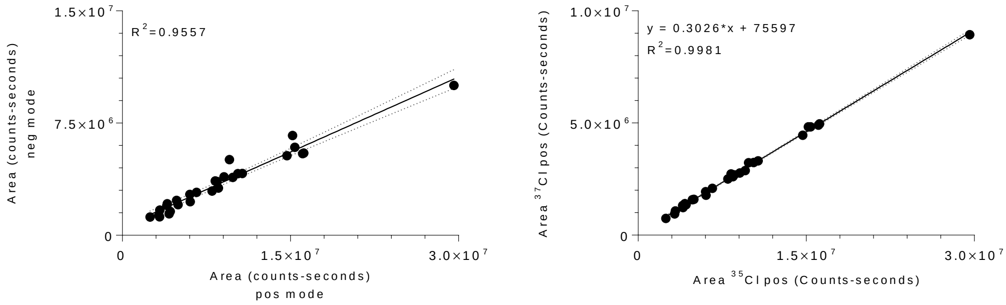

OH-FLUP 9.8 min

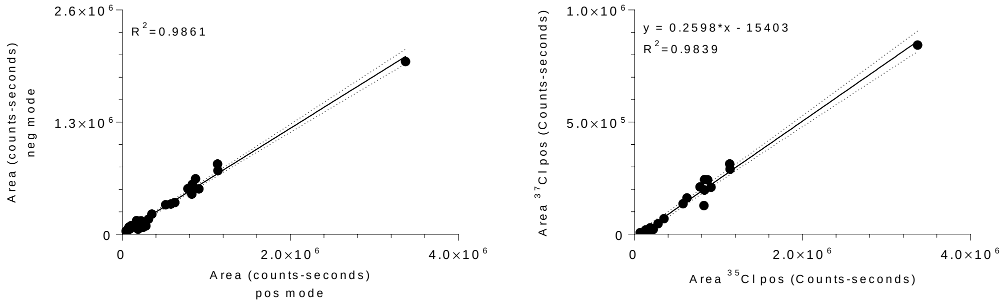

Figure S3f: continued.

DFE-FLUP (ESI+)

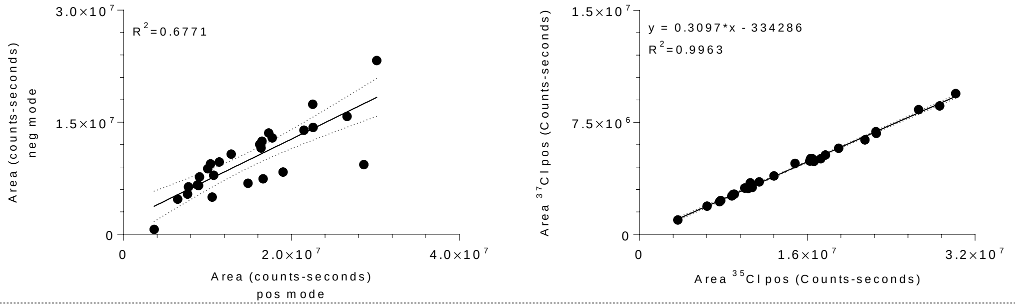

6-CNA-gly (ESI-)

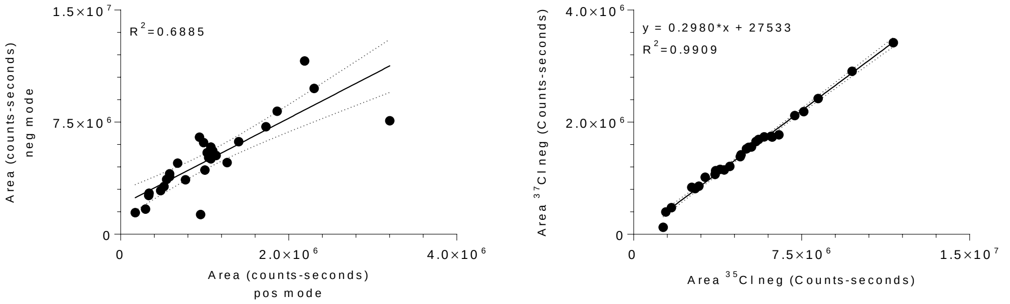

Figure S3g: Pearson correlation between the peak areas in the positive and the negative ionization mode (left column) and pearson correlation between the peak areas of the two sulfur ^{32}S/^{34}S-isotopes from the negative ionization mode for sulfoxaflor (SULF) after oral dose of 3 mg obtained by LC-Q-Orbitrap-MS analysis. Based on the relative abundance of the ^{32}S- and ^{34}S- isotopologs, the slope of the linear equation given in the graphs should ideally be around 0.045.

SULF

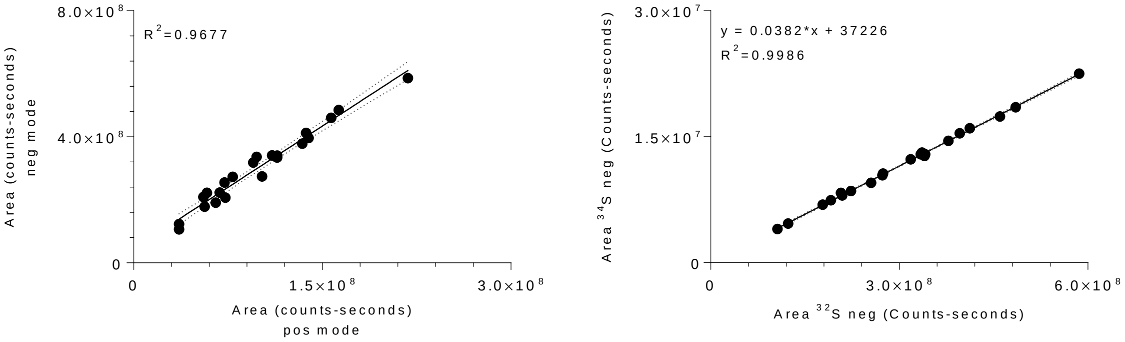

Figure S4: Artefactual formation of desnitro-IMI-olefin due to potential in-source fragmentation of IMI shown for a neat standard solution of 50 µg/L in water.

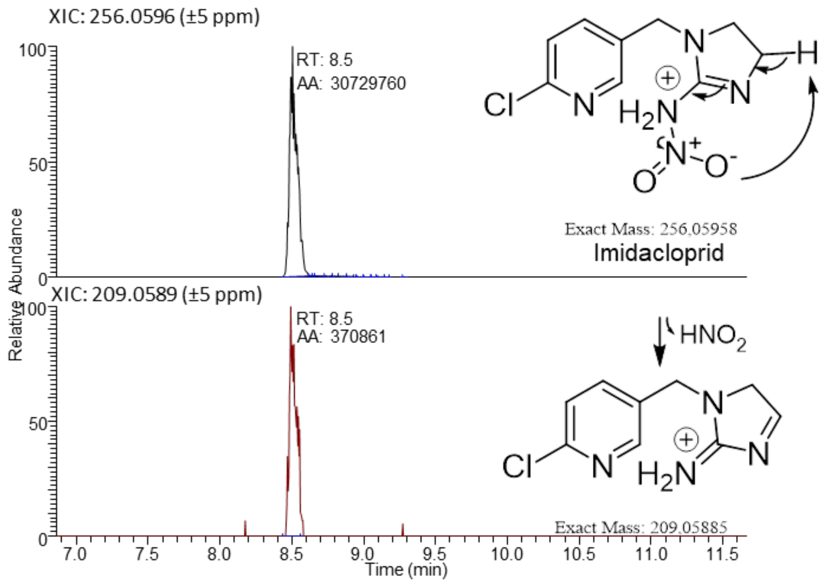

Figure S5: (**A**) Extracted ion chromatogram of 6-chloronicotinic acid (6-CNA) in the negative ionization mode (m/z 155.98578 ± 5 ppm) and (**B**) its corresponding product ion spectrum of a standard solution of 6-CNA in water (50 µg/L). (**C**) Extracted ion chromatograms (negative ionization mode) for the same mass of the first eight urine samples (t = 0-5.8 h) after an oral dose of 5 mg imidacloprid. No peaks for 6-CNA after dosing could be observed. (**D**) Extracted ion chromatograms of blank urine which was spiked with 6-CNA up to 25 µg/L and treated and analyzed in the same way as the urine samples from the dose studies. The lowest concentration which could be detected was 10 µg/L.

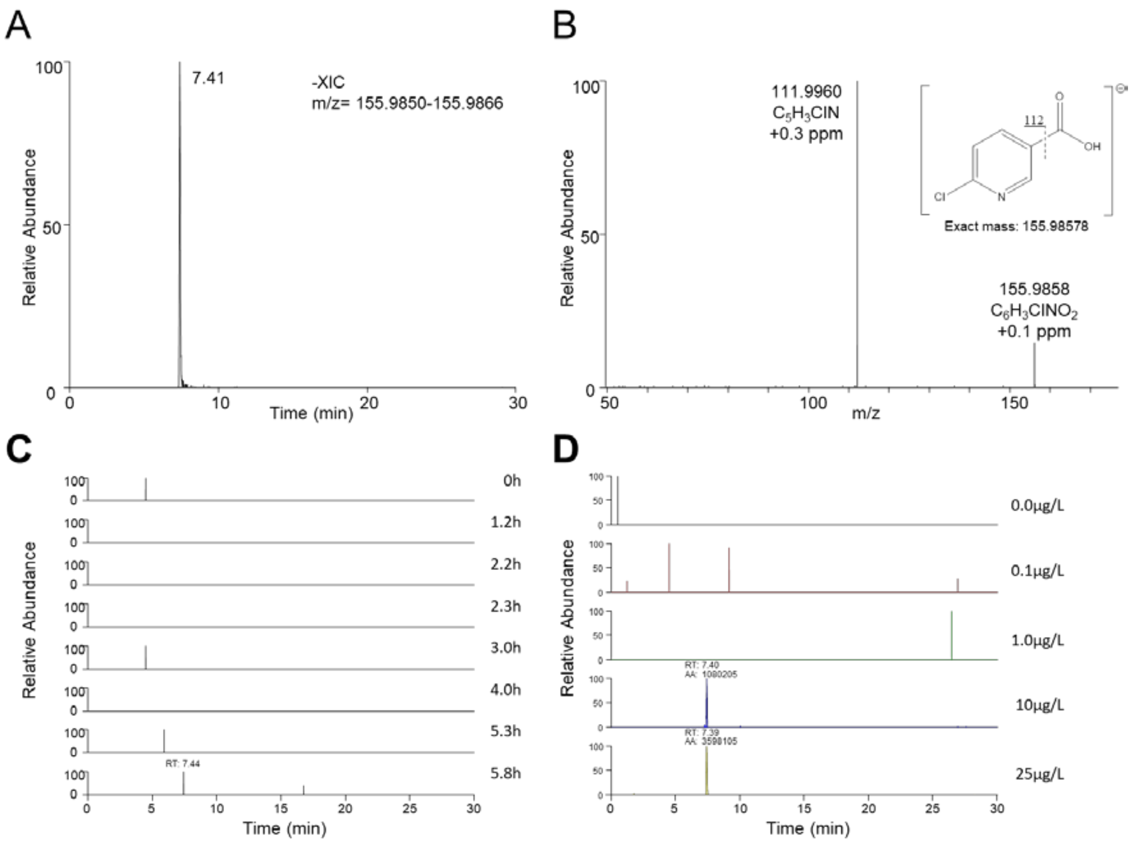

Figure S6: Molecular structures of ”THIAC+O”: OH-group either at C4- or C5-position of the 1-H-thiazol moiety (OH-THIAC; **1**), THIAC-amide-olefin (**2**) and THIAC-sulfoxide (**3**) (see main text).

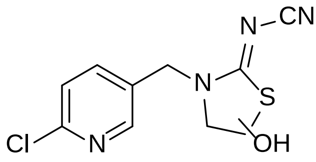

1
Chemical Formula: $C_{10}H_9ClN_4OS$
Exact Mass: 268.01856

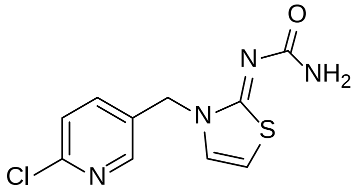

2
Chemical Formula: $C_{10}H_9ClN_4OS$
Exact Mass: 268.01856

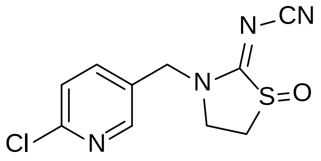

3
Chemical Formula: $C_{10}H_9ClN_4OS$
Exact Mass: 268.01856

Figure S7: Extracted ion chromatogram (MS^1 full scan data) in positive ionization mode for potentially three hydroxylated thiacloprid isomers (OH-THIAC, *m/z* 269.02584 ±5 ppm) 2.3 h after an oral dose of 1 mg of THIAC (see main text).

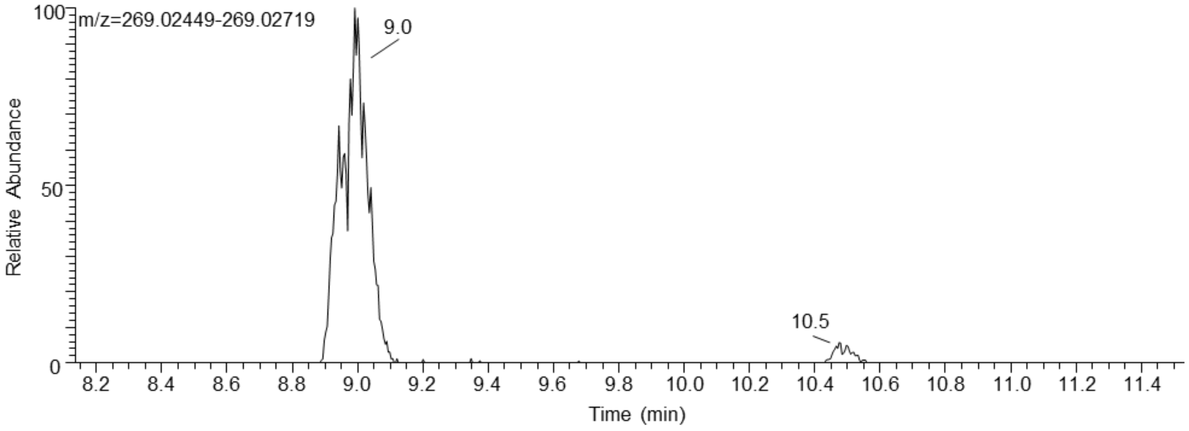

Figure S8: (**A**) Extracted ion chromatogram of 2-chlorothiazole-4-carboxylic acid (2-CTA) in the negative ionization mode (*m/z* 161.94220 ± 5 ppm) and (**B**) its corresponding product ion spectrum of a standard solution of 2-CTA in water (50 µg/L). (**C**) Extracted ion chromatograms (negative ionization mode) for the same mass of the first eight urine samples (t = 0-12.4 h) after an oral dose of 5 mg clothianidin. No peaks for 2-CTA after dosing could be observed. (**D**) Extracted ion chromatograms of blank urine which was spiked with 2-CTA up to 25 µg/L and treated and analyzed in the same way as the urine samples from the dose studies. The lowest concentration which could be detected was 10 µg/L.

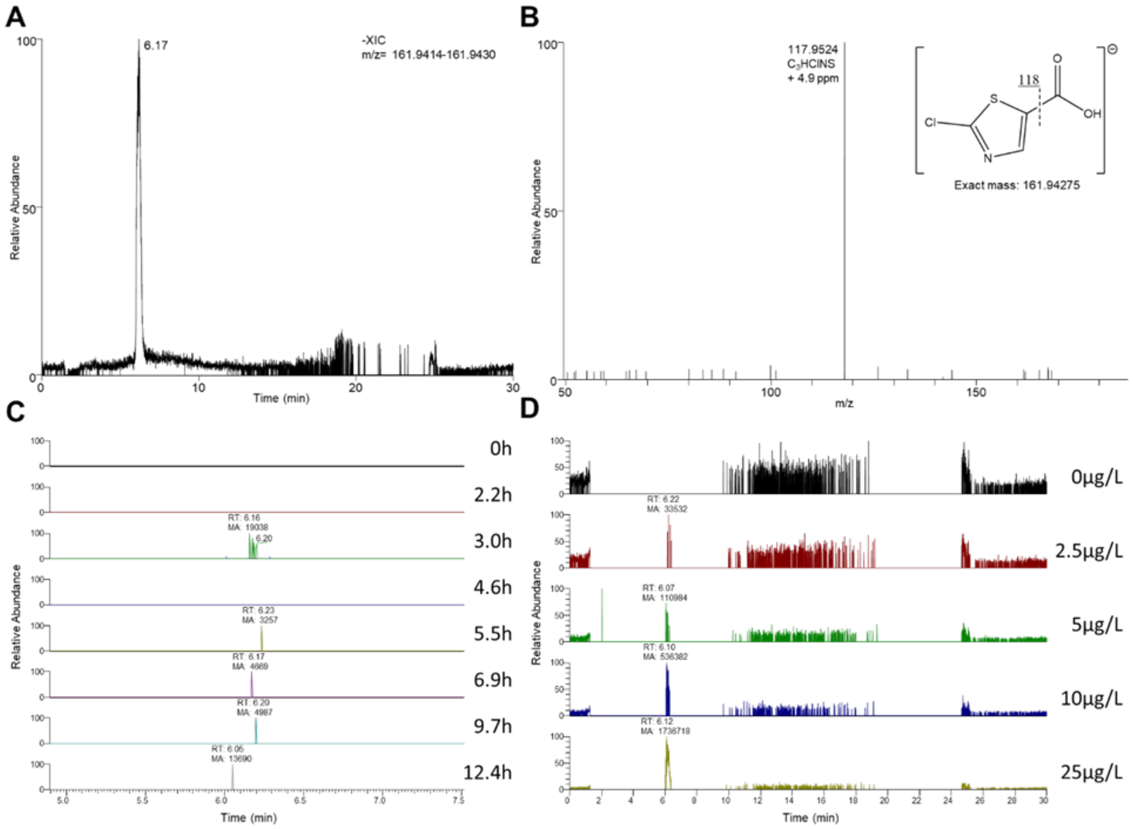

Figure S9: Extracted ion chromatogram (MS[1] full scan, ESI positive) for three hydroxy-flupyradifurone isomers (OH-FLUP; *m/z* 305.04995 ± 5 ppm) at 2.9 h after dosing 5 mg of FLUP (see main text).

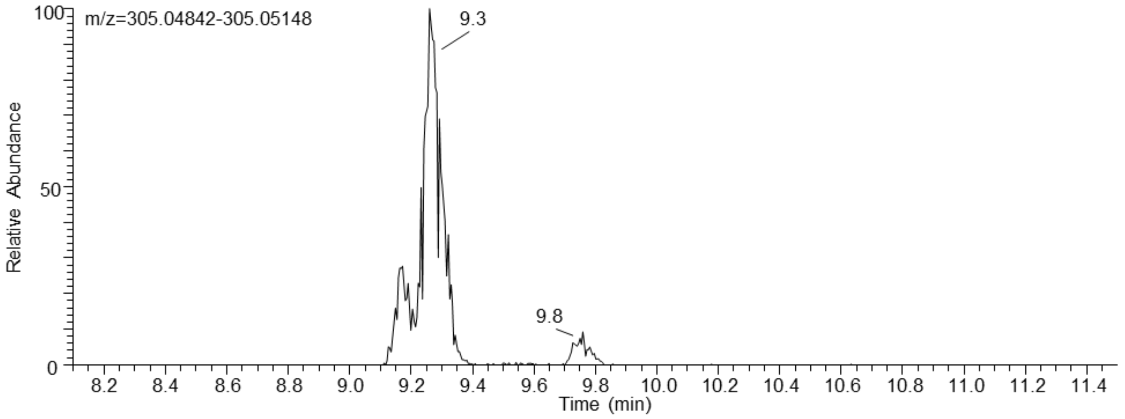

Figure S10: Extracted ion chromatograms (MS^1 full scan data in negative ionization mode) for flupyradifurone (FLUP; *m/z* = 287.04044 ±5 ppm; top), desdifluoro-ethyl-FLUP (DFE-FLUP; *m/z* = 223.02798 ±5 ppm; middle) and deschloropyridinyl-FLUP (DCP-FLUP; *m/z* = 162.03721 ±5 ppm; bottom) at t_{18} = 23h after dosing 5 mg of FLUP (see main text).

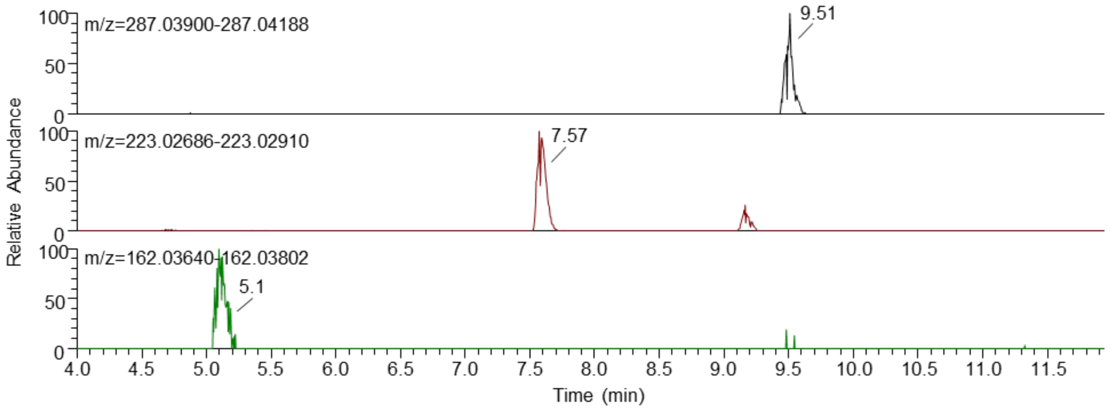

Supplement: Supplementary file 1 — Supplementary file1 (PDF 1895 KB) [file 204_2021_3159_MOESM1_ESM.pdf]
